# Transcriptomic dynamics in the transition from ground to space are revealed by Virgin Galactic human-tended suborbital spaceflight

**DOI:** 10.1038/s41526-023-00340-w

**Published:** 2023-12-20

**Authors:** Robert J. Ferl, Mingqi Zhou, Hunter F. Strickland, Natasha J. Haveman, Jordan B. Callaham, Sirisha Bandla, Daniel Ambriz, Anna-Lisa Paul

**Affiliations:** 1 Department of Horticultural Sciences, University of Florida, 2550 Hull Road, Fifield Hall, Gainesville, FL 32611 USA; 2 UF Research, University of Florida, 1523 Union Rd, Grinter Hall, Gainesville, FL 32611 USA; 3 Plant Molecular and Cellular Biology Program, University of Florida, 2550 Hull Road, Fifield Hall, Gainesville, FL 32611 USA; 4 Virgin Galactic, 1700 Flight Way, 3rd Floor, Tustin, CA 92782 USA; 5 Interdisciplinary Center for Biotechnology Research, University of Florida, 2033 Mowry Road, Gainesville, FL 32610 USA

**Keywords:** Plant sciences, Molecular biology

## Abstract

The Virgin Galactic Unity 22 mission conducted the first astronaut-manipulated suborbital spaceflight experiment. The experiment examined the operationalization of Kennedy Space Center Fixation Tubes (KFTs) as a generalizable approach to preserving biology at various phases of suborbital flight. The biology chosen for this experiment was *Arabidopsis thaliana*, ecotype Col-0, because of the plant history of spaceflight experimentation within KFTs and wealth of comparative data from orbital experiments. KFTs were deployed as a wearable device, a leg pouch attached to the astronaut, which proved to be operationally effective during the course of the flight. Data from the inflight samples indicated that the microgravity period of the flight elicited the strongest transcriptomic responses as measured by the number of genes showing differential expression. Genes related to reactive oxygen species and stress, as well as genes associated with orbital spaceflight, were highly represented among the suborbital gene expression profile. In addition, gene families largely unaffected in orbital spaceflight were diversely regulated in suborbital flight, including stress-responsive transcription factors. The human-tended suborbital experiment demonstrated the operational effectiveness of the KFTs in suborbital flight and suggests that rapid transcriptomic responses are a part of the temporal dynamics at the beginning of physiological adaptation to spaceflight.

## Introduction

The development of human-rated commercial suborbital spacecrafts presents the opportunity to understand the biological effects that occur during the transition from 1 g on the earth to microgravity in space. That the environment inside the vehicle and the accelerations of the flight profile are human rated suggest that these vehicles can be utilized as laboratories well suited to both the study of biological processes in early spaceflight adaptation, as well as opportunities for researchers to conduct those experiments in real time during phases of the flight profile. Virgin Galactic (VG) flies a spaceflight system consisting of a spaceship *VSS Unity*, along with a mothership, *VMS Eve*, to perform commercial suborbital flights. This unique platform delivers a suborbital spaceflight profile including a slight hyper-*g* of horizontal takeoff, a hyper-*g* during spaceship’s rocket motor boost to space, a 3–4 min microgravity period, and a re-entry with a mixture of hyper- and hypo-*g* segments prior to landing^1,2^. This platform allows customer-defined scientific experimental materials to be flown as automated hardware payloads, and also opens the door to researchers interacting with payloads to conduct experiments.

This report details the use of Kennedy Space Center Fixation Tubes (KFTs)^2,3^ as suborbital flight rated equipment to house biology in an experimental setup that allows introduction of a fixative solution to the biology during flight through the actions of a flight crew member. Virgin Galactic successfully completed their first fully crewed suborbital spaceflight^4^, the Unity 22 mission, in 2021 with three KFTs as a wearable science payload to be activated by one of the crew.

As a result of the operationalization of KFTs for suborbital science, the report details transcriptomic responses of plants to different phases of the flight profile. Plants have evolved complex responsive signaling in adaptation to environmental dynamic, including the adaptation to extended spaceflight^5–11^. Biological research on plants grown in spaceflight-associated environments is critical to long-term human space missions. Meanwhile, current understanding of plant spaceflight adaptation processes is derived from molecular experiments, primarily gene expression studies, conducted on plants after having been grown for days in orbit. The collective data are therefore rich in what might be considered the steady state levels of plant gene expression on orbit. However, stress adaptation responses of plants are seldom linear over time, and the ultrafast engagement of molecular networks can lead to subsequent systemic alteration of metabolism^12^. Knowledge of rapid gene expression adjustment of plants during the transition from the earth to successful spaceflight adaptation is therefore extremely limited.

## Results

### Setup of human-tended VG Unity 22 mission

**Fig. 1:**
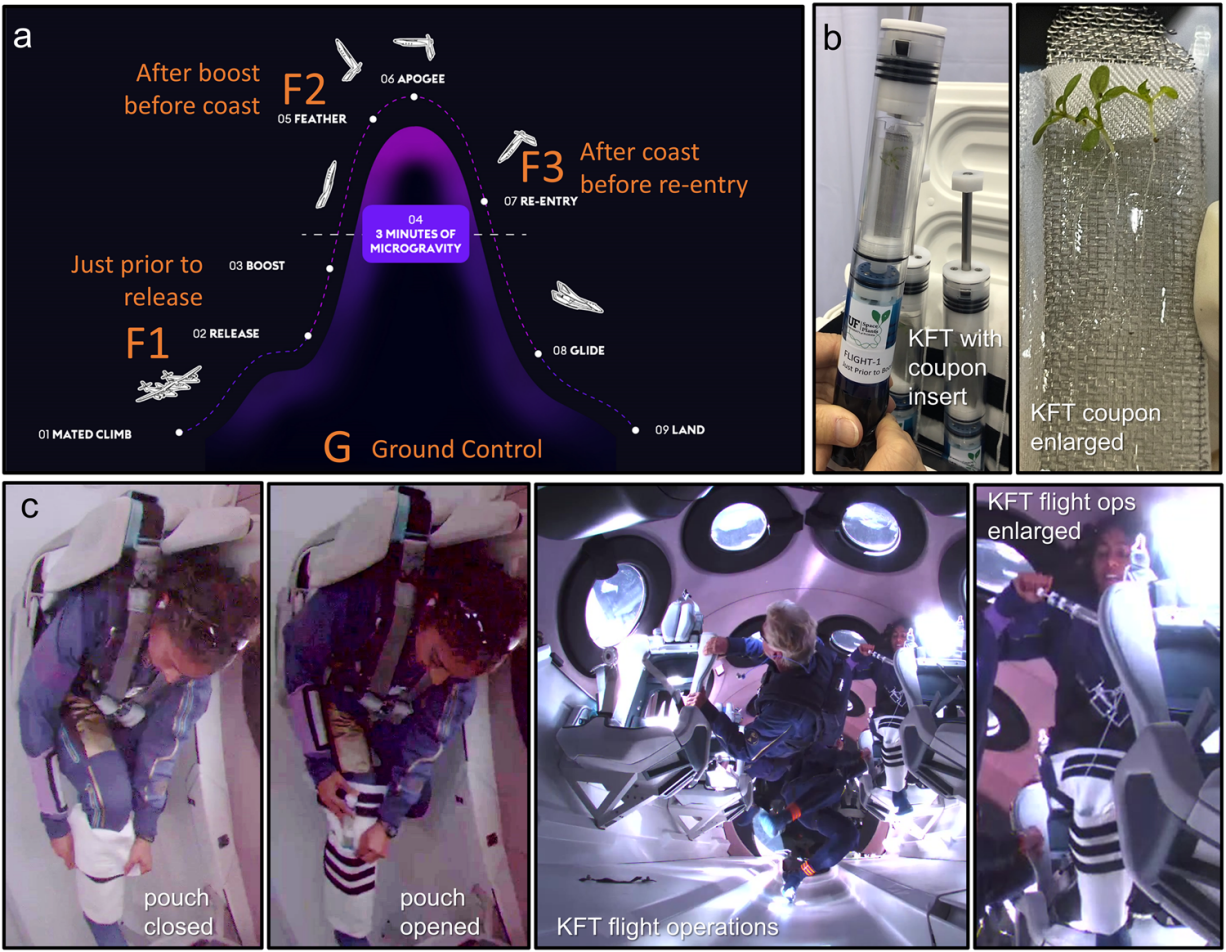
Profile and operation of the flight. **a** A graphic representation of the flight profile showing the points along the profile in which the seedlings were fixed in KFT by the astronaut at F1, F2 and F3. **b** A representative KFT showing the coupon insert holding Arabidopsis 7-day old seedlings; an enlargement of the coupon with seedlings shown to the right. **c** In flight operations of the astronaut showing the configuration of the leg pouch containing the KFTs and steps in the KFT actuation. The center panel shows a wider view of the vehicle interior. The people featured in the figure consent to their inclusion in the images. Co-author, Sirisha Bandla, is the identifiable astronaut manipulating the pouch and KFT in (**c**).

The VG Unity 22 suborbital spaceflight profile is shown in Fig. 1a. Plant materials were fixed at three time points, including F1 (During the climb phase of the spaceflight system—~4 min before release of spaceship from mothership), F2 (the end of hyper-*g* and the beginning of approximate 3-min microgravity), and F3 (the end of 3-min microgravity). These three timepoints bracket major transitions from ground to space. *Arabidopsis thaliana*, Col-0 (Arabidopsis) seedlings were well settled onto agar-coated mesh coupons and sealed in the KFT-supported environment as previously described^2^ (Fig. 1b and Supplementary Video 1). KFTs were actuated (which brings the fixative into contact with the biology) by the suborbital astronaut at the three designated time points (Fig. 1c).

The KFTs were deployed as a wearable payload attached to the astronaut’s calf for easy-access during the flight (Fig. 1c, insert). The loaded KFTs were secured within the pouch by the astronaut just prior to being deployed to the vehicle. Each KFT remained secured in the pouch until astronaut-actuation in turn at F1, F2 and F3, and then re-stowed in turn until their return to the investigators post-flight. Ground Controls were kept in an analog pouch that was worn by one of the investigators, and actuated approximately at the F1, F2 and F3 time points.

### Human-tended suborbital spaceflight revealed phase-dependent transcriptional changes that were not shown in samples collected only at the end of previous flights

**Fig. 2:**
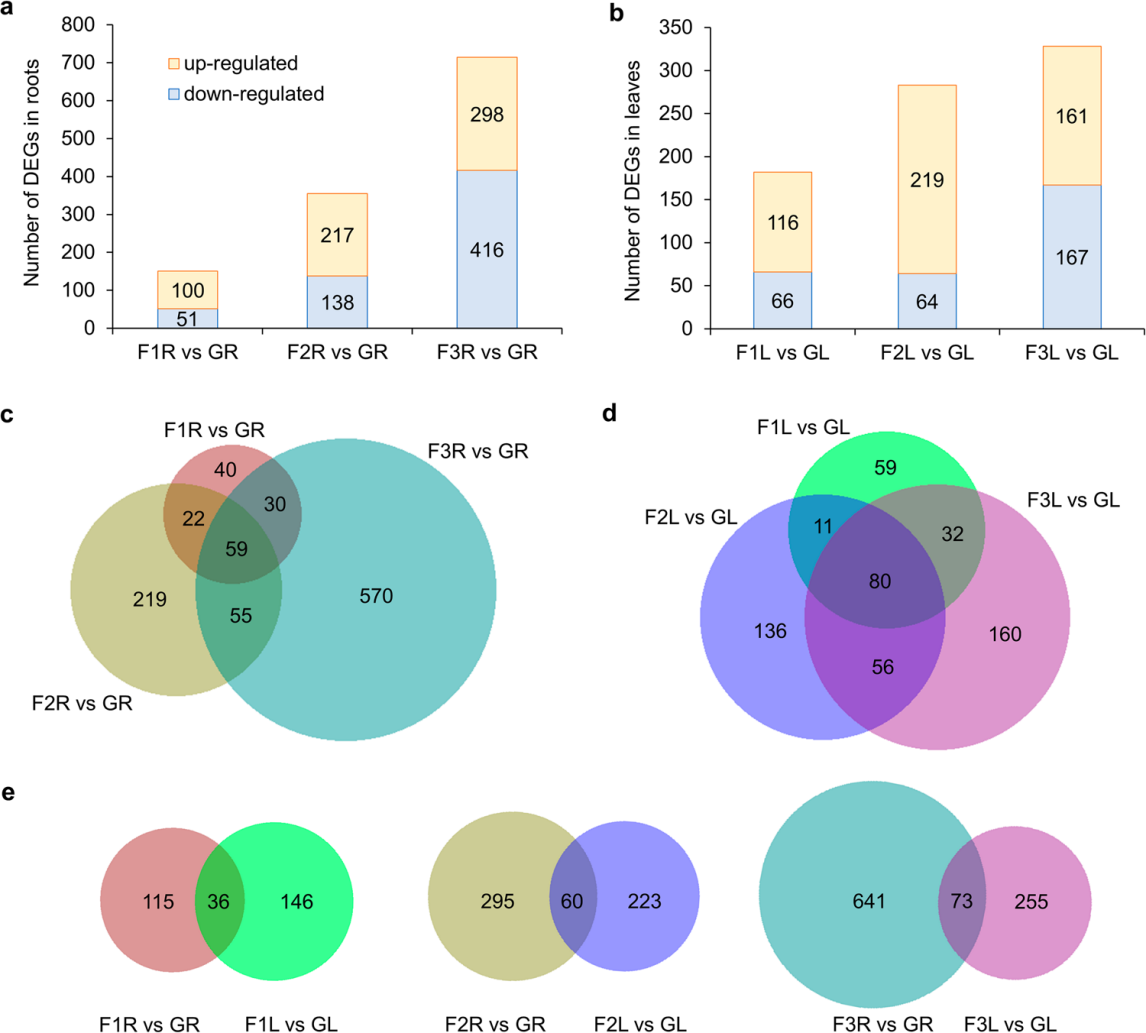
DEGs detected in roots and leaves between Flight and Ground Control. The threshold is Log_2_(fold-change) >1 or <−1, FDR *p*adj <0.05. **a**, **b** Numbers of up-regulated and down-regulated DEGs at time points of F1, F2 and F3 in roots (R) and leaves (L), respectively. **c**, **d** Overlap of DEGs detected at F1, F2 and F3 in roots and leaves, respectively. **e** Overlap of DEGs detected in roots and leaves at each time point of F1, F2 or F3, respectively.

**Fig. 3:**
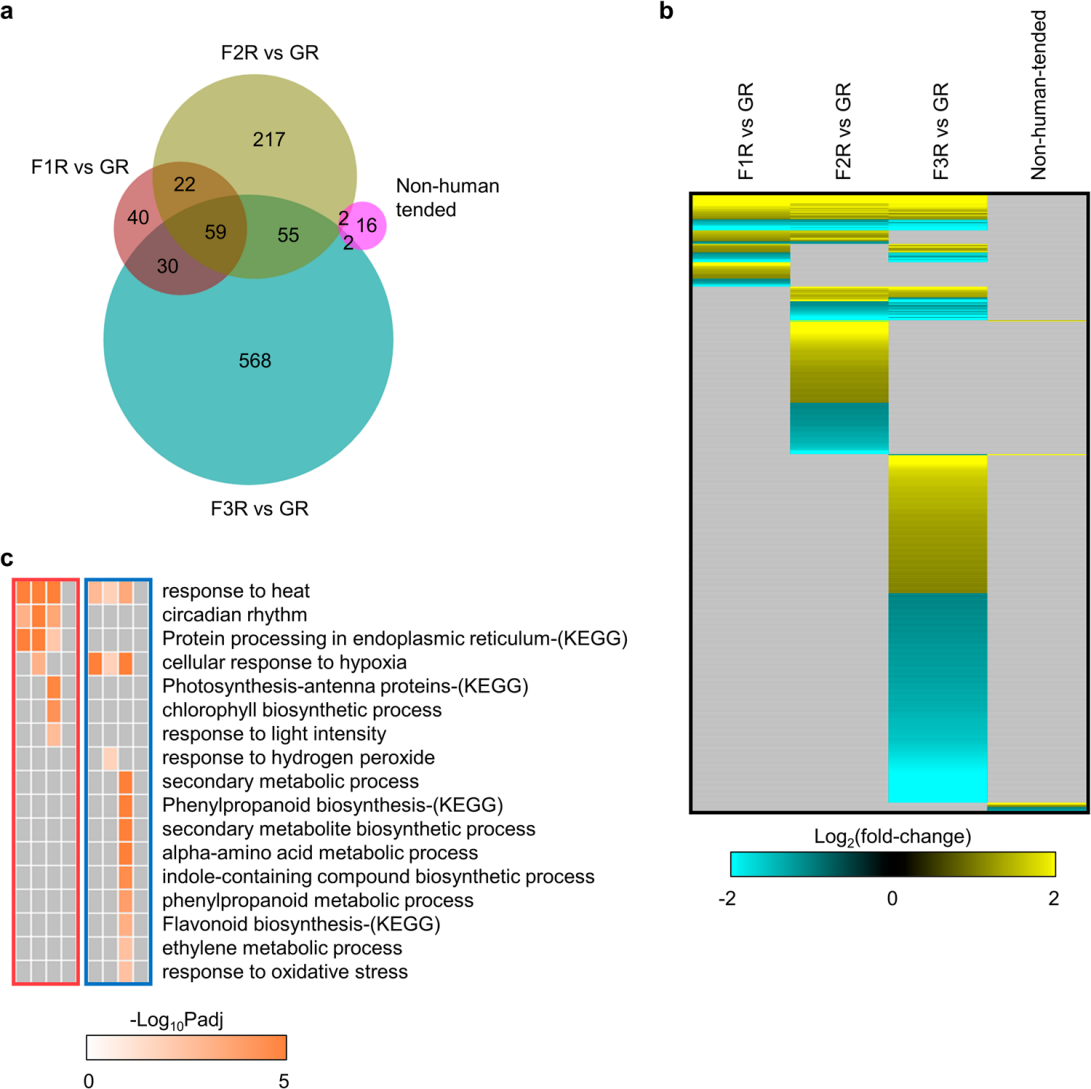
DEGs between Flight and Ground Control in Arabidopsis roots from human-tended and non-human-tended VG suborbital spaceflight experiments. **a** Overlap of DEGs at three time points of human-tended flight and in non-human-tended flight. **b** Expression pattern of DEGs detected in at least one comparison using the threshold of Log_2_(fold-change) >1 or <−1, FDR *p*adj <0.05. Hierarchical clustering of the heatmap is done using one minus cosine similarity. **c** Process and pathway enrichment for DEGs at three time points of human-tended flight and in non-human-tended flight. Biological processes and pathways with FDR *p*adj <0.05 are significantly enriched. The order of the columns is corresponding to that of four comparisons in (**b**). Orange or blue box indicates up-regulated or down-regulated genes in each comparison, respectively.

The differentially expressed genes (DEGs) have been analyzed between VG Unity 22 human-tended suborbital spaceflight samples at three time points and the Ground Control in roots and leaves. In both tissues, F1 showed the smallest number of DEGs and F3 showed the largest (Fig. 2a, b). Consistently, at F3 both roots and leaves exhibited more unique DEGs than that at F1 or F2 (Fig. 2c, d). The overlapped DEGs between roots and leaves at three time points were in a small proportion, lower than 24% in roots and 23% in leaves (Fig. 2e). The DEGs in roots were compared with that of non-human-tended VG VP-03 collected after the completion of a suborbital spaceflight^1^. In VP-03 experiment, 10-day old Arabidopsis seedlings experienced a similar flight pattern as VG Unity 22, while tissues were fixed in RNAlater only when access to payloads was available after landing and transportation of the payload to a harvest station^1^. Only 20 statistically significant DEGs were detected by non-human-tended VP-03 flight in roots, while human-tended VG Unity 22 flight exhibited 151, 355 and 714 DEGs at three time points, respectively (Fig. 3a). Compared with VP-03, VG Unity 22 allowed survey of transcriptome at each time flight stage, therefore revealed phase-dependent gene expression patterns (Fig. 3b and Supplementary Table 1). Using pathway and biological process enrichment analysis, conserved and unique signaling pathways employed by plants in response to each phase of suborbital spaceflight in roots were outlined, while no pathway was significantly enriched for DEGs detected in VP-03 (Fig. 3c). Together, the data suggest human-tended suborbital experiments were able to reveal detailed transcriptional changes for each phase of the transient suborbital spaceflight, which was beyond the capacity of autonomous flight studies.

### Organ-specific expression pattern and gene category of transcriptional regulation in suborbital spaceflight

**Fig. 4:**
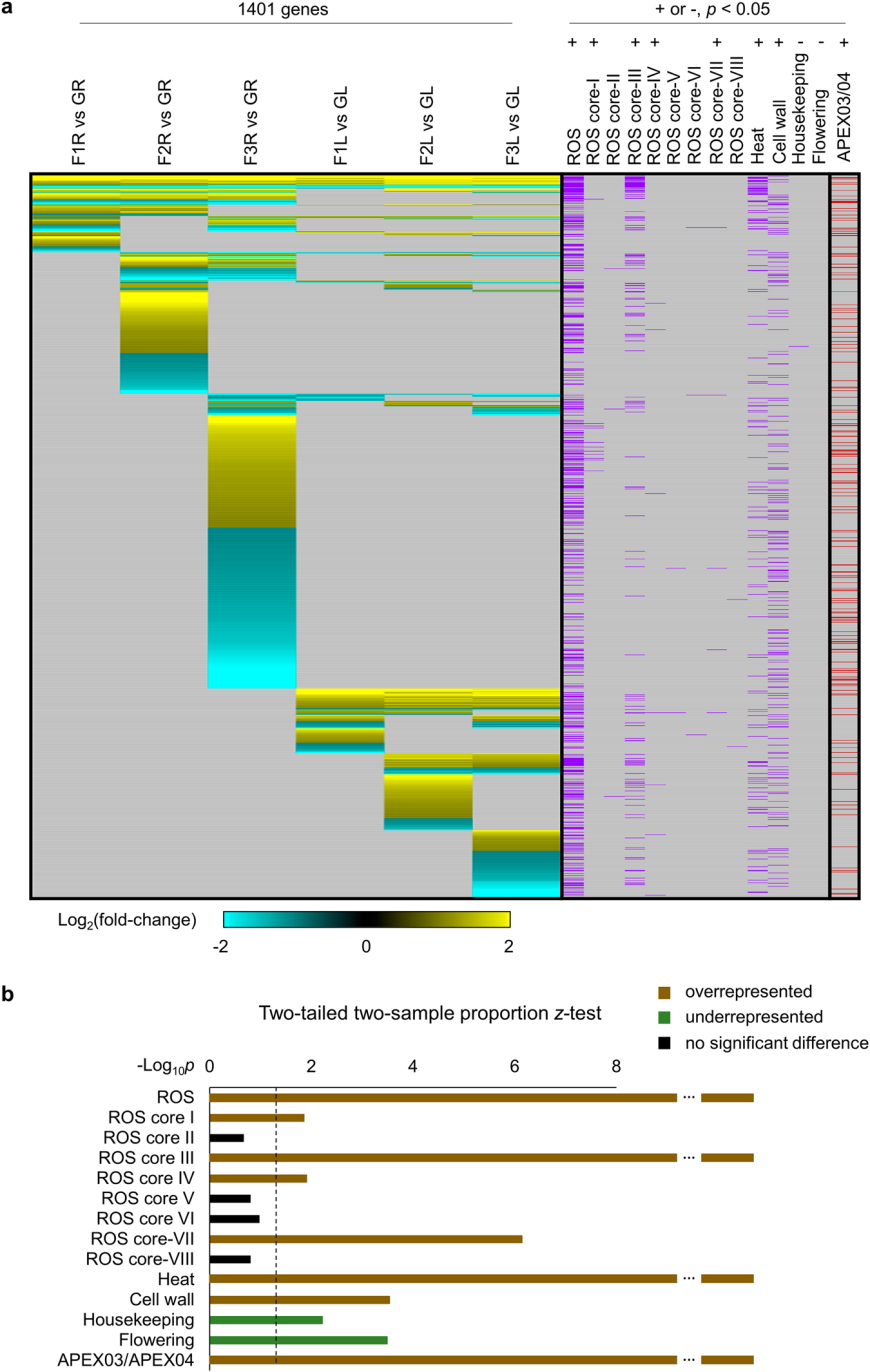
Differential expression pattern of 1401 DEGs between Flight and Ground Control detected in at least one condition. **a** Heatmap showing the up- and down-regulation of DEGs. The threshold is Log_2_(fold-change) >1 or <−1, FDR *p*adj <0.05. Hierarchical clustering of the heatmap is done using one minus cosine similarity. The DEGs belonging to categories of overall ROS related, eight ROS core gene groups, heat response, cell wall development, flowering, housekeeping and APEX03/04 spaceflight related, are indicated by purple or maroon bars as labeled. The description of eight ROS core groups is in Supplementary Table 2. Over- or under-representation of genes from each category in 1401 DEGs are indicated by + and −, respectively. Two-tailed two-sample proportion *z*-test is used, threshold of significant difference is *p* < 0.05. **b** Significance of *z*-test in (**a**) for each gene category. The dot line indicates the significance of *p* = 0.05 in the axis.

To explore the organ-specific transcriptome and signaling induction of plants in response to phases of flight, expression pattern was plotted for 1401 genes that were differentially expressed in at least one comparison out of six conditions of three time points in roots and leaves (Fig. 4a and Supplementary Table 2). In line with the overlap analysis (Fig. 2c–e), only around 2% DEGs were coordinately expressed across all conditions, and each time point showed unique up- and down-regulated genes in both roots and leaves, especially at F2 and F3. The 1401 DEGs were labeled using their expression profiles in reactive oxygen species (ROS) associated biological processes and developmental processes regarding housekeeping, cell wall and flowering, as well as in spaceflight experiments of APEX03-2 (Arabidopsis Col-0 roots)^13^ and APEX04 (Arabidopsis Col-0 roots and leaves)^14^ (Fig. 4a). The overall ROS related genes were extensively detected for differential expression during the whole suborbital flight process, while phase-specific differential expression pattern was observed using subgroups of ROS core genes according to a meta-clustering analysis of ROS transcriptional footprints that were summarized as the ROS Wheel^15^. There were eight categories of transcriptional profiles in the ROS Wheel, defined as eight ROS Wheel Clusters. The ROS core genes were genes differentially expressed in all transcriptome data sets used within each ROS Wheel Cluster^15^, and eight corresponding ROS core gene groups were used for the overrepresentation analysis. ROS core genes in groups I, III, IV and VII were overrepresented in 1401 DEGs of VG Unity 22 suborbital spaceflight. ROS core I genes associated with the genome uncoupled (GUN) mutation retrograde signaling were particularly up-regulated at F3 in roots. The genes related to heat response and cell wall development that were commonly observed in differential transcriptome of spaceflight^5,8,14,16,17^, were also overrepresented in 1401 DEGs. Flowering and housekeeping genes were underrepresented, indicating the specificity of gene regulation in this suborbital flight study. Notably, ROS core III (rapid-response, high light exposure) and heat responsive genes were highly overlapped with DEGs that were detected in all six conditions. In addition, genes differentially expressed in the Arabidopsis (Col-0) plants of APEX03 and APEX04 spaceflight were overrepresented in the VG Unity 22 DEGs as well^13,14^. Genes of overall ROS related, ROS core III, heat responses and APEX03/04 spaceflight showed highest significance for the overrepresentation in the 1401 DEGs (Fig. 4b), demonstrating that these genes involved in long-term spaceflight were also subjected to fast and/or transient transcriptional modulation in suborbital spaceflight.

### Regulation of plant signaling pathways during phases of flight

**Fig. 5:**
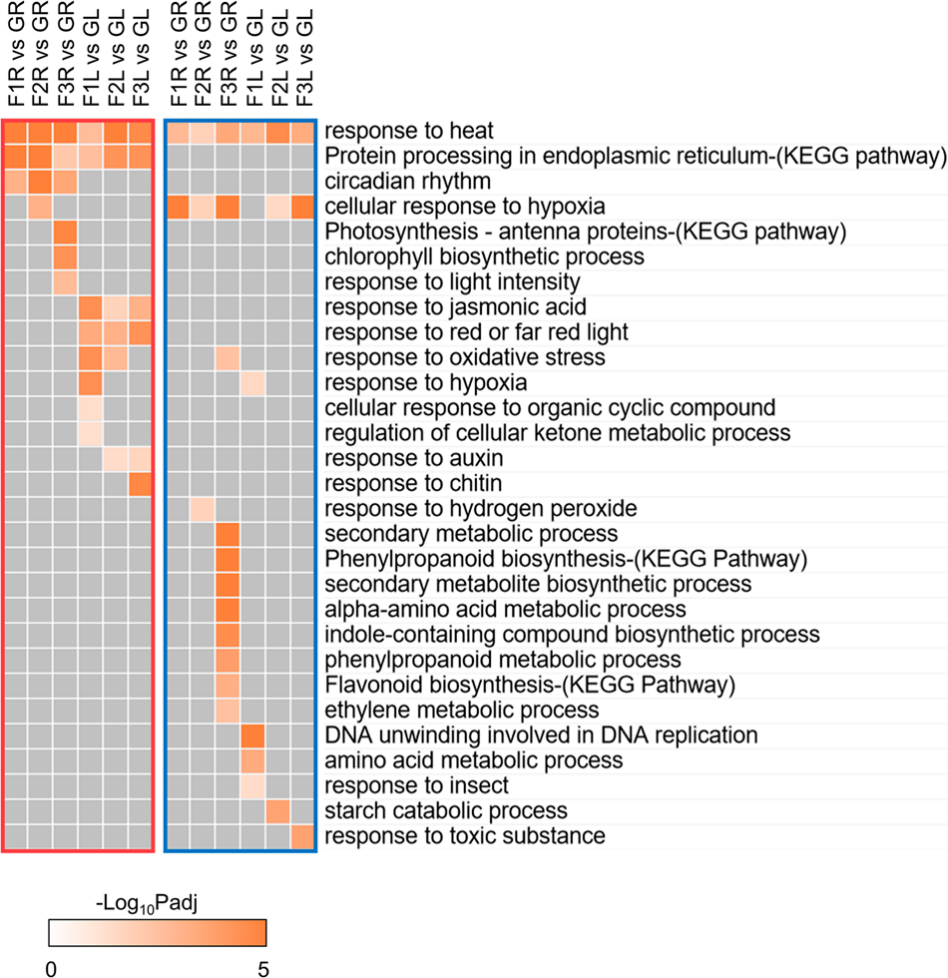
Process and pathway enrichment analysis for DEGs at each time point of VG suborbital spaceflight in roots and leaves. Biological processes and pathways with FDR *p*adj <0.05 are significantly enriched. Orange and blue box indicates up- and down-regulated genes in each condition, respectively.

Biological process and pathway enrichment analysis was performed on DEGs of each condition to further assay plant responses to rapid gravity alteration of suborbital spaceflight (Fig. 5). The heat responsive pathway was shared by up- and down-regulated DEGs of roots and leaves, which was consistent with the gene category analysis (Fig. 4a). Genes associated with signaling were up-regulated across three phases of flight. Genes associated with protein processing in endoplasmic reticulum was enhanced in both organs, while circadian related pathway genes were induced only in roots and jasmonic acid-associated, and red/far red light signaling were induced only in leaves. Hypoxia responsive pathways that are typical of orbital spaceflight responses^18,19^ exhibited diverse regulation of activation and repression at same stages of suborbital spaceflight. At F3 when plants just ended transient microgravity experience, roots showed a robust adjustment of signaling cascades, in which light responsive pathways were enhanced and multiple metabolic processes were decreased. Together, the rapid gravity alteration in VG suborbital spaceflight profile elicited diverse stress responses and metabolic processes in roots and leaves.

### Gene families with diverse expression patterns in plant responses to phases of flight

**Fig. 6:**
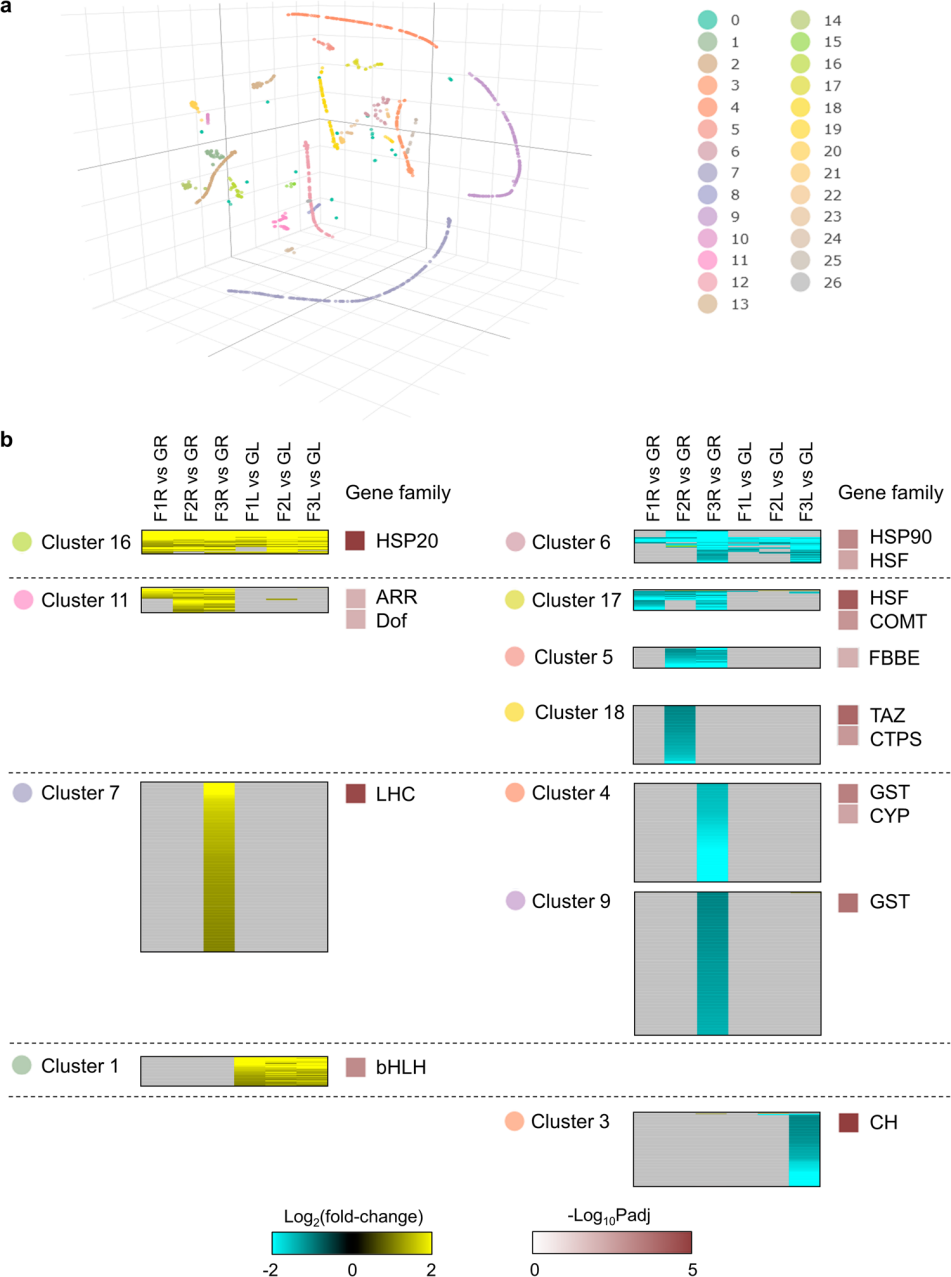
Gene family enrichment analysis through t-SNE clustering. **a** 27 clusters are shown for 1401 DEGs in t-SNE clustering. **b** Gene families significantly enriched for DEGs in 11 clusters. Threshold of significant enrichment is FDR *p*adj <0.01. Differential expression patterns of these DEGs are shown.

To further uncover gene families that were enriched for DEGs detected in each phase of VG suborbital spaceflight, we performed t-distributed stochastic neighbor embedding (t-SNE)-based clustering on 1401 DEGs. In total, 27 clusters were generated according to significant differential expression pattern of DEGs (Fig. 6a). Clusters were marked by phase and/or organ-dependent up- or down-regulation of DEGs (Supplementary Table 2). Enrichment analysis identified representative gene families that were altered in roots and leaves in different phases of suborbital spaceflight for 11 clusters (Supplementary Table 3). In general, more significantly enriched gene families were exhibited in roots than in leaves, and most phase-specific gene groups were observed at the time point of F3, in which plant responses to the microgravity period were surveyed (Fig. 6b). These enriched groups involved genes with differential expression in long-term orbital spaceflight experiments. For instance, heat shock protein (HSP) and heat shock factor (HSF) families that were induced in plants grown in ISS^9,13,14,20–23^ were detected in cluster 16 and 6 with differential gene regulation across all conditions. In addition, chlorophyll a/b-binding light-harvesting complex (LHC) and Glutathione S-transferase (GST) families involved in plant ROS signaling during orbital spaceflight^24–29^ were standout among DEGs uniquely changed at F3.

**Fig. 7:**
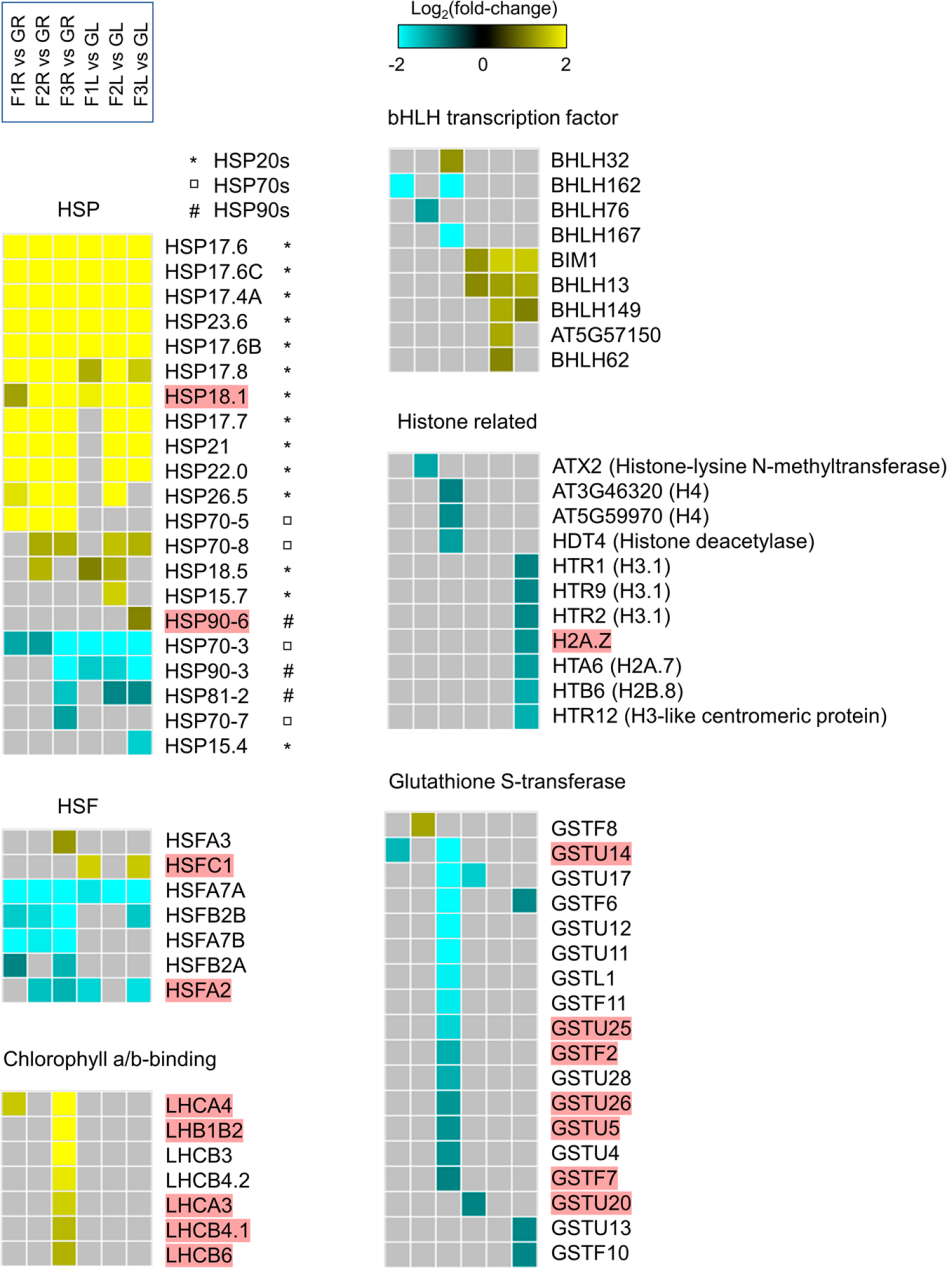
Differential expression patterns of DEGs from selected enriched gene families. Genes belonging to HSP, HSF, Chlorophyll a/b-binding, bHLH, Histone related, and GST families within 1401 DEGs are listed. The order of six comparisons in VG suborbital spaceflight are shown in the box and all gene families listed are using the same order. The genes with differential expression in APEX03/04 spaceflight experiments are highlighted in pink.

After we outlined the gene families showing phase and/or organ-specific differential gene regulation that were identified though clustering, the expression pattern of all genes within these families were pulled out from 1401 DEGs (Fig. 7). For HSPs, three subfamilies including HSP20s, HSP70s and HSP90s were identified. Small HSPs were highly activated in both roots and leaves in the whole process of VG suborbital spaceflight, while HSP70s and HSP90s were repressed extensively in leaves and at F3 in roots. HSFA7, HSFB2 and HSFA2 were also largely reduced in roots. Among these heat related suborbital flight DEGs, 2 HSP and 2 HSF genes were also induced in APEX03/04 orbital flight^13,14^. The light and ROS signaling related LHC genes and ROS scavenger GST genes were specifically activated and repressed at F3 in roots, respectively. There were 5 LHC and 7 GST genes also changed in APEX03/04 experiments. In addition to common genes employed by plants in response to different types of spaceflight, we identified a set of genes that were absent in long-haul orbital spaceflight related transcriptome. For basic helix-loop-helix (bHLH) transcription factors, 1 gene was induced and 3 were down-regulated in roots, while 5 were up-regulated in leaves, and none of them were differentially expressed in APEX03/04 spaceflight. Genes of histone proteins and enzymes associated with their modification were mostly repressed at F3. H4 genes were shown in roots while H2A, H2B and H3 genes were observed in leaves. Among them, only H2A.Z gene was detected in APEX03/04 orbital spaceflight as well.

## Discussion

Suborbital spacecraft, flying to space and back within a short timeframe, offer the potential to examine the biological processes necessary for successful physiological adaptation to space during the process from liftoff to entry into the spaceflight environment. The promise of high flight cadences and reasonable costs for persons to fly further suggests that suborbital flights could be developed into opportunities that allow replicated observations and experiments conducted by researchers. However, since this is still a unique capability, the full complement of operational mechanisms that allow experiments to be conducted by astronauts within their vehicles is being continuously developed and adapted in response to researcher needs. As a result, the types of experiments that are able to be conducted by humans within the suborbital flight parameters are still being developed. This report demonstrates the conduct of an astronaut-tended experiment designed to initiate the process of defining researcher flight operations, using an experiment system examining the gene expression changes that occur in model organisms during suborbital spaceflight.

The successful activation of KFTs during the Virgin Galactic Unity 22 mission demonstrated that experiment processes can be operationalized within the context of a crewed suborbital flight. The KFTs were taken on board the spacecraft within a wearable, carry-on pouch by the astronaut and then activated at key points in the flight. This approach balanced science concepts against the demanding safety and operational requirements of what was the first fully crewed flight of the vehicle. Within those operational constraints, and using methods of analysis derived from ground based simulations of suborbital flight profiles^2^, significant changes in gene expression patterns were detected in Arabidopsis at each phase of flight.

In many biological systems, responses in gene expression have been shown to be episodic and wave-like, not linear over the time-course of the stimulus, especially for stimuli related to environmental components that are outside of life evolutionary history, such as spaceflight^1,10,11,19,30^. Moreover, some episodic gene expression patterns change rapidly. For example, plants are able to activate Ca2+ and ROS waves as well as transcriptional alteration within seconds to minutes in response to abiotic stressors^12,31^. The phase-specific profiling of transcriptome changes in Arabidopsis during the Unity 22 flight demonstrates that signaling pathways related to ROS, light, heat and metabolic process, vary during the flight. A large portion of the differentially expressed genes in suborbital flight are ROS related (Fig. 4), primarily those represented in core DEGs of ROS Wheel Cluster III^15^. The ROS Cluster III genes are typical of a rapid-response to high intensity light and are also represented in the rapid oxidative stress response of peroxisome-dependent genes^32^. The predominance of this category of ROS genes suggests that rapid changes in the gravity environment are perceived by plants as an acute stress requiring mitigation through the ROS signal transduction pathway. The engagement of ROS signaling in response to stress is very broadly applied in plants, and includes numerous biotic and abiotic conditions^33,34^. In plants grown in sustained microgravity environments of orbital habitats, ROS-associated genes are also highly represented in the transcriptomic resposnes^8,25,35,36^, but the patterns of ROS gene representation are more evenly distributed among the ROS Wheel Clusters.

The microgravity phase (time point of F3 when the vehicle was ending the parabolic phase of suborbital spaceflight) elicited the most robust change gene expression in both roots and leaves (Fig. 2a, b). This phase represented the first several minutes of access to the spaceflight environment, primarily microgravity. The overrepresented genes included ROS associated LHC genes that were up-regulated, and genes of ROS scavenger GSTs and cytochrome P450s that were down-regulated (Figs. 4 and 6b and Supplementary Table 3). These gene expression changes were consistent with the active ROS adjustment and altered metabolic/biosynthesis processes in plants grown in and fully adapted to orbital spaceflight^5,8,16^, which suggested that the plant sensing of environmental changes in spaceflight and initiation of signaling transduction for long-term adaptation to space occurs within minutes during the start of the journey into space.

The plant suborbital transcriptome also exhibited gene expressions that were unique and distinct compared to orbital gene expression patterns. First, genes not typically observed to be differentially expressed in orbit were altered in expression during suborbital spaceflight. Genes in this category tend to be associated with transient processes. For example, the genes of histone proteins and their modification enzymes were downregulated. These genes encode proteins important to DNA replication in S-phase and associated with cell cycle regulation^37,38^. The repression of these genes in roots and leaves suggest an alteration in DNA replication and cell cycle processes, which might be a component of a transient adjustment at the early stage of space access in plants (Fig. 7). Similarly, genes of the superfamily of bHLH transcription factors, which participate in multiple biological processes in plants^39^, were also repressed in roots and induced in leaves during suborbital flight, but are not typical of a response to sustained orbital habitats.

Second, in addition to a qualitative difference in the types of genes that are differentially expressed between suborbital and orbital environments, there is a quantitative difference in how the same genes and gene families are differentially expressed in both environments. A major example is found with genes encoding heat shock proteins (HSPs) and heat shock factors (HSFs), which are commonly represented in orbital plant spaceflight transcriptomes^9,21,22^. In most stress treatments of Arabidopsis, which includes orbital spaceflight and terrestrial stressors, altered genes from various HSP gene families show a consistent trend of either up- or down- regulation. For an orbital example, the HSP DEGs from the ISS APEX03-2 and APEX04 Col-0 datasets were consistently up-regulated^13,14^. HSP DEGs also exhibit consistent expression patterns within their subgroups in response to terrestrial stress. For instance, in Arabidopsis plants exposed to wounding, heat and ultraviolet-B light, the various subfamilies of HSPs (HSP20, HSP70, HSP90 and HSP100 group genes) all show consistent expression patterns within their groups in the same tissues and conditions^40^. However, in VG Unity 22 DEGs, while HSP genes were largely induced, the differential expression patterns among the subfamilies were not consistent. Most of repressed HSP genes belonged to HSP70 and HSP90 subfamilies, however, some HSP70s were also upregulated (Fig. 7). Greater consistency is seen among the small HSPs (HSP20s) gene expression patterns, almost all of which were induced in suborbital flight. The HSP20 family is not highly represented in the orbital spaceflight transcriptomes of APEX03-2 and APEX04, and their abundance in the suborbital could suggest a unique role for small HSP chaperones in the suborbital spaceflight responses of plants.

Arabidopsis plants in parabolic flight aircraft^41^ also showed responses typical of abiotic stress, including representation of ROS-associated^1,42,43^. This ROS-associated commonality suggests that plant responses to parabolic flight and suborbital spaceflight share some regulatory pathways. Yet, the substantial differences in the plant transcriptomic profiles between parabolic flight and suborbital spaceflight suggest that plants cope with these two distinct environments differently and engage those related pathways through different strategies.

The ability to evaluate the transcriptomic changes that are accumulated during progressive stages of a single suborbital flight enables the quantitative and qualitative evaluation of the early stage adjustment to entering space, including a survey of phase-specific biology between hyper- and micro-gravity in the process of suborbital spaceflight transportation^44^.

Prior to this human-tended experiment, all suborbital spaceflight transcriptomic data were derived from the endpoint, after landing and some dwell-time on the ground. Orbital spaceflight transcriptome data are derived from plants grown in sustained orbital habitats (recently reviewed^9,45^).

This experiment successfully demonstrated the ability to capture transcriptomic changes over the course of a suborbital flight, but it was also a success in the quality insight it revealed in the challenges of conducting such an experiment. For example, managing ground controls was a challenge in that it was difficult to align the time points accurately to those of human-tended flight operations, and it was impossible to re-create the temperature and lateral-motion environments that were experienced by the Flight samples as part of the wearable payload. To help normalize the environmental variables experienced by the Ground Controls, samples from all ground control time points were pooled, and then compared to each Flight time point, rather than attempting to use the individual ground controls collected at each flight time point. This approach was first tested for efficacy during method development studies; one pooled control set derived from three time points sufficiently profiled the transcriptomic responses to clinostat-caused gravity alteration in plants grown in KFT micro-environment^2^. The importance of precise time-matched flight and ground samples could be related to the sensitivity to short-term gravity switch in both roots and leaves of Arabidopsis. With the development of advanced suborbital space transportation, improved experimental workflow will allow more extensive application of fast biological survey accurately in multiple species.

Through this flight, VG also developed lessons learned on creating efficient and effective requirements for human-tended experiments including (1) creating controlled environments for sensitive biological payloads, (2) adapting ground training methods for human-tended researchers and (3) placement of human-tended payloads in the cabin and tethering methods. Regardless, this human-tended suborbital spaceflight experiment demonstrated the operational effectiveness of utilizing simple, spaceflight-vetted hardware to preserve the transcriptomic record of terrestrial biology as it initiates the physiological adaptation to the novel environment of space. The resulting readout suggests that the genetic tools that plants utilize to adjust to the transition to space are distinct from the tools needed for successful long-term physiological adaptation to an orbital, microgravity environment. Elucidating the role of the transcription factors and other rapid-response genes engaged along the various stages of the suborbital flight will further our understanding of how terrestrial organisms set the stage for successful acclimation to microgravity, and other environments outside their evolutionary experience.

## Methods

### Arabidopsis plants and growth conditions

Plant growth conditions applying Kennedy Space Center Fixation Tubes (KFTs) has been previously described^2^. In brief, the *Arabidopsis thaliana* seeds of Col-0 were grown on 10 cm square petri plates on solid nutrient gel under constant light in a broad-spectrum LED bank (100 µmoles/m^2^)^1^. After 4 days plants were hand-carried to the launch location on a commercial air flight (around 8-h dark), and then the plates of seedlings were placed in an LED-lit (80 µmoles/m^2^) growth habitat fashioned in the Virgin Galactic Hanger and grown for three additional days.

At 04:00 on Launch Day 6, 7-day old plants were transferred from the surface of the growth plates to the surface of a previously prepared stainless steel coupon containing a surface layer of nutrient gel (Fig. 1b). The prepared coupons were kept vertical in a ventilated, cool (23–25° C) environment until being assembled into the Flight and the Ground Control KFTs at 07:00. The Flight KFTs were Turned Over to the Virgin Galactic support crew at 07:20, and were then taken by the support crew to the astronaut.

### Operations during the Virgin Galactic suborbital spaceflight

The Virgin Galactic suborbital flight 2018 of VSS Unity VP-03 FLEX payload has been previously described^1^. The human-tended suborbital spaceflight experiment of this study was carried out by Virgin Galactic Unity 22 mission, 11 July 2021. The flight launched at 10:40 am EST. The spaceship, *VSS Unity*, was mated under the wings of the mothership, VMS Eve. After achieving release altitude of ~45,000 ft, *VSS Unity* was released from *VMS Eve* and burned its rocket motor for about 60 s. It was during this phase that astronauts in the cabin were subjected to sustained 3gs—peaking at about 4 g during the horizontal to vertical turn toward space. *VSS Unity* climbed to reach around 86 km altitude, followed by ~3 min of microgravity. Whereafter, the spaceship began its reentry, and descended back to Spaceport America in New Mexico—its original launch point—ultimately landing on the runway it took off from. This profile contained a mixture of short hyper- and hypo-*g* spikes prior to landing, which was 14 min and 17 s after release from Eve. The actuation of KFTs for RNAlater fixation of plant tissues was performed at three time points, including F1 (at about 4 min to release of the spaceship from mothership at ~1 g), F2 (immediately following the hyper-*g* phase of the rocket motor burn of spaceship and the start of microgravity), and F3 (the end of microgravity, which was also the start of re-entry). On the ground, control KFTs were actuated at comparable time points. The timeline for F1, F2 and F3 was communicated to the investigators by radio, although there was a lag in communication that made it impossible to coordinate timing with Flight actuation precisely. One KFT was used for each time point, each KFT held 6 seedlings. In the RNA-seq analyses, samples from three ground KFTs were pooled as the Ground Control as previously described^2^.

The KFTs were deployed as a wearable payload. An experiment-unique pouch was constructed that could hold three KFTs and was attached to the astronaut’s calf for easy-access during the flight (Fig. 1c, insert). The loaded KFTs were delivered to the astronaut just prior to launch preparations, who then secured them within the pouch via tether just prior to being deployed to the vehicle. The KFTs remained secured in the pouch until each designated KFT was retrieved by the astronaut for actuation (which brings the fixative into contact with the biology) at the appropriate time point. Actuated KFTs were returned to the pouch until their return to the investigators post-flight. KFTs of the Ground Controls were kept in an analog pouch that was worn by one of the investigators, and the comparable KFTs were actuated at as close to the flight time point as feasible.

### RNA extraction and library preparation

RNA preparation and sequencing were conducted as previously reported^2^. At least two individual plants from one KFT in each condition constituted a biological replicate and 3 replicates were generated for each condition. Seedlings were dissected into roots, leaves and hypocotyls for downstream applications, whereas the intervening hypocotyls were set aside. RNA extraction for tissues of roots or leaves was performed using Qiagen RNeasy Plant mini kit (Qiagen, Germantown, USA). The concentration of resulted RNA samples was determined on Qubit 2.0 Fluorometer (Thermo Fisher Scientific, Waltham, USA) and the quality was assessed using Agilent 2100 Bioanalyzer (Agilent, Santa Clara, USA). Approximately 100 ng of total RNA was used to produce mRNA using NEBNext Poly(A) mRNA Magnetic Isolation Module (NEB, Ipswich, USA). RNA library construction was performed using NEBNext Ultra II Directional RNA Library Prep Kit (NEB) with 2/3 of poly A enriched RNA. Each uniquely barcoded library was enriched by 15 cycles of amplification, and then purified with AMPure beads (Beckman Coulter, Indianapolis, USA). The prepared libraries were sized on the Bioanalyzer and quantified using the Qubit 2.0 Fluorometer.

### RNA sequencing

The individual libraries were pooled in equimolar concentration, then treated with Free Adapter Blocking Reagent (Illumina, San Diego, USA) in order to minimize the presence of adapter-dimers and index hopping rates. This library pool was diluted to 0.8 nM and sequenced on Illumina NovaSeq6000 platform using one S4 flow cell lane (2 × 150 cycles). The instrument computer was using NovaSeq Control Software v1.6 while Cluster and SBS consumables were v1.5. The final library loading concentration was 120 pM with 1% PhiX spike-in as a control. The fastq files were generated using the BCL2fastQ function in Illumina BaseSpace portal.

### RNA-seq analysis pipelines

Reads acquired from RNA-seq were cleaned up using the cutadapt program^46^ to trim off sequencing adapters and low-quality bases with a quality phred-like score <20, and then reads <40 bases were also excluded. The *Arabidopsis thaliana* genome (version TAIR10.51) was used as the reference sequences for RNA-seq analysis. The mapping of cleaned reads was done using STAR package (Spliced Transcripts Alignment to a Reference, v2.7.9a)^47^, and mapping results were processed with the HTSeq (High-Throughput Sequence Analysis in Python, v0.11.2)^48^ samtools as well as scripts developed in house at ICBR of UF to remove potential PCR duplicates and count uniquely mapped reads for gene expression analysis. For differential expression analysis, flight samples of three time points had 3 replicates for each, while samples of three ground time points were considered as 9 replicates of one Ground Control. The counted reads of each gene were analyzed by a DESeq2-based R pipeline. Genes were removed from differential analysis if Fragments Per Kilobase of transcript per Million fragments mapped (FPKM) ≤ 3 in all conditions after combining biological replicates. Differentially expressed genes (DEGs) between samples of three flight time points and Ground Control were selected using the threshold of Log_2_(fold-change) >1 or <−1, and FDR adjusted *p* value (Padj) < 0.05. Enrichment analysis for process and pathway was conducted using Metascape (https://metascape.org/gp/index.html#/main/step1)^49^, and that for gene family was performed using GenFam (https://www.mandadilab.com/genfam/)^50^.

### Generation of t-SNE plot and clustering analysis

Generation of t-SNE plot and clustering were performed using R (4.2.1). A CSV file with the expression data of genes that were differentially expressed in at least one condition were used to generate the t-SNE plot, with non-significant values replaced with zero. The t-SNE package, Rtsne^51^, was used with default parameters aside from the eta parameter altered to a value of 2000. Clustering was conducted using DBSCAN with the fpc package^52^ with default parameters aside from an eps of 2, and MinPts of 10. The clusters identified by DBSCAN were then colored and projected using the plotly chart studio web tool (https://plotly.com/).

### Reporting summary

Further information on research design is available in the Nature Research Reporting Summary linked to this article.

### Supplementary information

Supplementary Tables

Reporting Summary

Supplementary Video 1

## Data Availability

RNA-seq data have been deposited with the accession number of PRJNA984625 in BioProject (https://www.ncbi.nlm.nih.gov/bioproject/) of National Center for Biotechnology Information (NCBI) and with the OSD identifier of OSD-624 (10.26030/8ane-wq52) in NASA GeneLab (https://genelab.nasa.gov/).

## Code availability

All codes used in this work have been described in “Methods” section. No previously unpublished codes were used.
