# Supplementary material for: Transcriptomic dynamics in the transition from ground to space are revealed by Virgin Galactic human-tended suborbital spaceflight
Source: NPJ Microgravity. 2023 Dec 20;9:95. doi: 10.1038/s41526-023-00340-w (PMC10733374; doi:10.1038/s41526-023-00340-w)
Supplement: Supplementary file 1 — Supplementary Tables [file 41526_2023_340_MOESM1_ESM.pdf]

Transcriptomic dynamics in the transition from ground to space are revealed by Virgin Galactic human-tended suborbital spaceflight

Robert J. Ferl[1,4,#,*], Mingqi Zhou[1,#], Hunter F. Strickland[1,2], Natasha J. Haveman[1], Jordan B. Callaham[1], Sirisha Bandla[5], Daniel Ambriz[5], Anna-Lisa Paul[1,3,*]

[1]Department of Horticultural Sciences, University of Florida, 2550 Hull Road, Fifield Hall, Gainesville FL, 32611, USA
[2]Plant Molecular and Cellular Biology Program, University of Florida, 2550 Hull Road, Fifield Hall, Gainesville FL, 32611, USA
[3]Interdisciplinary Center for Biotechnology Research, University of Florida, 2033 Mowry Road, Gainesville FL, 32610, USA
[4]UFResearch, University of Florida, 1523 Union Rd, Grinter Hall, Gainesville, FL, 32611, USA
[5] Virgin Galactic, 1700 Flight Way, 3rd Floor, Tustin, CA, 92782

[#]These two authors share first authorship.
*Correspondance: Robert J. Ferl (robferl@ufl.edu) and Anna-Lisa Paul (alp@ufl.edu)

Supplementary Table Information
(table legends are also included at the top of each Table)

Supplementary Table 1. DEGs between Flight and Ground Control in Arabidopsis roots from human-tended and non-human-tended VG suborbital spaceflight experiments. Log^2FC numbers highlighted in yellow indicate significant changes (Padj < 0.05). Red indicates upregulation ($Log^2FC > 1$) and blue indicates downregulation ($Log^2FC < -1$).

Supplementary Table 2. Annotation and t-SNE clustering of DEGs between Flight and Ground Control in Arabidopsis roots or leaves from human-tended VG suborbital spaceflight experiments. Log^2FC numbers highlighted in yellow indicate significant changes (Padj < 0.05). Red indicates upregulation ($Log^2FC > 1$) and blue indicates downregulation ($Log^2FC < -1$). DEGs belonging to each functional category are highlighted by purple bars, and those with significant changes in APEX03/APEX04 spaceflight are highlighted by maroon bars.

Supplementary Table 3. Gene families that are significantly enriched in each t-SNE cluster of DEGs between Flight and Ground Control in Arabidopsis roots or leaves from human-tended VG suborbital spaceflight. Threshold of significant enrichment is FDR Padj < 0.01.

Supplementary Video 1. Exhibition of 7-day old Arabidopsis seedlings loaded on the coupon inserted in the KFT. This setup is prepared for human-tended suborbital spaceflight. Author Robert Ferl’s hand is visible in the video, and he consents to the use.

Supplementary Table 1. DEGs between flight and ground control in *Arabidopsis* roots from human-tended and non-human-tended VG suborbital spaceflight experiments. Log2FC numbers highlighted in yellow indicate significant changes (Padj < 0.05). Red indicates upregulation (Log2FC > 1) and blue indicates downregulation (Log2FC < -1).

DEG	F1R vs GR	F2R vs GR	F3R vs GR	Non-human tended
AT5G12020	4.216718	5.042931	4.28459	0
AT3G07365	4.165475	4.255888	4.494545	0
AT1G53540	4.06149	4.917348	4.324765	0
AT3G46230	3.900891	4.595676	3.832939	1.566132384
AT5G59310	3.633171	3.191085	3.484839	0
AT5G12030	3.523844	4.419821	3.622538	0.802777087
AT4G25200	3.288786	4.25889	2.660264	0
AT2G29500	3.093928	3.587851	2.839218	0.315672641
AT4G27670	3.044989	4.486064	3.578685	0
AT1G72660	2.930665	3.195148	2.893988	0
AT3G09640	2.777945	3.335524	3.223334	0
AT4G10250	2.743394	3.932565	3.207527	0
AT1G07400	2.729125	3.078603	2.152146	2.283558601
AT5G48570	2.629963	2.487407	2.283606	1.297654224
AT4G21320	2.38917	2.601264	2.765459	0.260451878
AT5G02810	2.368592	1.54	3.121996	1.262025741
AT2G36690	2.267007	1.329327	1.794874	-0.629079828
AT1G16030	2.011317	2.982081	2.598904	0.919231521
AT3G53830	2.006512	1.421122	2.267396	-1.568948969
AT3G21520	1.812323	2.201173	1.847756	-1.157552289
AT3G14620	1.812057	1.729229	1.933181	0.488676056
AT1G52560	1.759724	2.571227	1.986799	0
AT2G45220	1.753503	1.359277	1.491997	0.278067968
AT2G47180	1.714637	1.577774	1.826593	-0.228787911
AT5G64170	1.636696	1.518106	1.760263	0.642934211
AT2G21130	1.532125	1.97956	1.031382	-0.244830446
AT5G57345	1.529001	1.395954	1.453336	-0.153012424
AT3G24100	1.522008	1.611207	1.303972	0.235473372
AT5G12110	1.408043	1.770589	1.480163	-1.097021064
AT2G43050	1.384906	1.955138	2.03113	0.016541893
AT1G09140	1.348256	1.267761	1.336809	0.181662211
AT1G53560	1.336472	1.303434	1.128949	0.091309779
AT1G05340	1.307938	1.392754	1.024712	0.25916227
AT5G59720	1.247581	2.349741	2.443903	-0.255460975
AT2G17900	1.247011	1.153895	1.39694	-0.804897064
AT1G17870	1.234852	1.246557	1.577568	1.434941679
AT4G12400	1.178512	1.037801	1.105737	0.230913631
AT1G22770	1.155735	1.177153	1.316926	0.260932321
AT2G21180	1.152515	1.370282	1.129413	-0.029482874
AT5G47830	1.0451	1.492457	1.001849	-0.550004898
AT3G47500	1.00311	1.08642	1.431843	-0.565046732
AT3G30775	-1.115234	-1.125539	-1.170141	-0.081808635
AT3G51240	-1.118587	-1.269922	-1.735583	0.584661413
AT5G41080	-1.27182	-1.026615	-1.881202	0.100807341
AT3G09440	-1.283624	-1.21149	-2.130376	0.218173278
AT3G03780	-1.334834	-1.138053	-1.358276	-0.005569445
AT2G19800	-1.416281	-1.782491	-1.184144	-0.075828498
AT5G05410	-1.465562	-1.60729	-2.193693	0.329682788
AT2G01150	-1.499881	-1.158495	-1.534765	0.169014884

AT4G11660	-1.580804	-1.720317	-2.119695	0.737408641
AT5G48070	-1.626477	-1.354151	-2.265612	0.732696073
AT1G77530	-1.920377	-2.037929	-1.729753	-0.914048925
AT1G72416	-2.143392	-2.27646	-2.490559	-0.356659299
AT3G14200	-2.210698	-2.066235	-2.485751	0.020667517
AT1G77520	-2.309494	-1.554439	-2.008605	-0.836011128
AT1G14200	-2.315524	-2.266047	-3.297081	-0.10731455
AT3G01260	-2.5041	-2.275703	-4.018825	-0.144584627
AT3G63350	-2.925821	-1.867086	-2.792691	-0.961429698
AT3G51910	-2.931173	-2.931642	-2.99228	-0.104628154
AT5G66780	1.631016	1.449417	-0.129591	1.363566212
AT3G02535	1.539091	1.743924	0.77836	2.052526396
AT1G56300	1.485821	1.402647	0.850122	0.203715899
AT5G67488	1.448369	1.721648	-0.233614	0.460993219
AT5G56985	1.439937	1.36708	0.411495	0.509605373
AT5G09978	1.358983	1.129875	0.089971	0.200396876
AT4G27310	1.352594	1.356563	0.844588	0
AT4G23680	1.31762	1.130266	0.940622	-0.089378139
AT2G36210	1.30492	1.067659	0.784842	1.422736983
AT3G10020	1.287356	1.270572	0.99254	-0.028061334
AT5G23950	1.260541	1.122348	0.245233	0.097127648
AT1G75750	1.238048	1.147584	0.94673	0.059327277
AT4G04125	1.130472	1.638557	0.023596	0.097088841
AT5G01815	1.125838	1.900914	0.285972	-1.823163792
AT3G48020	1.074765	1.463699	0.589108	0.19589988
AT5G64230	1.039307	1.511403	0.912386	-1.626017632
AT1G80240	1.024525	1.008564	-0.187396	0.245818168
AT2G39380	1.006684	1.038041	0.879923	-0.058708762
AT4G38470	-1.004229	-1.300692	-0.527516	-0.365550729
AT3G43790	-1.062923	-1.029714	-0.052412	-1.021141096
AT1G71880	-1.131434	-1.196134	-0.405212	-0.072699201
AT4G18340	-1.265081	-1.093341	-0.368571	-0.290692618
AT1G77410	1.737003	1.205985	1.693103	-0.524865866
AT3G47470	1.561164	1.308576	2.233887	0.359377847
AT1G73750	1.406917	0.309631	1.234275	0.905659337
AT3G15400	1.248032	0.975575	1.3319	-0.081680711
AT2G30480	1.18775	0.612213	1.711739	-0.541971843
AT3G16770	1.152038	0.778405	1.926131	0.040155714
AT5G47560	1.120182	0.908814	1.481345	-0.196841276
AT2G24545	1.108775	0.847323	1.094394	-0.804635334
AT5G01600	1.080546	0.69049	1.425451	0.293455816
AT5G37340	1.064659	0.870767	1.057341	-0.59185056
AT1G70890	1.061792	0.525177	1.149483	0.210982971
AT2G37180	1.051639	0.883943	1.638514	0.270395436
AT2G46830	1.033461	0.191219	1.720909	0.036489777
AT1G44446	1.020162	0.805074	1.828591	-0.356807716
AT5G62020	-1.01893	-0.914067	-1.404083	0.277366942
AT3G16050	-1.067697	-0.919366	-1.577891	-0.03246082
AT1G66080	-1.18022	-0.865919	-1.852268	0.584904377
AT5G23020	-1.198342	-0.857997	-1.681344	0.047789765
AT5G59820	-1.205176	-0.501604	-1.401091	-0.198020238
AT5G52020	-1.274136	-0.372132	-1.612361	1.165402986
AT3G57520	-1.277967	-0.881966	-1.06685	-0.315078723
AT2G03090	-1.284783	-0.797696	-1.205445	0.331374071
AT4G16370	-1.414453	-0.469594	-1.594989	-0.291721153

AT1G27140	-1.421021	-0.384965	-2.174562	0.089650619
AT3G58990	-1.624697	-0.772085	-1.805892	0.164443534
AT1G05700	-1.733813	-0.411823	-1.54292	0.172859379
AT5G59680	-2.172715	-0.800144	-1.857364	-0.149316383
AT2G32487	-2.872857	-1.269447	-2.334702	-0.556089355
AT4G20970	-2.984233	-1.840313	-4.20842	0
AT1G26240	-3.579884	-1.120445	-3.753697	-0.520567943
AT2G27390	7.179772	-1.929317	-1.929317	0
AT3G02480	3.013046	1.094285	0.039073	0.531920173
AT5G65080	2.539585	0.901328	0.524842	0.345099279
AT1G02205	2.176505	0.350418	-0.473127	-0.46152531
AT2G23110	2.173891	1.704273	1.652632	0
AT2G21820	1.941384	1.527626	0.957123	0.553894854
AT2G31980	1.62	0.720406	0.632442	0.831450011
AT5G67140	1.598734	1.153967	0.907651	0.520128277
AT5G45810	1.567945	0.1981	-0.087114	0.05585537
AT3G44326	1.477819	1.169825	0.789794	0.197711005
AT4G30470	1.432662	0.572562	-0.052246	0.612785939
AT4G40010	1.386977	-0.003235	0.395484	0.473505119
AT5G43570	1.337961	0.560316	0.662693	-0.378268366
AT2G01530	1.307637	0.419159	0.409763	-0.172173356
AT1G02400	1.305033	0.569534	0.481325	1.013820779
AT4G40070	1.252833	0.940369	0.472159	0.103460776
AT5G54300	1.244075	0.552751	0.878944	-1.282372196
AT4G32590	1.236103	0.958521	0.846138	-0.230989946
AT1G62370	1.225279	0.992608	0.297362	0.285174309
AT1G64610	1.109557	0.359427	0.869727	0.117554977
AT2G40230	1.105413	0.982527	0.637824	0.185269718
AT1G20440	1.093579	0.799255	0.494424	0.233936694
AT3G26450	1.086117	0.559806	0.887745	-0.459470616
AT5G24930	1.077466	0.797426	0.743844	-0.170781008
AT4G09760	1.059695	0.429678	0.865787	0.425422175
AT5G10300	1.050549	0.979961	0.971468	-0.320729896
AT1G07150	1.024278	0.613588	0.427876	0.290540874
AT2G44670	-1.012964	-0.429062	-0.856808	0.539218232
AT1G49870	-1.040771	-0.831065	-0.727987	-0.177684695
AT4G02800	-1.066081	-0.71784	-0.594039	-0.451709481
AT3G13310	-1.089788	-0.290695	-0.628915	-0.136579551
AT5G08350	-1.138312	-0.488092	-0.561931	0.373235004
AT1G22885	-1.184697	-0.749212	-0.682139	-0.296799283
AT3G20470	-1.26923	0.131559	-0.40241	-0.058077152
AT5G47240	-1.285494	-0.77495	-0.716291	-0.903576389
AT1G75000	-1.563527	0.408226	-1.030914	-0.427705729
AT1G11330	-1.625244	-1.000497	-0.311479	-0.319431966
AT4G30110	-1.910509	-0.976765	-1.011486	-0.547068941
AT5G06730	-2.12991	-0.523685	-0.411957	1.302650291
AT4G16240	-3.731688	-0.634149	-1.401884	0
AT1G70830	2.926244	3.145675	4.586487	-0.985889056
AT4G33720	-0.481336	4.098788	2.813569	-0.966263747
AT5G07330	1.406978	2.305217	1.964978	0.472183259
AT5G06980	1.135535	1.376926	1.940125	-0.048439702
AT5G67430	1.660326	1.89383	1.891703	1.430635744
AT1G47655	0.611115	1.123764	1.690156	0.128470311
AT1G28660	1.062224	1.633298	1.511136	-0.217686081
AT2G26690	0.879443	1.01373	1.472436	0.065627183

AT1G03070	1.140244	1.845265	1.438955	0
AT2G32120	0.998669	1.317333	1.352457	-0.015685432
AT2G46790	1.002819	1.492866	1.34123	-1.943113874
AT1G05347	0.820065	1.668425	1.326686	0
AT5G56600	1.005675	1.705249	1.320285	0.627549625
AT5G01880	1.01396	1.102173	1.294419	0.112171118
AT2G21320	0.596295	1.398744	1.277043	0.840375539
AT3G12320	1.015459	1.381827	1.271423	0.000595947
AT5G39090	1.155748	1.396939	1.221701	0.781097639
AT3G06470	0.437195	1.043594	1.052182	0.180129796
AT2G01010	0.606197	1.901027	-1.162058	0.051990077
AT3G27030	0.045082	1.389664	-1.171363	0.112936363
AT1G64220	-0.037902	1.348992	-1.192297	0.341508473
AT2G01020	-0.531923	3.352589	-2.474923	0.214170618
AT3G41979	-0.531923	3.352589	-2.474923	0.214170622
AT2G08770	-0.608317	2.508266	-2.986555	0.951988168
AT3G22840	-1.077401	-1.064502	-2.166505	1.318632886
AT5G10230	-0.419895	-1.080493	-1.826657	-0.425265665
AT5G10625	-0.974157	-1.082044	-1.167373	0.178563773
AT1G64390	-1.267608	-1.103235	-1.287977	0.07845008
AT1G21120	-0.819428	-1.110484	-2.509646	0.057888492
AT5G08640	-0.962638	-1.124625	-1.571627	0.644380395
AT5G49350	-0.467084	-1.126502	-1.099112	-0.573789433
AT5G40780	-0.608488	-1.158153	-1.085134	-0.139251958
AT1G29270	-0.992828	-1.173217	-1.295722	-0.02130591
AT3G15340	-0.742525	-1.220985	-1.154356	0.091714967
AT2G34790	-1.14687	-1.274233	-1.631086	-0.002614267
AT1G49430	-0.04126	-1.303809	-1.259144	0.261019551
AT2G03830	-0.902176	-1.310044	-1.38112	-0.328645119
AT3G44550	-0.486889	-1.339568	-1.349594	-0.552321042
AT5G62430	-0.978216	-1.408231	-2.193501	0.583290448
AT3G04370	-0.260492	-1.432362	-1.472871	0.361004127
AT2G26150	-1.138655	-1.494756	-1.382194	0
AT1G67110	-1.15208	-1.503497	-1.331369	-0.256569664
AT5G49620	-1.314999	-1.556885	-1.664959	-0.51548649
AT1G59730	-0.603281	-1.639691	-2.528951	-0.48908799
AT1G55940	-0.718611	-1.63999	-1.156154	-0.408632858
AT4G35160	-0.84809	-1.697794	-1.515289	-0.38170719
AT5G62360	-1.369192	-1.819534	-2.466561	0
AT1G30760	-1.859478	-1.821661	-2.71806	0.141400989
AT5G12270	-1.421199	-1.936775	-2.143151	-0.511612727
AT1G01580	-1.355545	-2.285458	-3.259857	0.42279607
AT4G21400	-0.726941	-2.628019	-1.035961	-0.303912621
AT4G19690	-2.24278	-2.796674	-3.460338	0.514000757
AT5G19890	-1.861776	-3.070502	-2.898552	-0.324582311
AT1G64160	-2.159583	-3.552051	-5.52994	0.046522746
AT2G15780	-2.024449	-3.919219	-5.570754	0
AT5G59220	1.744524	1.930995	1.166112	1.412823441
AT1G06002	-0.905895	-12.31423	-0.889454	11.08945758
AT5G10435	10.24649	10.59382		0
AT1G15405	-0.923263	4.14679	-1.364609	1.099471608
AT2G03230	0.767375	3.986566	-0.425822	0
AT5G04305	-0.721724	3.880508	-5.178514	0
AT2G09465	-0.105541	3.413528	-2.323762	0
AT5G01515	-0.066876	3.33913	-3.516786	0

AT3G01715	0.774588	3.103484	-1.892221	0.505656895
AT5G09125	0.122846	2.625397	-1.36985	0
AT5G09585	-0.198323	2.571355	-1.355098	-0.454861024
AT3G12510	0.838065	2.487858	0.744825	0
AT2G08760	-0.433353	2.470426	-1.883001	0.530859174
AT1G56600	1.915624	2.338281	0.638761	0
AT2G06835	-1.582177	2.299842	-1.177739	1.118383338
AT1G09950	0.344134	2.297899	0.446413	0.977915411
AT5G53902	0.727159	2.225174	0.573563	0.042354933
AT2G41342	0.459097	2.225118	1.193747	-0.661999649
AT1G07803	1.448604	2.108005	0.480025	0.625719762
AT1G07430	1.560594	2.087194	1.251464	0
AT5G09065	0.580254	2.043265	-0.216067	0
AT5G06845	0.362119	2.027688	-0.093714	0.438991038
AT3G48510	1.471205	1.924841	0.13775	0.943449642
AT3G57765	0.520666	1.921867	-0.473678	0
AT4G39404	1.331221	1.900367	0.25251	-0.937447782
AT4G06795	0.968176	1.887055	-0.316685	0
AT1G09363	0.70495	1.849854	0.340181	2.068599922
AT3G03270	0.300857	1.835518	0.589587	0.20908385
AT5G03285	1.001971	1.830839	0.768855	0
AT3G56825	-0.03959	1.807221	-0.729327	0.902233175
AT4G29610	0.006979	1.791484	-0.438293	2.337141418
AT5G23230	1.287051	1.759603	1.347366	-0.908143703
AT5G54075	-0.189889	1.720665	-0.696652	1.781382778
AT2G18193	0.97904	1.696662	0.548311	0.439349665
AT4G00165	0.783474	1.657854	0.762443	0
AT1G47600	0.627294	1.649757	-1.338493	-0.067324951
AT1G34047	0.362757	1.64268	1.36374	-0.550420901
AT5G47450	1.425326	1.608182	1.036748	0.271350589
AT4G39235	0.969104	1.600696	0.508469	0.170811081
AT3G41762	0.349342	1.574142	-0.55885	0.549383051
AT4G33660	0.799315	1.572224	1.070383	0
AT5G65990	1.136658	1.543503	0.767089	1.50900448
AT4G36010	0.681214	1.521338	0.725516	0.582649391
AT2G01008	0.110501	1.504435	-0.368115	0.641496485
AT5G58070	0.818761	1.497766	0.676897	0.19750352
AT4G12495	-0.18717	1.491834	-0.6454	0
AT3G08590	0.475012	1.485825	0.254638	-0.227205295
AT1G11700	0.706864	1.462969	0.626363	-0.70883833
AT5G27660	0.312869	1.438113	-0.69432	0.296846916
AT5G56540	0.544564	1.435145	0.039318	0.083327856
AT5G59320	0.409965	1.395177	0.375707	0.849744784
AT2G19310	0.891837	1.395094	0.61248	-0.044849028
AT2G28710	0.926296	1.390007	0.818468	0.09344256
AT1G01940	0.920069	1.388418	0.906361	-0.145711459
AT1G09800	-0.08367	1.387957	-0.261544	-0.637206182
AT1G14980	0.724117	1.382638	0.003283	0.076668125
AT1G16825	0.329855	1.382135	0.187001	1.614267924
AT5G50175	0.988511	1.37983	0.088906	-0.312810052
AT1G24600	1.076222	1.370651	-0.287181	0.507198146
AT5G10990	0.717107	1.363935	0.08297	-0.190995167
AT3G62990	0.307781	1.357682	0.182141	2.031560652
AT3G28740	0.784507	1.35373	0.603865	0.20408461
AT1G07600	1.289187	1.352235	-0.860972	-0.054855973

AT1G16820	0.553537	1.343169	0.212605	0.834272086
AT4G12490	0.165431	1.30484	-0.807561	-7.939231414
AT2G33740	0.850502	1.304509	0.75498	-0.187262191
AT5G01550	1.067895	1.297288	0.651409	0.137984313
AT3G06355	0.086511	1.284085	-0.498719	0.106416155
AT2G47730	0.741501	1.281939	0.578034	0.298255784
AT5G50760	0.828774	1.279686	0.157529	0.136537045
AT1G22985	0.933955	1.278524	0.678113	-0.363366609
AT1G03660	0.411611	1.273573	-0.185178	0.167182259
AT4G33666	0.063405	1.262166	-0.022588	-1.21158622
AT3G50350	0.634776	1.226123	0.5062	-0.22464802
AT3G59370	0.825981	1.224476	-0.043206	0.331175469
AT1G72240	0.72235	1.213268	0.10846	0.837430375
AT3G22540	0.756047	1.213177	0.074609	1.575475252
AT5G66590	0.254734	1.210419	-0.432749	0.206749307
AT1G66230	0.78276	1.208778	0.792765	0.262712311
AT5G64180	0.669016	1.199857	0.718081	0.281519747
AT1G05560	0.238051	1.171229	0.896028	-0.304795694
AT1G55990	1.089473	1.165946	-0.372454	-0.12055886
AT5G11975	0.71141	1.16534	-0.175039	-0.311810673
AT1G67810	0.320764	1.165139	0.42905	0.004739043
AT1G65520	0.607739	1.161788	0.252205	-0.041789521
AT1G48750	0.148833	1.141882	-0.175391	0.063662678
AT2G36830	0.886308	1.14007	0.625712	0.07689637
AT1G20190	0.241424	1.138888	-0.09562	-0.032701091
AT5G17350	0.843383	1.129437	0.197906	1.685924422
AT3G50850	0.6701	1.129257	0.131348	0.351566235
AT1G65980	0.764917	1.129079	0.739723	-0.026361014
AT5G03030	0.414657	1.126514	0.201071	0.005391408
AT1G03220	0.819744	1.123235	0.699965	-0.076012826
AT1G73480	0.902908	1.120055	0.416807	0.221087902
AT5G02220	0.559099	1.116044	-0.22535	-0.073608973
AT5G15770	-0.268062	1.115895	-0.370455	0.206193834
AT5G62340	0.473796	1.115646	0.036978	0.116554728
AT5G23460	0.019448	1.112812	0.680506	-0.025336935
AT2G07671	0.151973	1.109714	-0.627221	-0.120384442
AT2G35910	0.293863	1.108104	0.257294	0.188388902
AT3G41768	0.052707	1.10444	-0.728767	0.024019763
AT2G37750	0.882413	1.101242	0.200653	-0.234039778
AT5G45095	0.403955	1.098268	0.884378	0.913129733
AT2G30550	0.864258	1.093334	0.363167	0.396014807
AT1G04980	0.925211	1.093018	0.24278	0.152591132
AT4G33550	0.245256	1.087422	-0.185627	0.089287022
AT4G39360	1.009545	1.08739	0.349825	0
AT4G02980	0.860804	1.084307	0.87728	-0.122141352
AT4G20000	0.964115	1.080739	0.256572	0.510588562
AT3G08690	0.785959	1.079456	0.757991	0.309711829
AT3G01430	0.765033	1.075171	0.489101	0.431561402
AT1G07130	0.534737	1.064705	0.112806	-0.110490468
AT2G41380	0.77266	1.061623	0.076869	0.072350923
AT5G66985	-0.182472	1.060896	-0.176783	0.077058052
AT2G01520	0.906298	1.050027	0.691143	-0.421699451
AT5G16060	0.274005	1.048663	-0.18444	0.193915318
AT5G22890	0.660868	1.047951	0.557211	-0.285358194
AT5G06330	0.788618	1.046303	0.715793	-0.375815954

AT4G00170	-0.032793	1.04314	-0.325116	-0.006363579
AT5G03545	0.294088	1.042705	0.518734	0.55400998
AT3G47836	0.180967	1.04185	-0.282293	0.729572207
AT5G15802	0.467323	1.034681	0.213425	-0.316588109
AT3G25882	0.638434	1.031354	-0.663045	0.478832251
AT4G37320	0.691785	1.030934	0.493336	-0.19882625
AT4G27030	0.772062	1.029462	0.597897	0.689406984
AT2G27660	0.913939	1.028666	0.37288	-0.067070292
AT5G01250	0.652053	1.027931	0.499579	0.020655827
AT4G09695	0.696088	1.027859	0.515416	-0.084957634
AT1G05300	0.742817	1.022597	0.294903	-1.60443308
AT5G23220	0.869789	1.015471	0.086783	0.72832203
AT1G05210	-0.062835	1.014899	-0.503245	-0.724629101
AT3G16240	0.576959	1.014837	0.482485	0.195696634
AT5G08330	0.338168	1.013128	0.355026	0.006083447
AT1G77120	0.162385	1.011651	0.3985	0.333106903
AT5G18790	0.135282	1.005642	-0.044576	-0.463008562
AT2G40150	-0.202737	-1.007905	0.216069	0.496639037
AT3G15220	-0.284781	-1.0106	0.206491	0.136717844
AT2G01130	-0.546206	-1.01066	-0.029679	-0.057785935
AT3G15550	-0.748536	-1.01619	-0.320404	0.625109977
AT2G20440	-0.209667	-1.021602	-0.080605	0.321309509
AT4G15563	0.323062	-1.027665	0.052578	0.171289616
AT3G51740	-0.728314	-1.028825	0.148836	-0.646103098
AT2G32440	-0.47005	-1.031434	-0.150829	0.042590346
AT3G51480	-0.286605	-1.040306	0.117816	0.46047187
AT3G52340	-0.771464	-1.041043	-0.694577	-0.007334122
AT4G16957	0.03566	-1.042398	0.218765	0.372712905
AT3G25820	-0.254729	-1.045342	-0.332353	-0.112718743
AT3G13730	-0.916598	-1.060679	-0.6477	-0.078374528
AT4G00520	-0.27547	-1.064694	-0.018558	0.139132292
AT2G29060	-0.400228	-1.068322	0.045362	-0.377982156
AT1G30820	-0.862519	-1.073627	-0.672622	-0.175593917
AT3G11260	-1.186642	-1.077942	-1.030661	-0.592001235
AT1G10570	-0.419474	-1.078871	-0.10287	0.155683345
AT2G23030	0.06804	-1.081557	0.151201	-0.652712271
AT1G50240	-0.857428	-1.081843	-0.254637	-0.166744996
AT2G46240	-1.040706	-1.09371	-0.896476	0.308243278
AT4G33260	-0.457726	-1.095453	0.374733	0.118218664
AT2G18000	-0.199556	-1.103965	-0.491555	-0.400643397
AT2G18360	-0.490257	-1.107671	-0.328579	0.184058539
AT5G48310	-0.909401	-1.109828	-0.304806	0.56076568
AT1G18270	-0.466026	-1.112161	-0.280043	0.016085204
AT3G44670	-0.356491	-1.115511	0.584683	0.073702751
AT2G37280	-0.92564	-1.115964	-0.231887	-0.62521383
AT5G40840	-0.199177	-1.121698	-0.363038	0.121458505
AT3G03300	-0.154423	-1.124883	0.455795	0.035786652
AT1G21650	-0.394534	-1.127324	-0.356205	1.131900668
AT2G33770	-0.677867	-1.130229	0.202894	0.200108385
AT5G63160	-0.569876	-1.146043	-0.343427	-0.831132425
AT1G48360	-0.20213	-1.148494	-0.176184	0.0141849
AT1G77380	-0.920911	-1.14859	-0.960945	-0.715460657
AT3G48360	-0.17487	-1.153122	0.201555	-0.299239567
AT1G34670	-0.104032	-1.153528	-0.889645	-0.26333123
AT1G49960	-0.182489	-1.167948	-0.954093	-0.907343904

AT3G18400	-0.280125	-1.168752	-0.694978	-0.117355023
AT5G65460	-0.501784	-1.171915	0.014522	-0.326820793
AT2G32250	-0.326226	-1.199316	0.101556	0.115647302
AT1G26260	-0.633972	-1.220824	-0.825437	0.009008542
AT4G36980	0.078568	-1.225905	0.56246	-0.047422373
AT2G34930	-0.851814	-1.226569	-0.398618	0.425775654
AT5G18065	-0.419831	-1.229888	-0.102	0.590546943
AT3G54880	-0.441648	-1.248617	-0.881337	-0.077914439
AT5G20250	-0.883445	-1.256029	-0.3262	-0.631087412
AT5G41370	-0.42932	-1.260681	0.126183	0.298503076
AT1G30410	-0.234278	-1.265283	0.076895	-0.019353611
AT2G22920	-0.936293	-1.266737	-0.238245	-0.228855261
AT2G28470	-0.228017	-1.283253	-0.988068	0.194386843
AT1G05830	-0.303598	-1.283594	0.094102	0.052771385
AT3G16260	-0.236965	-1.288356	0.340712	-0.280916754
AT1G25098	-0.536434	-1.291073	0.474733	-0.006606139
AT1G27370	-0.251357	-1.298193	0.317681	-0.454195763
AT4G37610	-0.600785	-1.308328	-0.391654	-0.053537668
AT5G60430	0.110527	-1.313363	0.253112	0.167132448
AT3G57470	-0.388158	-1.347753	0.464489	-0.119821966
AT4G20320	-0.690302	-1.348861	-0.547721	0.034063747
AT2G30600	-0.777868	-1.350015	-0.291541	-0.15720285
AT5G37690	-0.37529	-1.357508	-1.012599	-1.172579512
AT4G33770	-0.862151	-1.363503	-0.86828	0.345320907
AT5G48220	-0.024056	-1.370076	0.274021	0.165047435
AT4G36670	-0.229499	-1.3814	-0.115507	0.096433471
AT3G49070	-0.792031	-1.402873	-0.963182	-0.063645443
AT5G62630	-1.055277	-1.405176	-0.634601	-0.396487009
AT1G15040	-0.930924	-1.422048	-0.941633	-0.357244641
AT5G44750	-0.210504	-1.43223	0.10128	-0.209391327
AT4G04740	-0.199917	-1.43672	0.228833	-0.182807803
AT1G30420	-0.651402	-1.45501	-0.782767	0.041064066
AT1G19397	0.387416	-1.46778	0.684822	0.19292508
AT2G16750	0.144862	-1.503602	-0.294435	-0.045447845
AT1G65370	-0.518288	-1.529337	0.213945	-0.205311112
AT4G15530	-0.615212	-1.552979	-0.049266	0.529245123
AT1G13670	-1.108312	-1.553236	-1.230053	0.128274048
AT1G54400	-1.049196	-1.618958	-1.113977	0
AT4G03205	-0.329378	-1.629766	0.859419	0.345739807
AT2G28570	-0.253478	-1.640996	0.04213	-0.15987582
AT1G73300	0.11484	-2.229979	0.030514	-0.129639394
AT4G24415	-1.235462	-2.246204	-1.379517	0.535265917
AT1G51270	-0.634106	-2.408212	-0.811254	0.182789855
AT2G28500	-1.931447	-2.649576	-2.130292	0
AT4G08795	0.331608	-2.861224	0.246408	0.972572342
AT1G17820	-0.65622	-11.85063	0.170025	0
AT1G06475	-0.487625	0.031476	-1.023528	9.093667709
AT3G01505	-0.507995	-0.52986	-1.323393	7.259019258
AT5G53870	1.139594	1.494486	2.894371	0
AT1G50960	-0.390534	-0.100209	2.761335	0
AT5G66110	1.938641	1.778945	2.549865	-0.513306714
AT1G03620	0.696866	-0.757155	2.484211	-0.055301255
AT5G59305	1.99043	0.645872	2.459817	0
AT3G51220	1.106283	0.410557	2.439951	-1.088703599
AT3G08115	0.364735	1.400854	2.279186	1.323071685

AT5G22860	0.63266	0.157006	2.274471	-0.247460841
AT3G54500	1.078821	-0.112962	2.244644	0.606639482
AT2G34420	1.30063	1.561258	2.193316	0.757964228
AT5G01190	0.415373	0.269013	2.145637	-0.944432349
AT3G59060	0.805501	0.605044	2.045381	-0.290666595
AT5G10970	0.706097	0.85892	2.020656	0.343564994
AT5G65590	-0.137001	0.327988	2.015755	-1.273619066
AT5G54270	1.27095	1.000833	1.991305	-0.832985289
AT2G22990	0.570851	-0.125485	1.932213	-0.052832927
AT1G78490	-0.230241	0.735902	1.889328	1.019214834
AT5G14920	-0.244733	-0.031665	1.874068	-0.583213668
AT1G48700	1.872304	0.999186	1.854821	0.87081214
AT3G08940	1.343239	0.914953	1.822768	-0.52467976
AT2G15880	0.526643	-0.558661	1.766989	0.558519177
AT5G24770	0.059624	0.46325	1.73224	-2.061168991
AT4G20235	0.227235	0.002962	1.729866	-0.237820592
AT1G52220	0.921127	1.005757	1.707003	0
AT1G72645	0.157112	-0.78838	1.684162	0
AT3G23290	0.428963	0.607816	1.681501	-1.163472208
AT4G18020	0.702199	0.145948	1.675173	-0.088553694
AT4G37970	0.20773	0.689066	1.659296	0.468064536
AT1G07440	0.20685	0.651728	1.656356	0
AT3G56260	0.728228	-0.069501	1.653657	0.390792258
AT3G22410	0.653006	0.52041	1.642291	0.859674429
AT2G35260	0.497515	0.257796	1.640601	-1.778776373
AT1G77090	0.659929	0.250751	1.625851	0
AT1G61520	1.041494	0.851633	1.615083	0.749628933
AT1G02300	0.453041	-1.137317	1.596355	-0.225881305
AT3G54260	-0.020274	0.029985	1.592054	-0.24432672
AT4G27440	0.247898	0.50299	1.573983	-0.072637431
AT4G30270	0.671001	0.50154	1.557407	0.078334839
AT2G20180	0.859105	0.281173	1.546307	-0.385245109
AT4G32810	0.484912	-0.093841	1.541747	-0.373078701
AT5G10946	0.441097	0.255683	1.540585	0.342609245
AT1G20620	0.024038	0.126522	1.533907	-0.032287598
AT1G74470	0.73165	0.496922	1.529785	0.068638522
AT1G22990	1.001714	0.497748	1.52906	-0.56351898
AT2G29650	0.521119	-0.206701	1.527737	0.217180019
AT1G06520	-0.105057	-0.481171	1.524543	0.510064576
AT5G44572	0.483372	0.406414	1.521191	1.043518119
AT3G17040	0.421685	-0.093401	1.517348	0
AT3G50820	1.603625	0.811555	1.51646	0.169301916
AT1G19720	0.016982	-0.447543	1.510887	0.054125123
AT3G16520	0.375066	-0.049271	1.504256	0.190720526
AT2G20260	0.839913	0.860959	1.473664	0.228388473
AT5G23000	0.115475	0.341395	1.468369	-0.784532932
AT3G56940	0.776188	0.261642	1.468103	-0.619040328
AT1G69230	-0.061845	0.315558	1.465253	0.066182422
AT4G35440	-0.036178	-0.207486	1.459887	-0.141449101
AT5G61290	0.712464	-0.016127	1.456518	-0.86571202
AT5G06510	0.29064	-0.241106	1.443136	-0.311369546
AT1G43040	0.392689	1.273184	1.441088	1.51338123
AT4G33000	0.575147	0.215158	1.440218	0.17778838
AT5G01530	0.968329	0.680624	1.434944	0.353136622
AT5G19530	0.339239	0.225235	1.433736	-0.121076306

AT5G54530	0.492463	-0.186808	1.425144	0.12739917
AT5G14370	0.501852	0.48835	1.420105	0.652419648
AT3G56250	0.212898	-0.460384	1.41682	-0.166179836
AT3G53960	0.465325	0.06565	1.416283	-0.317515616
AT3G10405	0.521627	0.280266	1.414471	-0.347777703
AT4G16410	0.642583	0.184013	1.413409	-0.015869641
AT1G06680	0.988996	0.785742	1.407592	0.012015265
AT3G05640	0.457726	0.674223	1.399246	-0.15161072
AT4G23496	-0.092118	0.20912	1.397023	0.509385201
AT3G57190	0.586584	0.46084	1.390429	-1.329927506
AT5G46110	0.64913	-0.14827	1.388981	0.273697115
AT3G20820	0.462912	0.670669	1.387737	0.912020003
AT5G19940	0.705271	0.382812	1.380972	0.137655969
AT1G15820	1.354771	0.914757	1.377911	-0.335867465
AT5G18930	0.489115	0.12322	1.376232	0.325296518
AT1G62770	-0.046204	-0.251073	1.374473	0.269963408
AT1G14700	1.005072	0.250942	1.369828	-1.273272646
AT2G01990	0.193635	-0.058323	1.363386	0.100461954
AT1G21350	0.827783	0.507598	1.362461	0.551847414
AT1G35140	0.042288	-0.91335	1.349759	2.646740218
AT1G25440	0.641907	0.336463	1.348322	0.152926088
AT3G18890	-0.03714	-0.333508	1.346398	-0.805327976
AT4G14540	0.792256	0.933835	1.344746	-0.514063248
AT3G13810	0.212451	-0.06509	1.342162	-0.526665229
AT4G01050	0.412886	0.302804	1.337621	-0.238462018
AT5G49440	0.837223	0.786421	1.335972	-0.080367036
AT1G29240	0.102288	0.045204	1.3332	0.869404459
AT3G28100	0.506909	0.144612	1.313701	-0.585645832
AT2G41190	0.987973	1.035882	1.302392	1.133044019
AT4G13280	-0.088802	-1.089322	1.30126	-1.133867668
AT5G13630	0.77605	0.344641	1.296069	0.137990028
AT4G19420	0.34359	0.388445	1.293309	-0.09236205
AT5G39530	0.400501	0.022973	1.292138	0.604832939
AT1G60000	0.785236	0.639299	1.279184	-0.097080762
AT1G67910	0.279415	0.445343	1.266346	-0.465898755
AT2G33850	-0.437654	-0.025639	1.264442	-0.85471599
AT2G21187	0.971088	-0.06503	1.263317	0.630056478
AT5G43620	0.984969	0.332806	1.262455	0.344158285
AT1G35830	0.179032	-0.102397	1.254928	0
AT1G19540	0.120216	-0.679273	1.254583	0.064419914
AT4G37800	-0.343963	0.05705	1.249899	-0.75089385
AT1G72060	0.783864	0.840417	1.248096	-0.37721307
AT1G76800	0.915193	0.555234	1.24777	0.453180366
AT3G06750	0.206989	0.433817	1.244233	0.48922099
AT1G54200	0.409486	0.070817	1.24345	-0.421397972
AT1G69160	-0.230361	0.494286	1.243144	-0.326743061
AT1G51400	0.632936	0.98513	1.237188	-0.436767549
AT3G50410	0.488188	0.933145	1.23393	1.500306149
AT1G79760	0.567178	0.750221	1.230042	0.238045539
AT5G58330	0.814133	0.299438	1.2262	0.348886656
AT4G18050	0.096768	-0.209308	1.223772	-0.59873191
AT4G16980	0.849697	0.357183	1.221484	0.376690242
AT5G63580	-0.211171	-0.258889	1.219489	-1.080400707
AT5G23380	0.446778	0.384881	1.219268	-0.238427633
AT5G20380	0.222459	0.001994	1.214437	0.19561056

AT2G47400	0.877732	0.645503	1.214201	0.148740013
AT3G01210	0.996717	0.982386	1.213243	0.225914452
AT3G26550	0.374794	-0.21425	1.210039	-0.437573792
AT1G02860	0.399386	0.091789	1.204829	-0.270846996
AT4G14060	0.181189	0.550066	1.201981	-1.547136871
AT1G78290	0.535669	-0.584595	1.199559	0.269698338
AT2G40920	0.365121	0.191641	1.191026	-0.269359499
AT5G51010	0.222921	0.101709	1.190717	-0.241808658
AT2G34490	0.21427	0.654199	1.19071	-1.037840568
AT1G60550	0.432027	0.167062	1.188513	0.098230712
AT4G02290	-0.088897	-0.201689	1.18561	-0.142948813
AT4G37150	0.720732	0.541583	1.185064	0.020182958
AT1G67710	0.129629	-0.14724	1.182976	0.081412645
AT1G54500	0.690758	0.321456	1.182524	0.369238754
AT3G53420	0.875986	0.934039	1.18238	0.209904394
AT4G25080	0.538092	0.418944	1.181532	0.020018853
AT2G31160	-0.038606	0.307836	1.180652	-0.297982163
AT4G08150	-0.308751	-0.801732	1.178785	0.028803273
AT5G18100	0.901144	0.873599	1.177522	0.348945352
AT3G62740	-0.449991	-0.990895	1.176609	-0.291431999
AT3G26220	0.473483	0.741786	1.175099	-0.463098642
AT5G52970	0.576822	0.066938	1.174218	0.740798492
AT1G21680	0.776158	0.916123	1.170407	-0.407570031
AT5G03720	0.357431	0.49085	1.169727	-1.521798607
AT1G52870	0.613885	-0.017462	1.165818	-0.395298724
AT5G35790	0.053746	-0.416993	1.163674	0.458858073
AT1G63240	0.022982	0.180941	1.162656	-0.360957726
AT1G23740	0.905531	0.560388	1.16243	-0.782310981
AT5G60490	0.22705	0.340985	1.161166	0.339450449
AT2G40460	-0.05849	0.266389	1.160919	0.129169856
AT2G21050	0.389172	0.560331	1.156217	-0.134388904
AT1G13700	0.179878	0.242467	1.153634	-0.528845492
AT4G25707	0.715837	-0.591173	1.153609	-0.119041069
AT2G36000	0.276648	0.270256	1.152724	-0.780735028
AT2G40530	0.085359	0.684503	1.152108	-0.849219117
AT3G49160	0.407674	0.365566	1.149831	-0.669696831
AT4G34030	0.381921	-0.189222	1.147261	-0.094159235
AT4G03210	0.27438	0.349073	1.144858	-0.277037286
AT2G39310	0.796634	0.41231	1.143888	-0.317200072
AT5G48775	0.496373	0.094487	1.139513	0.232333796
AT4G37550	0.014822	-0.450955	1.138555	-0.526514136
AT3G25710	0.267232	0.701831	1.137221	-0.12082327
AT1G17460	0.220298	0.436354	1.135091	0.32352161
AT1G68585	0.151129	0.517606	1.131751	-0.035194773
AT5G03680	-0.003905	-0.014054	1.130275	-0.35484041
AT1G64430	0.383341	-0.592398	1.127421	0.129508819
AT5G51110	0.526023	0.174717	1.124292	-0.301145015
AT5G25140	1.187338	0.319569	1.124029	-0.166903484
AT5G48620	-0.014627	-0.791943	1.122992	-0.276514301
AT2G44290	0.815584	0.521021	1.120297	0.115842004
AT5G48800	-0.007023	0.08727	1.117764	-0.57402274
AT3G21090	-0.217742	0.011218	1.117599	0.564947395
AT3G10200	-0.00755	-0.158333	1.117019	-0.263432359
AT4G18780	0.311019	0.522101	1.11528	0.225303668
AT3G03870	0.612235	0.629822	1.107264	0.159894009

AT4G34160	-0.371607	0.2469	1.100149	-0.185380433
AT5G67030	0.830304	0.443737	1.096208	0.143632627
AT1G01770	0.562451	-0.396452	1.096082	-0.26022405
AT1G80060	0.411152	-0.671876	1.091056	-0.07400268
AT3G18080	-0.058705	0.052813	1.090514	-0.412663248
AT4G04630	-0.076865	0.466005	1.090253	-0.504833994
AT3G01860	0.014273	-0.197999	1.089826	-0.69267565
AT5G06290	0.707947	0.796301	1.089032	1.153659869
AT5G14450	-0.025625	0.186384	1.08793	0.720188976
AT3G52060	0.393143	0.286838	1.086754	-0.293437641
AT1G14730	-0.053107	-0.042237	1.083431	-0.333105024
AT5G38850	0.900317	0.598112	1.079738	-0.318855127
AT1G13610	0.033185	0.037454	1.077628	-0.849437878
AT4G19830	0.438466	0.039437	1.074537	-0.191077711
AT4G34480	0.540916	0.559379	1.071441	0.021049692
AT3G53950	0.31544	0.574261	1.071077	0.199201246
AT1G06040	0.512435	0.689355	1.070833	0.755209797
AT3G52840	-0.230314	-0.651849	1.066826	0.259248405
AT4G19950	0.292466	0.1446	1.06373	-0.164741976
AT1G80640	-0.006806	-0.045668	1.062324	-0.507874583
AT3G63210	0.266477	0.179599	1.062119	0.020163726
AT1G06570	0.794424	0.574583	1.059245	-0.26538086
AT4G03230	0.359664	-0.199162	1.058646	-0.476326031
AT3G10080	0.212749	0.435971	1.057967	0.101956149
AT1G02640	-0.090025	-0.408706	1.056406	-0.465324983
AT1G60390	-0.435011	0.20874	1.054871	0.075240791
AT3G47620	0.094997	0.371925	1.048503	0.011860297
AT1G56612	0.267983	-1.017878	1.046803	-0.561264646
AT3G51870	0.126612	0.002867	1.04521	-0.938177194
AT3G20100	0.186556	0.345596	1.044373	-0.492169916
AT5G44190	0.869001	0.039135	1.044094	-0.063606737
AT3G56880	0.535075	0.767061	1.042786	0.107228657
AT3G16800	0.960855	0.823997	1.034041	0.573915044
AT1G80160	0.713231	0.00887	1.030216	-0.53180547
AT2G28410	0.050093	0.609159	1.029851	0.143960401
AT1G17020	0.805223	0.712614	1.029811	-0.085092198
AT2G04850	-0.314067	-0.047729	1.027685	-0.116323702
AT5G01670	0.393297	0.071712	1.022615	-0.172920568
AT1G12280	0.261289	-0.375349	1.02198	0.282140147
AT4G38020	0.391814	0.258935	1.020716	1.09498634
AT2G40640	0.025253	-0.204856	1.018001	0.143446824
AT1G28570	-0.085302	-0.195008	1.014598	0.123501262
AT2G46220	0.560235	0.240478	1.014491	0.109484905
AT1G06473	0.222923	0.246043	1.014149	0.200500791
AT4G28490	0.015485	0.320349	1.011987	-0.149782878
AT4G00780	0.808594	0.87106	1.01163	-0.750695462
AT4G14720	0.337934	-0.157163	1.00986	-0.051498461
AT1G73655	0.773165	0.846885	1.005857	-0.118198894
AT5G07030	-0.077731	0.413172	1.0051	-0.125064133
AT5G40910	0.011163	-0.918527	1.005064	-0.074463297
AT2G33060	-0.014317	-0.362159	1.004949	0.350731517
AT1G55510	0.341459	-0.334327	1.002116	0.156787635
AT4G21480	0.507319	0.706678	1.001602	-0.423631362
AT5G52882	-0.699737	-0.576085	-1.003833	0.053846933
AT5G61030	-0.725369	-0.000411	-1.003987	-0.043708384

AT4G01480	-0.352909	-0.121765	-1.005531	0.005864574
AT1G62380	-0.373213	-0.233535	-1.011113	-0.023370365
AT3G46320	-0.349408	0.368548	-1.011216	0.211591175
AT5G39320	-0.438403	0.010571	-1.013604	0.154477545
AT1G02920	-0.309434	0.351583	-1.014248	-0.136264684
AT1G12110	-0.273042	0.225472	-1.014991	0.161319647
AT5G63980	-0.590189	-0.211657	-1.016296	-0.337159627
AT1G74030	-0.344348	-0.166027	-1.018521	-0.129525056
AT5G61440	-0.693343	-0.369178	-1.019958	-0.270059606
AT5G45280	0.144677	0.394378	-1.022004	-0.049956088
AT1G10682	-0.525008	-0.572414	-1.022129	0.006551152
AT4G04940	-0.779004	-0.614739	-1.022176	-0.127472344
AT2G30930	-0.468159	-0.113153	-1.023511	0.136861297
AT3G09940	-0.128696	-0.1534	-1.023674	0.115694644
AT4G19720	-0.071226	-0.379081	-1.024658	-0.696320212
AT3G18130	-0.414834	0.02656	-1.026965	0.369900274
AT5G59850	-0.225304	0.41779	-1.02821	0.224534878
AT5G01765	-0.466644	-0.539178	-1.031386	0.546645429
AT5G40590	-0.343409	-0.096735	-1.03603	-0.868525617
AT1G58170	-0.755017	-0.848633	-1.036958	-0.472383486
AT2G19690	0.165206	0.557111	-1.039151	-0.495976932
AT1G22065	-0.839121	0.077842	-1.043617	0.017234613
AT1G31280	-0.86586	-0.931003	-1.04458	0.264668903
AT3G27880	-0.999341	-0.389642	-1.049335	-0.038738135
AT1G44800	-1.114576	-0.708438	-1.049662	0.007204168
AT5G66390	-0.07259	-0.219765	-1.049713	0.037730914
AT4G11290	-0.236073	-0.069274	-1.055499	-0.086871301
AT5G52220	-0.126247	0.244602	-1.056668	-0.315328911
AT2G07687	0.079201	0.617206	-1.056696	-0.539884518
AT5G14750	-0.335465	0.009322	-1.064772	0.169320482
AT4G13430	-0.822834	-0.516822	-1.06547	0.034004296
AT4G25630	-0.540412	-0.402291	-1.069257	0.163776644
AT2G04170	-0.272251	-0.172649	-1.069834	-0.035034942
AT2G22000	-0.355413	-0.407289	-1.070952	-0.312135911
AT1G20696	-0.318415	-0.173146	-1.073718	0.004780555
AT1G22410	-0.60096	-0.429595	-1.074632	-0.021984391
AT3G48340	-0.010424	0.115815	-1.074635	0.05364792
AT5G09530	0.018296	-0.318781	-1.075279	-0.911793873
AT2G28960	-0.611006	-0.76031	-1.077689	-0.173613271
AT5G09800	0.110317	0.134999	-1.081617	0.730674366
AT2G46410	0.128938	-0.013193	-1.082746	0.356805292
AT2G46140	0.241761	0.530094	-1.084738	-0.044863823
AT1G61360	-0.487382	-0.23781	-1.085469	0.064257922
AT5G44820	-0.295471	-0.576406	-1.087169	0.453722267
AT5G45670	-0.967574	-0.349439	-1.088216	0.252920285
AT1G08580	-0.557202	0.055793	-1.088454	0.263779594
AT1G71750	-0.719829	-0.211822	-1.088701	-0.32407216
AT5G59970	-0.116813	0.373121	-1.09156	0.280704334
AT5G23190	0.496743	-0.332132	-1.09234	0.50511715
AT4G35630	-0.585708	-0.089477	-1.093117	-0.166218915
AT1G66160	-0.212923	-0.027776	-1.093756	0.090709781
AT4G34810	0.201513	0.195488	-1.093872	0.503267661
AT3G08520	-0.061271	0.821878	-1.093992	-0.075460124
AT5G52250	-0.239435	-0.003999	-1.098588	-0.734439068
AT3G62460	-0.896746	-0.362422	-1.098762	0.604528228

AT4G19550	-0.590538	-0.899261	-1.09881	-0.636457686
AT5G26340	-0.186389	-0.279942	-1.100932	0.186609292
AT1G26270	-0.657272	-0.745498	-1.100977	-0.068899635
AT5G35320	-0.912165	-0.828944	-1.102481	0.088740625
AT1G52245	-0.493859	-0.641183	-1.103713	0.62669187
AT3G54640	-0.606745	-0.460138	-1.103839	-0.260550684
AT1G74385	-0.376624	0.548171	-1.10503	0.500208839
AT1G34760	-0.174756	-0.744007	-1.105887	-0.596307073
AT4G26200	-0.483076	-0.087907	-1.106622	1.689302913
AT4G30320	0.419793	-0.033264	-1.11723	0.553876244
AT1G71695	-0.075332	0.0249	-1.121676	-0.192060606
AT2G47550	-1.035152	-0.89166	-1.126822	0.031040395
AT2G19590	-1.045345	-0.881076	-1.126884	0.224453384
AT2G29460	-0.390892	0.00111	-1.128426	0.465366314
AT5G65040	-0.336172	-0.41176	-1.130167	-0.121975257
AT4G15270	-0.136372	-0.250593	-1.130419	0.197167625
AT2G32960	-0.641741	-0.255659	-1.130692	0.212830279
AT2G04400	-0.528605	-0.724726	-1.131472	0.013709771
AT3G13610	-0.441872	0.013884	-1.134385	-0.074639695
AT4G11350	-0.205706	-0.571025	-1.136686	-0.013323737
AT2G29450	-0.411186	-0.564673	-1.139992	0.037771918
AT2G47140	0.18309	0.129201	-1.146233	0.470284236
AT1G43800	0.283288	-0.455571	-1.146867	0.619764156
AT1G68825	-0.346226	0.009526	-1.150447	0.460526475
AT1G19020	-0.587149	0.238963	-1.150578	0.085993876
AT1G66280	-0.513607	-0.258949	-1.152461	0.120330744
AT3G54625	-0.050197	-0.2412	-1.153647	0.287770437
AT5G60950	0.031556	0.295487	-1.158116	0.218374053
AT5G04310	-0.554915	0.302381	-1.158729	0.117151015
AT4G02270	0.259243	0.090831	-1.15997	0.277805567
AT3G20015	-0.75352	-0.474009	-1.164072	-0.004213161
AT1G55205	-0.670885	-0.150284	-1.164256	-0.286122201
AT3G50900	-0.340171	0.186507	-1.168765	0.47195944
AT5G28640	-0.599287	-0.680451	-1.169772	0.450647136
AT1G17190	-0.322125	0.076727	-1.171452	0.252651083
AT2G31790	-0.865856	-0.491292	-1.172511	-0.228647889
AT3G28930	-0.571929	-0.121519	-1.173095	-0.425900223
AT3G48460	-0.037895	0.068143	-1.173926	-1.870767573
AT1G68850	-0.001365	-0.445818	-1.177788	0.341554879
AT2G15310	-1.13716	-0.04377	-1.177919	-0.313502495
AT5G15520	-0.169621	0.293387	-1.181393	-0.221746244
AT1G26840	-0.521109	0.164342	-1.181743	0.083118916
AT1G66270	-0.45485	-0.324711	-1.182712	0.215965358
AT5G48540	-0.54742	0.031949	-1.183747	0.22587769
AT5G11740	-0.684324	-0.131995	-1.188024	0.19201932
AT1G26255	-1.02877	0.153535	-1.188189	-0.866274908
AT3G25717	-0.306344	-0.784785	-1.188333	0.144902567
AT1G63410	-0.052262	-0.232266	-1.188565	0.232532917
AT3G46940	-0.38016	0.455131	-1.188807	0.576284426
AT1G05250	-0.147639	-0.347063	-1.189229	-0.033384112
AT3G13520	-0.581113	0.359199	-1.189296	0.178209538
AT4G37490	-0.591957	-0.481515	-1.189817	0.511682103
AT1G11540	-0.618008	0.14583	-1.191437	-0.126659572
AT1G09932	-0.395267	-0.460503	-1.191929	-0.054619669
AT1G62980	0.035745	0.105572	-1.19959	0.091185825

AT3G56020	-0.011258	0.552336	-1.203741	0.153068985
AT1G14220	-0.384914	0.316435	-1.20433	0.243656672
AT2G43510	-0.047103	-0.020622	-1.20444	0.638170478
AT2G38110	-0.277747	-0.917491	-1.208126	-0.294699347
AT1G74830	-0.069194	-0.058	-1.210804	0
AT2G46600	-0.422988	-0.095215	-1.2111	0.104723514
AT5G48880	-0.861453	-0.837328	-1.211789	-0.494674144
AT3G11120	-0.230915	0.217298	-1.216048	0.345981651
AT3G03130	-0.804599	-0.569645	-1.216221	0.3720346
AT1G77330	-0.032805	-0.138988	-1.221829	-0.174181999
AT3G23325	-0.410142	0.351365	-1.223069	-0.054269024
AT4G23700	-0.542114	-0.345232	-1.224641	-0.224973513
AT5G63270	-0.175183	0.173644	-1.224882	-0.237646429
AT4G30530	-0.49409	-0.304954	-1.225805	0.076060568
AT4G30140	-0.113795	-0.927298	-1.226201	-0.061320075
AT5G25250	-0.70974	-0.17965	-1.226273	-0.318102952
AT5G24850	-0.465304	-0.94882	-1.226814	-0.180364411
AT5G43540	0.385203	-0.454475	-1.228133	-0.023757569
AT1G11185	-0.554009	-0.245688	-1.228693	-0.823300989
AT2G22860	-0.188478	-1.048143	-1.231144	-0.393912897
AT1G26250	-1.167409	-0.038312	-1.231583	-0.414875856
AT5G60520	0.109471	0.614623	-1.232771	0.938981446
AT5G40510	-0.269672	-0.03132	-1.233106	0.054765554
AT2G27840	-0.738717	-0.401187	-1.233954	0.543549414
AT1G05260	-0.34733	-0.310905	-1.237154	-0.217838293
AT3G55120	-0.774614	-0.740433	-1.239613	1.153603494
AT4G24140	-0.319119	-0.807269	-1.239617	-0.429415233
AT5G23830	-0.615177	-0.100685	-1.241291	0.229025357
AT5G49910	-0.79373	-0.871565	-1.244471	0.119314835
AT5G64810	-0.328181	0.313978	-1.245803	-0.221525671
AT5G42510	-0.260026	0.139823	-1.248525	-0.454265864
AT2G47540	-0.48787	0.090496	-1.249415	-0.367253037
AT2G16760	-0.022977	0.068446	-1.25077	0.161938566
AT4G29690	-0.465519	0.028149	-1.254194	0.087956765
AT5G06990	0.093692	0.093449	-1.255148	-0.400407754
AT5G38900	-0.113614	0.110578	-1.263354	-1.185744029
AT2G30130	-0.39951	0.359922	-1.264639	-0.222477217
AT4G26010	-0.115705	-0.271572	-1.265105	0.182042236
AT2G45860	-0.139827	-0.450013	-1.265345	0.221263411
AT1G01600	0.138296	0.04705	-1.266002	-0.04089042
AT2G37360	-0.3305	-0.825433	-1.268737	-0.210109414
AT1G02360	-0.699947	-0.328034	-1.271276	-0.18674406
AT1G24575	-1.128757	-0.382125	-1.27254	0.819294189
AT4G00680	-0.166247	-0.167754	-1.272624	0.526676638
AT4G24275	-0.424353	-0.054816	-1.27514	-0.202167641
AT5G58860	-0.236437	-0.678497	-1.278697	-0.526219613
AT3G60330	-0.36491	-0.826923	-1.281548	-0.172332343
AT3G12965	-0.202022	0.227662	-1.282509	0.393290887
AT2G23540	-0.249714	-0.701946	-1.289491	-0.361455433
AT1G33280	0.23062	0.50757	-1.294979	0.654931447
AT1G26800	-0.78978	-0.701159	-1.298158	0.09663277
AT3G14940	-0.423203	-0.415379	-1.298165	0.055714553
AT4G13310	-0.316742	-0.679158	-1.298473	-0.63592553
AT5G18290	-0.730218	-0.585263	-1.298973	0.350410272
AT1G05730	-0.151054	0.291762	-1.303067	-0.383484562

AT5G59090	-0.378556	-0.482282	-1.305749	-0.197528617
AT5G50300	-0.52583	-0.335798	-1.308902	-0.060272013
AT5G24140	0.139757	-0.642058	-1.313223	1.160261613
AT5G43870	-0.766309	-1.150558	-1.314545	-0.122963866
AT2G04025	-0.35416	-0.690583	-1.315947	0.105997554
AT1G74460	-0.178428	-0.262872	-1.316702	-0.960576635
AT4G15280	-0.234832	-0.214684	-1.319112	-0.750580744
AT1G14160	0.077449	-0.643929	-1.320432	0.220740616
AT5G09480	-0.464222	-0.800085	-1.322322	-0.014856181
AT4G19980	-0.827857	-0.097633	-1.323659	-1.173449537
AT5G58750	-0.206705	-0.683571	-1.324183	-0.086359565
AT3G51600	-0.307893	-0.045323	-1.327362	0.246886198
AT2G02950	-1.164171	-0.763088	-1.331786	-0.076633537
AT4G24670	-0.355472	-0.195334	-1.332132	0.312123869
AT1G23140	-0.668682	-0.532696	-1.332357	0.318703792
AT1G53680	-0.266201	-0.167965	-1.33259	0.177759119
AT5G67400	-0.351005	-0.374817	-1.335297	0.171373728
AT4G24930	-0.127288	-0.474877	-1.337499	0.133226869
AT1G66200	-0.213474	-0.175217	-1.343165	-0.156931029
AT3G17611	-0.803514	-0.596808	-1.34406	0.871316726
AT1G23410	-0.271271	0.433294	-1.346887	-0.075605161
AT1G13420	-1.373466	-1.005055	-1.34779	-0.442012408
AT4G02520	-0.75067	-0.497679	-1.348079	-0.246145988
AT1G20160	-0.213395	-0.021166	-1.352141	0.034364117
AT2G32220	-0.089048	0.455298	-1.353531	0.455164124
AT3G48450	-0.752378	-0.879649	-1.356452	-0.965563998
AT4G32950	-1.661293	-0.627078	-1.36037	0
AT5G06740	-0.922793	-0.337777	-1.364131	0.155211038
AT4G19030	-0.227653	0.154186	-1.366627	-0.025777444
AT1G79075	0.102312	0.802444	-1.379466	0.268084612
AT4G25220	0.067921	-0.392316	-1.384452	0.635905536
AT5G43380	-1.181741	-1.189757	-1.392323	0.868838956
AT5G44110	-0.323607	-0.705925	-1.408296	0.801179699
AT4G14640	-0.535947	-0.099465	-1.41003	-1.255559919
AT2G46650	-1.04725	-0.107029	-1.41081	-0.144070418
AT5G54165	-0.383693	-0.192512	-1.420163	0
AT1G20693	-0.975327	-0.997029	-1.424106	-0.065158892
AT1G74010	-1.018436	-0.331337	-1.432017	0.060797687
AT5G23360	-0.946008	-0.293912	-1.433347	-0.01070891
AT1G79470	-0.73826	-0.557143	-1.438448	-0.39391664
AT2G16060	-0.893233	-0.170502	-1.442934	0.020226757
AT1G53950	-0.342425	-0.060957	-1.444144	0.373754605
AT3G45430	-0.34339	-0.129175	-1.445823	-0.227937776
AT3G51680	-0.475167	-0.391369	-1.447665	0.809163723
AT2G29440	-0.760443	-0.70484	-1.448169	0.103354976
AT1G54000	-0.77653	-0.477076	-1.449895	-0.143683513
AT4G26790	-0.147009	-0.943896	-1.452342	-0.404132295
AT2G38750	-0.075107	-0.187432	-1.454638	0.321585607
AT4G08780	-1.42417	-0.613627	-1.457832	0.346085458
AT1G01480	-1.009953	-0.913606	-1.459451	-1.227464371
AT4G22080	-0.283264	-0.743388	-1.461666	-0.505929554
AT3G59068	-0.928791	0.418123	-1.4628	-0.450221383
AT1G13590	0.249405	0.061341	-1.469072	-0.154072141
AT3G09680	-0.627034	-0.051564	-1.470426	-0.955705505
AT5G22410	-0.440303	-0.15878	-1.471469	0.227338772

AT1G63930	-1.023485	-0.54892	-1.473041	-0.059585872
AT1G01453	-0.862903	0.214227	-1.479461	0.327015574
AT2G20562	-0.423049	0.642782	-1.479533	0.398845787
AT1G66800	-0.232282	-0.759934	-1.479735	-0.039944892
AT5G23990	-0.280192	-0.794847	-1.491806	0.625153839
AT5G10210	-0.731991	-0.741933	-1.494035	0.005975893
AT2G18450	-0.254817	-0.490167	-1.498533	0.51350458
AT3G54150	-1.001798	-0.471404	-1.500805	-0.016074761
AT4G36880	-0.262669	-0.258658	-1.505119	1.34666873
AT1G57590	-0.41021	-0.218837	-1.509369	0.283818982
AT1G01750	-0.493761	-0.085749	-1.514348	-0.332261179
AT3G48520	-0.387937	-0.169461	-1.516356	1.34828096
AT3G19710	-1.279086	-0.474711	-1.518201	0.040304374
AT4G18510	-0.71864	-0.656194	-1.520631	0.055549673
AT5G56030	-0.929982	-0.741101	-1.522002	-0.024858354
AT5G57220	-0.895007	-0.336094	-1.523415	-0.061684084
AT3G16390	-0.652025	-0.999407	-1.523894	0.235411385
AT3G20557	0.248056	-0.728987	-1.524183	0.682227777
AT3G11930	-0.745273	-0.575158	-1.527243	0.036574182
AT3G61930	-0.064486	-0.524583	-1.527633	-1.64200393
AT3G27400	-1.270981	-0.777905	-1.544013	0.241267769
AT4G08770	-0.915895	-0.462169	-1.550862	0.310432026
AT1G18100	-0.44798	-0.547669	-1.553649	0.715000567
AT5G44550	-0.437932	-0.766893	-1.554721	-0.475663727
AT5G02575	-1.352987	-0.013261	-1.589974	0.239273484
AT2G21100	0.203899	-0.051823	-1.594506	0.051829833
AT2G43870	-0.476255	-0.467621	-1.607821	-0.976344499
AT5G23810	-0.64858	-0.913324	-1.611494	2.027977712
AT4G38410	-0.045714	-0.293254	-1.615066	0.678225926
AT2G08685	-0.844001	-0.899336	-1.616767	-0.352995118
AT2G43120	-1.162381	-0.483305	-1.622824	0.321729551
AT5G47220	-0.393428	-0.020605	-1.624514	-0.404108406
AT5G14200	-1.034635	-0.415342	-1.633721	0.096436826
AT3G14440	-1.067907	-0.027907	-1.639172	0.651555802
AT5G54490	-0.578246	-0.435977	-1.660973	-0.101430381
AT3G54600	-1.065837	-0.386336	-1.664351	0.103181171
AT1G17180	-0.910394	0.223524	-1.666048	0.562040215
AT5G39580	-0.582232	-0.451935	-1.678661	0.226127115
AT1G55290	-0.783956	-0.571483	-1.71282	0.237045892
AT4G21840	-0.213773	-0.330285	-1.713422	-1.394798525
AT5G04340	-0.859531	-0.806675	-1.717022	-0.235395148
AT5G57530	-0.470207	-0.480109	-1.718936	0.273982132
AT4G13770	-1.279279	-0.400674	-1.727271	-0.284361822
AT1G59725	-0.367103	-0.672414	-1.735547	-0.74247195
AT5G59240	-0.434984	-0.299292	-1.746568	0.313107336
AT1G51620	-0.768475	-0.746752	-1.759244	-0.431235956
AT1G65500	-0.319553	-0.189385	-1.76953	0.122683101
AT3G24500	-0.85451	-0.679065	-1.771902	-0.705262895
AT1G15385	-0.268757	-0.173941	-1.788369	-0.669675303
AT5G06090	0.394214	-0.97989	-1.789921	0.390922191
AT3G62930	-0.550487	-0.440237	-1.803704	0
AT2G37430	-0.898448	-0.117271	-1.804588	-0.185381349
AT5G05270	-0.996613	-0.966598	-1.812561	0.833361493
AT3G09405	-1.912584	-1.249647	-1.825553	0.380521895
AT4G22710	-1.718835	-1.673865	-1.832908	-0.383231308

AT3G03190	-1.160291	-0.442631	-1.850335	-0.058897781
AT3G07490	-1.294842	0.266846	-1.857478	-0.070370752
AT1G76650	-1.277792	-1.276945	-1.862554	0.677224119
AT4G28110	-0.391396	-1.335837	-1.878259	0.891938684
AT5G02780	-1.393445	-0.795787	-1.887235	-0.077959774
AT5G36130	-0.556029	-0.644655	-1.903842	0
AT5G15022	-1.0752	-0.314268	-1.90828	1.186745408
AT1G07795	-0.097215	-0.073604	-1.914039	0.915409404
AT3G04300	-0.636858	-0.722204	-1.919134	1.214349086
AT4G10510	-1.508189	-1.040651	-1.932355	0.742211045
AT3G27070	-1.032238	-0.372779	-1.938413	0.495474484
AT4G22217	-0.068251	-0.053639	-1.944659	-0.526654174
AT2G22880	0.488381	-0.00757	-1.954498	-0.047892244
AT1G73965	-1.012631	-0.146958	-1.960279	0.05696065
AT3G16150	-0.757497	-0.999723	-1.967137	-0.483167499
AT5G07990	-0.982221	-1.040808	-1.97735	3.228407009
AT1G14205	-0.226287	-0.823596	-2.006	0
AT4G18170	-0.924619	-1.046366	-2.010767	1.249458327
AT3G22740	-1.599079	-1.355851	-2.018625	-0.405991416
AT5G56010	-0.988266	-0.819622	-2.02405	-0.01843075
AT1G68350	-0.786709	-1.248299	-2.038695	0.504883111
AT4G22470	-0.520903	-1.202701	-2.047322	0
AT1G30990	-0.65665	-1.083548	-2.053779	0.143818056
AT1G08090	-1.495723	-1.275809	-2.058152	-0.628279338
AT1G10370	-0.435806	-0.855786	-2.072147	-0.494311382
AT4G06880	0.223606	-0.759203	-2.072211	0.844148769
AT5G11440	-0.510266	-0.540656	-2.086823	0
AT1G02930	-0.869295	-0.219465	-2.125705	-0.121108238
AT2G43000	-1.604386	0.137446	-2.12587	-0.054452648
AT5G04120	0.531092	-0.185082	-2.130739	-0.859571674
AT3G26830	-1.212772	-0.901302	-2.143684	0.089844307
AT1G62280	-1.039578	-0.728218	-2.16197	-0.075871633
AT1G13480	-0.744165	-0.968796	-2.168257	-1.035438379
AT1G21520	-0.539408	0.293497	-2.238988	1.543998557
AT3G14260	-1.000973	-0.974582	-2.329865	-0.410880015
AT2G41480	-1.211264	-0.922386	-2.334335	0.650977899
AT2G27402	-0.04442	-0.691739	-2.335108	0.171379479
AT5G59990	-0.438297	-1.231588	-2.34669	0.618429217
AT1G67980	-1.302711	-1.180795	-2.408602	0.063707327
AT2G28210	-1.715206	-1.593851	-2.434109	0.638267053
AT4G11393	-1.124004	-0.781506	-2.44866	0
AT2G08780	0.249838	0.710313	-2.514109	-0.038130075
AT4G22214	-0.495625	-0.377018	-2.62041	0.469555329
AT3G05950	-1.76391	-0.725934	-2.668328	0
AT3G60270	-1.844716	0.247969	-2.699636	0.475574729
AT3G47380	-1.165843	-1.506818	-2.723293	0.458667167
AT4G06775	-0.594441	-1.881976	-2.742255	-0.361314958
AT2G27535	-2.010253	-2.14808	-2.75906	0
AT5G56080	-1.72611	-1.919855	-2.769361	-0.288638127
AT1G68290	-1.591188	-1.140805	-2.7715	0
AT1G22900	-1.188473	-1.17909	-2.828529	-0.161261914
AT1G10585	-1.048153	-0.483236	-2.858729	0
AT5G22555	-1.235759	-1.306719	-2.96925	-0.564026749
AT2G02010	-2.670365	-1.759057	-3.038603	-0.788083096
AT5G43068	-0.546381	-0.945592	-3.052143	0

AT5G61890	-0.417987	-1.362108	-3.078779	0
AT4G33905	-0.408598	0.05935	-3.081623	0
AT3G55970	-2.754826	-2.353135	-3.120978	0
AT4G31970	-2.491997	-0.476889	-3.279442	-1.318628689
AT2G24195	-0.835773	-1.951897	-3.281274	0
AT2G30750	-2.586494	-0.904165	-3.430716	-0.512856249
AT1G69930	-1.833372	-1.286339	-3.437539	0.289415475
AT1G49570	-0.683303	-1.372928	-3.666709	-0.094238154
AT5G05340	-0.266232	-0.35025	-3.689902	0
AT1G69920	-1.587861	-2.472659	-4.799018	-0.050034141
AT5G52390	-1.383048	-2.066484	-5.359069	0
AT3G52605	0.402542	0.378059	0.039637	10.2686248
AT1G79850	-0.025024	-0.469899	1.144333	10.13488563
AT5G57560	-0.789868	-0.794768	0.217875	2.332042863
AT1G25055	0.475984	0.978678	-0.745652	1.398793876
AT5G45340	-0.006655	-0.780669	1.013856	1.149842454
AT1G25211	0.250596	0.319015	-0.064487	1.069870501
AT2G16740	-0.627272	-0.297824	-0.417738	1.027990995
AT4G17500	0.374438	0.571996	0.156557	-1.022119179
AT2G40330	-0.313502	0.290165	-0.692255	-1.055713604
AT4G26130	-0.481322	-0.195718	0.02145	-1.060006877
AT5G25350	0.341411	0.22982	-0.03036	-1.143772258
AT3G19840	-0.105376	-0.416687	0.370038	-1.180497446
AT4G23510	0.092171	-0.304776	0.149483	-1.190812728
AT1G24881	-0.053372	0.100875	0.208275	-1.777649373
AT4G26095	0.1022	1.761681	1.828625	-4.052718524
AT5G01210	-0.583482	-0.92905	-0.884269	-12.28145066

Supplementary Table 2. Annotation and t-SNE clustering of DEGs between Flight and Ground Control in Arabidopsis roots or leaves from human-tended VG suborbital spaceflight experiments. Log2FC numbers highlighted in yellow indicate a significant change (Padj < 0.05). Red indicates upregulation (Log2FC > 1) and blue indicates downregulation (Log2FC < -1). DEGs belonging to each functional category are highlighted by purple bars, and those with significant changes in APEX03/APEX04 spaceflight are highlighted by maroon bars.

DEG	F1R vs GR	F2R vs GR	F3R vs GR	F1L vs GL	F2L vs GL	F3L vs GL	Gene name	Annotation	t-SNE cluster	ROS	ROS core I	ROS core II	ROS core III	ROS core IV	ROS core V	ROS core VI	ROS core VII	ROS core VIII	Heat	Cell Wall	House keep-ing	Flower-ing	APEX03-APEX04
AT4G38470	-1.004229016	-1.300692072	-0.527516444	0.054233036	-0.160342277	0.15749905	STY46	Serine/threonine-protein kinase STY46 [Acc:F4JTP5]	0	■													
AT3G43790	-1.062923066	-1.029713549	-0.052412049	-1.258548312	-0.728034802	-0.177798268	ZIFL2	Probable peptide/nitrate transporter At3g43790 [Acc:Q3EAQ5]	0														
AT1G71880	-1.131434295	-1.19613376	-0.405212085	-0.631997804	-0.674155322	-0.901055451	SUC1	Sucrose transport protein SUC1 [Acc:Q39232]	0	■			■										■
AT4G18340	-1.265080759	-1.093341039	-0.368570551	-0.284098239	0.644450215	0.027877091	AT4G18340	Beta-1,3-glucanase-like protein [Acc:Q8L837]	0	■										■			
AT2G37180	1.051639096	0.883943405	1.638513643	0.896297566	1.350842936	0.937175922	PIP2-3	Aquaporin PIP2-3 [Acc:P30302]	0										■				■
AT4G30470	1.43266176	0.572561639	-0.05224616	0.50025739	1.336804646	0.574992112	AT4G30470	Cinnamoyl-CoA reductase-like protein [Acc:Q9M0B3]	0														■
AT1G62370	1.22527897	0.992607995	0.297362421	0.486514688	1.593867023	0.991004748	AT1G62370	F2401.10 [Acc:O48801]	0														
AT3G26450	1.086116532	0.559805786	0.887745044	0.378628456	1.187027966	0.56876396	AT3G26450	Major latex protein, putative [Acc:Q9LIN0]	0	■													
AT2G44670	-1.01296389	-0.429061778	-0.856807931	-0.595872127	-1.62265329	-1.02671282	FLZ3	FCS-Like Zinc finger 3 [Acc:O80506]	0														■
AT1G49870	-1.040771322	-0.831065167	-0.727987394	-0.351315103	0.834883514	-1.270010843	AT1G49870	Myosin-2 heavy chain-like protein [Acc:Q9C6C7]	0														■
AT2G01020	-0.531923379	3.352588756	-2.474923314	-0.329456963	0.012112388	-0.754394956	AT2G01020	rRNA [AT2G01020]	0														
AT3G41979	-0.531923374	3.352588755	-2.474923315	-0.329456948	0.0121124	-0.754394942	AT3G41979	rRNA [AT3G41979]	0														
AT2G01010	0.606197397	1.901027061	-1.162058099	-0.341647958	-0.03986117	-0.826204118	AT2G01010	rRNA [AT2G01010]	0														
AT3G27030	0.045082205	1.389664313	-1.171363049	-0.712133484	0.144944734	-0.09013618	AT3G27030	Transmembrane protein [Acc:F4JEW3]	0														
AT1G64220	-0.037902301	1.348991527	-1.192297423	0.540089783	0.089269942	-2.126117848	TOM7-2	Mitochondrial import receptor subunit TOM7-2 [Acc:Q3ECI7]	0														
AT2G46240	-1.040706131	-1.093710254	-0.896475686	-1.796870132	-1.496017573	-2.261192585	BAG6	BAG family molecular chaperone regulator 6 [Acc:O82345]	0	■			■										
AT2G30600	-0.777868319	-1.350014555	-0.291541106	-0.313274098	-1.052866737	-0.221309372	AT2G30600	BTB/POZ domain-containing protein [Acc:F4INV5]	0										■				
AT1G48360	-0.20213011	-1.148494258	-0.176184228	-0.324678051	-1.183905696	-0.422141236	FAN1	Fanconi-associated nuclease 1 homolog [Acc:Q5XVJ4]	0														
AT2G32250	-0.326226157	-1.199315925	0.101556004	-0.460184156	-1.066581205	-0.64093144	FRS2	Protein FAR1-RELATED SEQUENCE 2 [Acc:Q3EBQ3]	0	■													
AT2G37280	-0.925639831	-1.115964303	-0.231887261	-0.813599016	-1.657536656	0.540788516	PDR5	pleiotropic drug resistance 5 [AT2G37280]	0														
AT1G01940	0.920069225	1.388417796	0.90636133	0.89794666	0.8093721	1.102037755	CYP18-1	Peptidyl-prolyl cis-trans isomerase CYP18-1 [Acc:Q9LPC7]	0	■			■						■				
AT5G17350	0.843382666	1.129436668	0.197906057	0.533688079	0.187572021	1.106371449	AT5G17350	Uncharacterized protein At5g17350 [Acc:Q9LF49]	0	■			■										
AT5G06845	0.362118537	2.02768782	-0.093713766	-0.231227302	-0.357605668	-1.820980387	AT5G06845	None	0														
AT1G48750	0.148833165	1.141882228	-0.175390986	-1.149900554	-0.834409933	-1.39424333	AT1G48750	Bifunctional inhibitor/lipid-transfer protein/seed storage 2S albumin	0														■
AT1G17820	-0.656220076	-11.85062769	0.170025118	-10.86308127	0.798809548	-0.067396265	AT1G17820	At1g17820 [Acc:Q8L7M7]	0														
AT1G06002	-0.905895392	-12.31422924	-0.889453834	-2.472788373	-0.092208778	-4.34008455	AT1G06002	Potential natural antisense gene, locus overlaps with AT1G06000	0														
AT3G05640	0.457726152	0.674222901	1.399246414	0.321707682	1.543910446	0.613212164	AT3G05640	Probable protein phosphatase 2C 34 [Acc:Q9M9W9]	0														
AT3G63210	0.266476668	0.179598679	1.062118683	0.047925857	1.284655542	0.870927685	MARD1	Protein MARD1 [Acc:Q8LGS1]	0	■			■										
AT3G25717	-0.306343759	-0.784785104	-1.188333028	0.70762458	1.238354536	0.198318805	RTFL16	DVL6 [Acc:Q6IM95]	0														
AT1G21520	-0.539408377	0.293497101	-2.238988422	-0.387641533	1.178708583	0.18524496	AT1G21520	Uncharacterized protein At1g21520/F24J8_4 [Acc:Q8GYE6]	0	■													
AT3G13520	-0.581112762	0.35919906	-1.189296171	0.208735721	1.109101763	0.258074244	AGP12	Arabinogalactan protein 12 [Acc:Q9LJD9]	0										■	■			
AT2G29440	-0.760442815	-0.704840336	-1.448169421	-0.026580833	1.360883761	-0.469057144	GSTU6	GSTU6 [Acc:A0A178VUQ0]	0														
AT5G43620	0.98496945	0.332805648	1.262455027	1.233516199	0.103539213	1.37217085	PCFS5	Polyadenylation and cleavage factor homolog 5 [Acc:Q9FIX8]	0														
AT3G49160	0.40767398	0.365566045	1.149831426	0.830651576	-1.360120042	1.313315139	PKP4	Plastidial pyruvate kinase 4, chloroplastic [Acc:Q9M3B6]	0	■			■										
AT2G21790	-0.676207726	-0.62446296	-0.682216023	-1.002780151	-1.066522184	-1.008946121	RNR1	Ribonucleoside-diphosphate reductase large subunit [Acc:Q9SJ20]	0	■													
AT5G49030	-0.23824475	-0.548306523	-0.575506298	-1.044591733	-1.045980965	-1.027054667	OVA2	tRNA synthetase class I (I, L, M and V) family protein [Acc:F4K4Q2]	0														
AT5G02490	0.087483878	0.538440701	-0.939865241	-1.333953078	-1.39178367	-1.923316033	MED37D	mediator of RNA polymerase II transcription subunit 37c [Acc:P22954]	0	■			■						■				■
AT1G74300	-0.20978292	-0.455503365	-1.036114533	-2.8506271	-2.937661554	-3.820969708	AT1G74300	Alpha/beta-Hydrolases superfamily protein [Acc:Q9C915]	0	■													
AT1G27420	0.533516617	0.067598516	0.460173974	1.248283043	1.066963674	0.975089456	AT1G27420	Putative F-box/kelch-repeat protein At1g27420 [Acc:Q9FZJ3]	0														
AT2G39350	0.131282165	-0.577866577	-0.555513484	1.227901053	1.242153291	0.77649365	ABCG1	ABC transporter G family member 1 [Acc:O80946]	0														
AT5G66080	0.756247774	0.263186841	-0.232434522	1.212899686	1.595302472	1.629365423	AT5G66080	Probable protein phosphatase 2C 79 [Acc:Q9FKX4]	0										■				
AT2G42870	0.104326965	0.786138223	-0.628126826	1.147843756	1.161636535	0.922739865	PAR1	Transcription factor PAR1 [Acc:Q9SJH0]	0	■													
AT4G37370	0.160563986	1.269145597	0.03126124	1.136594347	1.039222123	0.616045629	CYP81D8	Cytochrome P450, family 81, subfamily D, polypeptide 8 [Acc:Q9SZT7]	0	■			■	■	■		■						
AT2G21640	0.416889143	1.513249018	-0.258984933	1.117225845	1.403743536	0.571243212	AT2G21640	Marker for oxidative stress response protein [Acc:Q9SIJ6]	0	■			■										
AT5G38200	0.581287976	0.366433445	0.924228133	1.081262128	1.0614406	0.901445227	AT5G38200	Class I glutamine amidotransferase-like superfamily protein [Acc:F4KA45]	0														
AT4G33010	-0.240483029	-0.292111991	-0.298268683	-1.184656394	-1.081997013	-0.816188063	GLDP1	Glycine cleavage system P protein [Acc:A0A178UTF1]	0	■													
AT5G67100	-0.380792473	-0.560504471	-0.187342045	-1.225598594	-1.140188307	-0.885442125	POLA	DNA polymerase alpha catalytic subunit [Acc:Q9FHA3]	0											■			
AT2G31980	1.62000037	0.720405817	0.63244232	1.464229966	2.724332952	2.012700545	CYS2	Cysteine proteinase inhibitor [Acc:A0A178VSQ8]	1														
AT1G20190	0.241423721	1.138888281	-0.095620029	1.254784821	1.415455827	1.68423352	EXPA11	Expansin-A11 [Acc:Q9LNU3]	1	■										■			
AT5G66590	0.254733637	1.210418642	-0.43274943	1.119583626	2.109157342	1.588684612	AT5G66590	CAP (Cysteine-rich secretory proteins, Antigen 5, and Pathogenesis-related 1	1														
AT5G05965	-0.902359121	0.379212204	-0.798626044	3.176986777	3.031454649	2.997493715	AT5G05965	Cell wall RBR3-like protein [Acc:Q5XV49]	1														
AT4G35720	-0.889460795	-1.311481953	-1.235757922	2.962895794	2.795141999	1.6015341	AT4G35720	DUF241 domain protein, putative (DUF241) [Acc:O81803]	1														
AT2G25460	0.596139559	0.15029815	0.256973819	2.680262799	2.577521454	2.930031245	AT2G25460	CONTAINS InterPro DOMAIN/s: C2 calcium-dependent membrane targeting	1														
AT5G02580	0.198871759	0.079938367	-0.608690788	2.666193455	3.705084207	3.258933694	AT5G02580	Argininosuccinate lyase [Acc:Q84TG0]	1														
AT3G62090	0.148987654	-0.52744474	0.410510143	2.52584143	2.876967245	2.669902483	PIF6	PIL2 [Acc:A0A178VFI3]	1										■				
AT1G10560	0.054446756	0.098439632	-0.574774837	2.277926	1.823748809	1.814570783	PUB18	U-box domain-containing protein 18 [Acc:Q9XIJ5]	1														■
AT1G11210	-0.449432256	-0.934570455	-0.533174457	2.223425262	1.895467668	1.679417512	AT1G11210	At1g11210/T28P6_5 [Acc:Q9SXA7]	1														
AT5G02540	-0.498390664	-0.452682364	-0.322540949	2.216194132	1.42234917	1.845835084	AT5G02540	NAD(P)-binding Rossmann-fold superfamily protein [Acc:F4KCF2]	1	■													
AT1G06080	-0.613161118	0.162653804	-0.497860641	2.197618972	1.170522016	2.053603222	ADS1	Delta-9 acyl-lipid desaturase 1 [Acc:O65797]	1	■									■				
AT4G16780	0.139095442	0.189739215	-0.201815005	1.992046436	2.61567966	2.264667396	HAT4	Homeobox-leucine zipper protein HAT4 [Acc:Q05466]	1											■			
AT1G07985	0.481766492	-0.037413162	-1.050115443	1.973832363	1.656565266	1.791450342	AT1G07985	Expressed protein [Acc:Q3EDG6]	1														
AT4G23493	0.491516379	0.85267269	0.502791564	1.80172689	2.685600629	1.938498219	AT4G23493	Putative uncharacterized protein [Acc:Q8LC94]	1	■			■										
AT1G02340	0.973973309	0.53253802	0.895693858	1.798788666	2.447514079	2.207254303	HFR1	Transcription factor HFR1 [Acc:Q9FE22]	1	■													■
AT1G56660	0.179902786	-0.235897591	-0.724433676	1.635606013	1.676573916	2.404754695	AT1G56660	F25P12.91 protein [Acc:Q9FXB5]	1											■			
AT1G21550	0.056871235	0.967853505	0.098453304	1.620459164	1.907176983	2.204767896	CML44	Probable calcium-binding protein CML44 [Acc:Q9LPK5]	1	■			■										■
AT4G01250	-0.10823056	0.377819176	0.465180246	1.589055562	1.209570038	1.404033554	WRKY22	WRKY transcription factor 22 [Acc:O04609]	1	■													
AT2G28400	0.507458464	0.695363956	-0.24831001	1.583308569	1.771108474	1.482811742	AT2G28400	Uncharacterized protein At2g28400 [Acc:Q9SKN0]	1	■													
AT1G01110	0.244991796	0.267501437	0.210020517	1.553649759	1.118260326	1.502988572	IQD18	IQ-domain 18 [Acc:Q9MAM4]	1														
AT3G62100	-0.14753615	0.016590961	-0.657045093	1.448621931	1.459749862	1.276620963	IAA30	Auxin-responsive protein (Fragment) [Acc:C0SVF9]	1											■			
AT5G25450	0.737209159	0.997788704	0.488048112	1.400307989	1.790571221	1.32389949	QCR7-2	Cytochrome b-c1 complex subunit 7 [Acc:A0A178UDJ6]	1	■			■										
AT1G04180	0.050286584	0.169056361	0.123248451	1.369601984	1.742792719	1.211557015	YUC9	Flavin-containing monooxygenase [Acc:A0A178WB22]	1														
AT4G30410	0.242577281	0.555797197	-0.06461874	1.343533064	1.159265378	1.532766907	IBL1	Transcription factor IBH1-like 1 [Acc:Q9M0B9]	1	■													
AT3G53230	0.611429197	0.482681833	0.30618238	1.332486853	1.943607093	1.750320206	CDC48D	Cell division control protein 48 homolog D [Acc:Q9SCN8]	1	■			■										
AT5G66580	-0.552982861	0.120567573	-0.89714933	1.237949412	1.320686553	1.030662283	AT5G66580	Uncharacterized protein At5g66580 [Acc:Q9FJY2]	1														
AT3G54200	0.064243313	0.537305132	0.035529534	1.232624703	1.380720829	1.349760348	AT3G54200	Late embryogenesis abundant (LEA) hydroxyproline-rich glycoprotein	1														
AT3G48100	0.505185466	0.666110673	0.414450239	1.202533212	1.777807413	1.721275735	ARR5	Response reactor 2 [Acc:Q0WW39]	1														
AT3G10930	-0.672063039	-0.221937356	-0.391448852	1.19862926	1.067185322	1.052456541	AT3G10930	Uncharacterized protein At3g10930 [Acc:Q84TK4]	1	■													
AT4G22670	0.812189318	0.866614383	0.81822591	1.190138813	1.298696012	1.368509096	AtHip1	FAM10 family protein At4g22670 [Acc:Q93YR3]	1	■													
AT5G40540	0.019109237	-0.144449211	-0.127953375	1.173555321	1.020125256	1.214840616	AT5G40540	Protein kinase ATN1 [Acc:Q9FM43]	1														
AT1G29465	-0.048996055	0.828698528	0.286218755	1.15930717	1.343843502	1.101432006	AT1G29465	Putative uncharacterized protein [Acc:Q0WQY3]	1														
AT5G08130	-0.068909975	-0.490319815	0.243633701	1.147734784	1.613893534	1.567771862	BIM1	basic helix-loop-helix (bHLH) DNA-binding superfamily protein [AT5G08130]	1	■													
AT1G02380	-0.459086195	-0.132258534	0.054533737	1.139661379	1.415762535	1.55721523	AT1G02380	T6A9.7 protein [Acc:Q9FZ23]	1											■			
AT1G09575	0.226101852	0.420930502	-0.31062724	1.108543855	1.000837307	1.278790105	AT1G09575	Calcium uniporter protein 6, mitochondrial [Acc:F4I111]	1	■													
AT1G01260	0.083282132	-0.07247962	0.067646404	1.077591148	1.243024845	1.31755008	BHLH13	Transcription factor bHLH13 [Acc:Q9LNJ5]	1														
AT3G57810	0.664408202	0.50465802	0.513083728	1.04552448	1.088864636	1.310289983	AT3G57810	OTU domain-containing protein At3g57810 [Acc:Q8LBZ4]	1	■			■										
AT1G69010	0.018116476	0.149721249	0.010604947	1.044192262	1.327773322	1.126119019	BIM2	Transcription factor BIM2 [Acc:Q9CAA4]	1														
AT5G48900	-0.152936597	0.389674851	-0.566976381	1.025551906	1.343540485	1.114128759	AT5G48900	Probable pectate lyase 20 [Acc:Q93WF1]	1	■										■			
AT5G56980	0.633832921	0.895096621	0.614067268	1.013479802	1.05552489	1.003257762	A70	unknown protein	1														
AT4G39360	1.009544738	1.087390393	0.349824836	2.731590402	3.211481168	2.583048219	AT4G39360	At4g39360 [Acc:Q5Q0B1]	2														
AT1G53830	0.110842069	0.453284759	-0.764145105	0.404216741	2.867091935	1.896297301	PME2	Pectinesterase 2 [Acc:Q42534]	2	■										■			
AT1G04220	0.781642356	-0.059036251	-0.322415929	0.581578561	2.813168551	0.178638223	KCS2	3-ketoacyl-CoA synthase 2 [Acc:Q5XEP9]	2														
AT2G05440	-0.314868651	0.254146681	0.749640311	0.772567231	2.563107312	1.099083209	ATGRP9	Glycine-rich protein 9 [Acc:Q9SL23]	2														■
AT2G18969	0.425156677	0.933919641	-0.165715124	1.946022559	2.536161288	2.138235705	AT2G18969	A.t. protein match is: sequence-specific DNA binding transcription factors	2														
AT1G72200	0.698110084	0.591174441	0.474514152	1.211913644	2.287010664	1.520846168	ATL11	RING-H2 finger protein ATL11 [Acc:Q84W40]	2														
AT1G70850	0.393234786	0.146367429	0.578778775	1.124486757	2.213928588	1.555353893	MLP34	MLP34 [Acc:A0A384KEJ8]	2														
AT2G20750	0.35060594	0.224364938	-0.483734577	0.450200175	1.925024129	0.736218363	EXPB1	Expansin-B1 [Acc:Q9SKU2]	2										■	■			
AT1G19960	0.388352333	0.827999703	0.447429115	-0.180734169	1.880047148	0.862687955	AT1G19960	Putative uncharacterized protein At1g19960 [Acc:Q9LNS5]	2											■			
AT4G31380	-0.187721257	-0.436875838	-1.319487793	1.770673946	1.876266994	1.483739895	FLP1	Flowering-promoting factor 1-like protein 1 [Acc:Q5Q0B3]	2														
AT1G56170	0.842539206	1.182186417	0.466435223	1.039332053	1.85048586	1.509143428	NFYC2	Nuclear transcription factor Y subunit C-2 [Acc:Q8LCG7]	2	■			■						■				
AT3G17580	0.275910849	0.858871719	-0.365121369	0.947133224	1.847157791	0.4249371	AT3G17580	SsrA-binding protein [Acc:Q9LUN9]	2														
AT1G52827	1.955254997	1.346119356	0.851185245	0.543841932	1.765584897	0.618503011	CDT1	At1g52827 [Acc:Q3ECR7]	2														
AT1G52565	0.004021136	-0.090038835	-0.162809817	0.928481148	1.718339358	0.768334623	AT1G52565	At1g52565 [Acc:A0JPU2]	2														
AT1G64200	0.910068623	0.93101717	0.632425233	0.663496189	1.71305378	0.966455368	VHA-E3	vacuolar H+-ATPase subunit E isoform 3 [AT1G64200]	2	■			■						■				
AT2G42395	-0.469791963	0.162603994	-0.529740767	0.153469554	1.684199479	-0.060165062	AT2G42395	At2g42395 [Acc:Q84TF7]	2														
AT4G23450	0.562605684	0.453216357	0.935031064	0.384847287	1.672811334	1.468686802	AIRP1	RING/U-box superfamily protein [Acc:F4JNK0]	2														
AT3G07150	0.513686948	0.675787869	0.177373901	1.184974088	1.645899267	1.50954776	AT3G07150	Amino acid-ligase [Acc:Q9SFU5]	2	■			■										
AT4G14130	0.590973837	0.627526025	0.302148047	0.746666401	1.64462859	1.01080908	XTH15	Xyloglucan endotransglucosylase/hydrolase protein 15 [Acc:Q38911]	2	■									■	■			
AT5G37670	0.466322406	0.512716731	0.038838783	0.783505921	1.614286525	0.685584596	HSP15.7	15.7 kDa heat shock protein, peroxisomal [Acc:Q9FHQ3]	2	■			■										
AT3G23930	-0.143143856	0.02295391	0.722348011	0.787302636	1.576144865	0.545871748	AT3G23930	Troponin T, skeletal protein [Acc:Q9LIR5]	2														
AT2G31945	0.594918629	0.627488053	0.79306127	1.217446224	1.544205276	0.942277706	AT2G31945	At2g31940/F20M17.2 [Acc:Q8RU85]	2	■				■									
AT3G29810	0.575762181	0.714285687	0.846116235	0.915617738	1.521001719	1.010329938	COBL3	COBRA-like protein 2 [Acc:Q8L8Q7]	2	■			■							■			
AT1G77885	-0.800310022	-0.118731015	-0.893865843	0.806181176	1.517639648	0.655443227	AT1G77885	Putative uncharacterized protein [Acc:Q3E7C8]	2														
AT3G05890	0.133872429	0.55097591	-0.159356852	1.106069403	1.492770253	1.459113262	RCI2B	At3g05890 [Acc:Q67Z65]	2														
AT1G31835	0.440052516	0.876050377	-0.044861967	1.048814993	1.475480974	0.665587348	AT1G31835	unknown protein	2														
AT1G30070	0.502734397	0.633077269	-0.207276522	0.863116702	1.448702184	0.944632636	AT1G30070	SGS domain-containing protein [Acc:F4I4Q9]	2	■			■										
AT1G01120	0.653450369	0.188871319	-0.13313583	0.61405883	1.436616971	0.671407566	KCS1	3-ketoacyl-CoA synthase [Acc:A0A178W124]	2										■				
AT5G40460	0.337055661	0.500397342	0.083075589	0.682916236	1.425020264	0.161577109	SMR6	Cyclin-dependent protein kinase inhibitor SMR6 [Acc:Q29Q81]	2	■													
AT1G20070	0.452045581	0.620819965	0.087113025	0.838046694	1.41658552	0.276449001	AT1G20070	T20H2.15 protein [Acc:Q9LNT3]	2														
AT1G58590	1.141624222	1.343583597	1.153662526	0.130820538	1.387620182	0.348258381	AT1G58590	other RNA [AT1G58590]	2														
AT3G29035	0.462087859	-0.2283887	0.220808938	0.181961541	1.387403103	1.109533121	NAC59	NAC domain-containing protein 59 [Acc:Q9LJW3]	2														
AT1G54120	0.83287522	1.062670715	0.15787471	0.419697922	1.353823428	1.141030582	AT1G54120	F15I1.22 [Acc:Q9SYH0]	2	■													■
AT4G23885	0.087984978	0.812906874	-0.205947428	0.730732433	1.339835281	0.768435845	AT4G23885	At4g23885 [Acc:Q2HIQ5]	2	■			■										
AT1G67050	-0.05578674	0.250544218	-0.246514653	0.526919758	1.324180483	0.875330363	AT1G67050	F1O19.11 protein [Acc:Q9FZH6]	2														
AT5G65390	0.065411942	0.290054009	0.112653059	0.627854623	1.30620263	0.685243872	AGP7	Classical arabinogalactan protein 7 [Acc:Q8LG54]	2	■									■	■			
AT4G23670	0.552210302	0.487603679	0.839294508	0.037387653	1.300311109	0.393646886	AT4G23670	AT4G23670 protein [Acc:Q9SUR0]	2														
AT5G57150	0.180314694	-0.400497584	0.581042858	0.63727389	1.298639789	0.728457131	AT5G57150	Basic helix-loop-helix (BHLH) DNA-binding superfamily [Acc:F4KAJ5]	2														
AT3G04010	0.506103581	0.887365014	0.47281563	0.887807748	1.298393325	0.41421309	AT3G04010	At3g04010 [Acc:Q9SQR1]	2	■										■			
AT3G06420	0.659758087	0.478835214	0.276393666	0.541732353	1.283888491	0.761115284	ATG8H	Autophagy-related protein 8h [Acc:Q8S925]	2	■													
AT2G30360	0.308424805	0.235075496	-0.120486334	0.562610498	1.272909429	0.482280256	CIPK11	CBL-interacting serine/threonine-protein kinase 11 [Acc:O22932]	2	■													
AT4G34760	-0.268168986	-0.199303286	-0.669020762	0.31110442	1.269651053	0.800261643	SAUR50	Auxin-responsive protein SAUR50 [Acc:O65695]	2	■													
AT2G41150	0.497540214	0.472619509	0.105046785	0.975681197	1.268753055	0.778768549	AT2G41150	unknown protein	2														
AT5G01740	0.385459578	0.995526326	0.32229651	0.929656358	1.254959424	0.475142233	AT5G01740	AT5g01740/T20L15_10 [Acc:Q9LZX2]	2	■			■										
AT5G13930	-0.101750735	-0.137399429	-0.357853019	-0.327128798	1.251331267	0.462517331	CHS	Chalcone synthase family protein [Acc:Q460R0]	2	■		■							■				
AT4G21215	0.150698596	0.163635364	0.287889803	0.496990398	1.251241544	0.979879101	AT4G21215	AT4G21215 protein [Acc:B9DFM7]	2														
AT2G23340	0.615525028	0.667622889	0.793147893	0.663563686	1.238927246	0.647372855	ERF008	Ethylene-responsive transcription factor ERF008 [Acc:O22174]	2	■													
AT5G20110	0.087776651	0.373871216	0.022181631	0.647340897	1.22866818	0.81076683	AT5G20110	At5g20110 [Acc:Q6NM36]	2	■													
AT3G17185	0.007704169	0.211870844	0.305091764	0.434908569	1.206268887	0.445406948	TASIR-ARF	TAS3/TASIR-ARF (TRANS-ACTING SIRNA3); other RNA [AT3G17185]	2														
AT4G25260	-0.225674222	0.311619669	-0.756492506	0.832499597	1.201899798	0.810929861	PMEI7	Pectinesterase inhibitor 7 [Acc:Q9SB37]	2	■			■						■				
AT5G66170	0.60628071	0.400956346	-0.097570927	0.567203836	1.185855659	0.54947073	STR18	Sulfurtransferase 18 [Acc:F4JZ39]	2														■
AT4G12080	0.275574001	0.117004572	0.620044407	0.336419014	1.17135977	0.327769766	AHL1	ATAHL1 [Acc:A0A178V7H8]	2														
AT3G14560	0.565039938	0.849990023	-0.245186741	0.533455354	1.166841402	0.458397444	AT3G14560	AT3g14560/MIE1_6 [Acc:Q9LUD8]	2														
AT1G52342	0.846128775	0.601770605	0.4189719	0.325922657	1.156053836	0.521481012	AT1G52342	Putative uncharacterized protein [Acc:Q1G3Y9]	2											■			

AT1G60610	0.327992478	0.354028884	0.597618742	0.346873289	1.148032086	0.586157692	AT1G60610	At1g60610 [Acc:Q4TU35]	2
AT4G30450	0.047026007	0.686096037	-0.974141384	0.025536831	1.146857457	-0.158736571	AT4G30450	At4g30450 [Acc:Q9M0B5]	2
AT1G01470	0.384744476	0.438659875	0.218200433	0.425767776	1.127745649	0.858610797	LEA14	LSR3 [Acc:A0A178W160]	2
AT5G03670	0.000558507	-0.012973307	-0.214724074	0.631916901	1.127432544	0.408086023	AT5G03670	TRM28 [Acc:A0A178UNJ0]	2
AT4G22190	0.134545777	0.289718274	0.173207502	0.861838278	1.116823082	0.995661198	AT4G22190	Serine/arginine repetitive matrix-like protein [Acc:Q8L4C5]	2
AT3G49780	0.100485647	0.28265976	0.146675268	0.064770897	1.108473827	0.543238964	PSK3	Phytosulfokines 3 [Acc:Q9M2Y0]	2
AT2G45400	-0.077972238	0.266134872	-0.578395626	0.690772266	1.107610128	0.618911402	BEN1	Protein BRI1-5 ENHANCED 1 [Acc:O22133]	2
AT4G03320	0.674666772	0.858384825	0.387858122	0.715225719	1.104542898	0.981543034	TIC20-IV	Protein TIC 20-IV, chloroplastic [Acc:Q9ZQZ9]	2
AT3G06868	-0.384133522	0.384033457	-0.018114285	0.285551703	1.103478111	0.424651647	AT3G06868	Vitellogenin-like protein [Acc:Q0WM46]	2
AT1G72470	0.113731782	0.468215963	-0.460886112	0.565613651	1.094205452	0.57087747	ATEXO70D1	Exocyst subunit Exo70 family protein [Acc:Q9C9E5]	2
AT2G22970	-0.075995054	-0.544009939	-0.272880475	0.329015172	1.092692631	0.800452092	SCPL11	Serine carboxypeptidase-like 11 [Acc:A8MQP0]	2
AT1G11545	0.28660896	0.618679095	0.231329604	0.586168178	1.09152783	0.800578021	XTH8	Xyloglucan endotransglucosylase/hydrolase [Acc:A0A178W0W2]	2
AT2G43060	0.521999145	0.841618295	0.208856323	0.571518904	1.08709502	0.654223301	IBH1	Transcription factor IBH1 [Acc:Q9SKX1]	2
AT4G23870	-0.201477596	0.207292281	-0.771398244	0.60923561	1.076545888	0.849702275	AT4G23870	Uncharacterized protein At4g23870; T32A16.40 [Acc:Q9T0A2]	2
AT1G06850	-0.138696897	-0.509195381	0.24951335	0.359685305	1.075705927	0.773203932	AtbZIP52	AtbZIP transcription factor [Acc:Q8VWG0]	2
AT5G60840	-0.477885569	-0.257766185	-0.160562955	0.510783878	1.06437416	0.75487334	AT5G60840	Uncharacterized protein At5g60840/mae1_90 [Acc:Q8GXK2]	2
AT3G49940	0.01673456	-0.200165213	-0.408222884	0.942168299	1.060074579	0.592118069	LBD38	LOB domain-containing protein 38 [Acc:Q9SN23]	2
AT5G51390	0.849449593	0.580004415	0.320258395	0.781337546	1.059775881	0.951357152	AT5G51390	unknown protein; Ha. [AT5G51390]	2
AT2G46310	-0.174205811	0.523403009	0.322616646	0.876898636	1.04698366	0.64220084	CRF5	Ethylene-responsive transcription factor CRF5 [Acc:O82339]	2
AT4G16670	0.657460102	0.578536899	0.689050987	0.902870039	1.043507264	0.486295662	AT4G16670	At4g16670 [Acc:Q5HZ31]	2
AT3G07340	-0.215380202	-0.173554703	0.084865407	0.662958231	1.040431432	0.666375269	BHLH62	Transcription factor bHLH62 [Acc:Q9SRT2]	2
AT2G40610	-0.346076992	0.556234893	-0.394994448	-0.288699181	1.038122497	0.515978963	EXPA8	Expansin-A8 [Acc:O22874]	2
AT2G14900	0.209442198	0.293920377	-0.543721111	0.584379322	1.030194356	0.632214415	GASA7	Gibberellin-regulated protein 7 [Acc:O82328]	2
AT5G21280	-0.005099702	0.212949974	0.283693912	0.55788435	1.026881858	0.320148096	AT5G21280	At5g21280 [Acc:Q9C589]	2
AT3G43270	0.318415114	0.382025407	0.585618372	0.270416586	1.022524039	0.777688455	PME32	Probable pectinesterase/pectinesterase inhibitor 32 [Acc:Q9LXK7]	2
AT4G10040	0.418002158	0.846511015	0.217923428	0.534150319	1.022236011	0.233759899	CYTC-2	CYTC-2 [Acc:A0A178UZN3]	2
AT3G54950	0.415873635	0.271989669	0.170692653	0.82876491	1.013967243	0.861628292	PLP7	Patatin-like protein 7 [Acc:Q9SV43]	2
AT1G04770	-0.13757429	0.303458532	-0.058169292	-0.066240227	1.011081914	-0.116179847	AT1G04770	Protein SULFUR DEFICIENCY-INDUCED 2 [Acc:Q8L730]	2
AT1G52080	0.394193459	0.150972341	0.057300343	0.329932298	1.007294039	0.557777698	AR791	Actin binding protein family [Acc:Q9SAU9]	2
AT5G43950	-0.38423892	0.134120707	0.461465408	0.559049494	1.00591997	0.455066219	AT5G43950	Vacuolar protein sorting-associated protein (DUF946) [Acc:Q9FND1]	2
AT5G60890	-0.444701967	-0.062603115	0.336205148	0.664961842	1.005793934	0.175573275	MYB34	Transcription factor MYB34 [Acc:O64399]	2
AT4G08230	0.980439758	0.917037969	0.375081187	0.644212903	1.00341477	0.94518509	AT4G08230	Glycine-rich protein [Acc:Q8VY68]	2
AT1G28570	-0.085302386	-0.195008447	1.014598027	-0.737164092	-0.849662552	-1.040485786	AT1G28570	GDSL esterase/lipase At1g28570 [Acc:Q9FXJ1]	3
AT4G15248	0.292291202	0.885379224	-0.58153324	-2.84436808	-3.976924119	-6.250191803	MIP1A	B-box domain protein 30 [Acc:Q1G3I2]	3
AT3G53750	-0.083888157	0.139693617	-0.0084116	-0.875526084	-1.177775875	-1.005068325	ACT1	Actin-3 [Acc:P0CJ47]	3
AT2G23600	-0.438163675	-0.401617501	0.155625332	-0.788034456	-0.597600328	-1.012736744	ACL	acetone-cyanohydrin lyase [AT2G23600]	3
AT5G65360	-0.293253034	0.169079029	-0.506382071	-0.722890352	1.323344439	-1.013639568	HTR2	Histone H3 [Acc:Q0WRA9]	3
AT2G30860	-0.419289695	-0.215117818	-0.465142887	-0.753851957	-3.424383137	-1.014385944	GSTF9	GSTF9 [Acc:A0A178VP05]	3
AT4G21910	-0.776737754	-0.736066382	-0.867592392	-0.861586055	-0.511305276	-1.015088412	AT4G21910	MATE efflux family protein [AT4G21910]	3
AT5G19250	0.089158647	0.478884093	-0.219842906	-0.61436701	-0.187316677	-1.024154693	AT5G19250	Uncharacterized GPI-anchored protein At5g19250 [Acc:P59833]	3
AT2G40205	0.059272803	0.585787548	-0.986122766	-0.177604191	-0.66037702	-1.029320395	RPL41G	60S ribosomal protein L41 [Acc:P62120]	3
AT4G32920	-0.275654851	-0.467805102	-0.280681583	-0.796844965	0.197339266	-1.029726341	AT4G32920	Glycine-rich protein [Acc:F4JV81]	3
AT4G22690	-0.90951022	-0.516430903	-0.468462529	-0.421760962	0.267974559	-1.030181687	CYP706A1	Cytochrome P450, family 706, subfamily A, polypeptide 1 [Acc:F4JLY4]	3
AT4G22380	-0.257200288	0.427369253	-0.757751407	-0.31413716	0.323066635	-1.031717014	AT4G22380	At4g22380 [Acc:Q8LCC7]	3
AT5G38430	1.023845184	-1.910844327	-2.201540028	-0.888445544	-0.297152797	-1.034525984	RBCS-1B	Ribulose bisphosphate carboxylase small chain 1B, chloroplastic [Acc:P10796	3
AT1G14170	0.209777518	-0.034224333	-0.644669396	-0.373840418	-0.860046047	-1.041111055	AT1G14170	F7A19.25 protein [Acc:Q9XI71]	3
AT5G33290	-0.377100525	0.019835807	-0.477540025	-0.562630641	-0.174357461	-1.042237163	XGD1	Glycosyltransferase (Fragment) [Acc:W8PUC9]	3
AT5G10400	-0.333609374	0.326940126	-0.790046205	-0.766874407	-0.188906022	-1.042465517	HTR2	Histone H3 [Acc:Q0WRA9]	3
AT1G27130	-0.318622478	0.469176066	-0.024881942	0.00142071	0.872197418	-1.043465122	GSTU13	Glutathione S-transferase U13 [Acc:Q9FUS6]	3
AT2G30870	-0.170449059	0.237739316	-0.592510494	-0.496666147	-0.773226709	-1.046998611	GSTF10	Glutathione S-transferase F10 [Acc:P42761]	3
AT1G09200	-0.242451383	0.403398116	-0.455487912	-0.617739424	0.870219355	-1.047203703	HTR2	Histone H3 [Acc:Q0WRA9]	3
AT3G01120	-0.532596878	-0.366726761	-0.534574149	-0.952038754	-0.556980931	-1.061820818	CGS1	MTO1 [Acc:A0A178VB36]	3
AT5G66750	-0.33463777	-0.283691344	-0.147664106	-0.927385574	-0.126434851	-1.062536982	DDM1	ATP-dependent DNA helicase DDM1 [Acc:Q9XFH4]	3
AT3G24000	-0.339129595	-0.056555141	-0.191977719	-0.438692769	0.527673732	-1.064239307	PCMP-H87	Pentatricopeptide repeat-containing protein mitochondrial [Acc:Q9LIQ7]	3
AT5G52810	0.177856279	0.148214389	-0.284806429	-0.718034392	0.036806722	-1.070441185	SARD4	Protein SAR DEFICIENT 4 [Acc:Q9FLY0]	3
AT5G19151	0.122885754	0.582876937	-0.699125428	-0.229466675	0.770430905	-1.074605781	AT5G19151	unknown protein	3
AT3G62020	0.1167858	0.567376587	-0.199607612	-0.692121341	0.710594685	-1.076154846	GLP10	At3g62020 [Acc:A0JQ09]	3
AT1G78770	-0.41033969	-0.251391936	-0.307602941	-0.730185701	0.595135649	-1.076481217	APC6	Anaphase-promoting complex subunit 6 [Acc:B3DNN5]	3
AT1G08930	-0.224402709	-0.337407091	-0.429322173	-0.316613807	0.812019808	-1.080940066	ERD6	ERD6 [Acc:A0A178W9Q9]	3
AT4G35350	0.140403387	0.348654436	-0.004489975	-0.728632438	-0.879428139	-1.091732496	XCP1	Cysteine protease XCP1 [Acc:O65493]	3
AT3G62150	-0.879833838	-0.311620964	0.143183931	-0.623979155	-0.408300148	-1.097938939	ABCB21	ABC transporter B family member 21 [Acc:Q9M1Q9]	3
AT5G53420	-0.457241651	-0.371478993	-0.464811583	-0.681637152	-0.060431783	-1.099755417	AT5G53420	CCT motif family protein [Acc:Q8L602]	3
AT1G52410	-0.292323651	-0.213664296	0.293957144	-0.968177199	-0.972494183	-1.126113048	TSA1	TSK-associating protein 1 [Acc:F4ICX9]	3
AT1G24020	0.431716525	-0.475599389	-0.23519911	-0.897028024	1.402511198	-1.12952953	MLP423	MLP-like protein 423 [Acc:Q93VR4]	3
AT3G17670	-0.476230612	-0.227453467	0.097564022	-0.620621165	-0.477922838	-1.129677761	AT3G17670	Tetratricopeptide repeat (TPR)-containing protein [Acc:F4J6C9]	3
AT3G54560	0.215999465	0.295699134	-0.417530484	-0.342093088	-0.394688452	-1.132829461	H2AV	Histone H2A [Acc:A0A178V944]	3
AT5G01500	-0.588044316	-0.482790801	-0.93921421	-0.528764718	0.612458355	-1.13447314	TAAC	Thylakoid ADP,ATP carrier protein, chloroplastic [Acc:Q9M024]	3
AT1G73177	-0.087431554	0.322803684	-0.835197714	-0.017217979	0.770920684	-1.143978612	APC13	Anaphase-promoting complex subunit 13 [Acc:Q8L981]	3
AT1G09780	-0.460026994	-0.183630295	-0.967182277	-0.901509206	0.881604592	-1.148047906	PGM1	IPGAM1 [Acc:A0A178W6V9]	3
AT5G61590	-0.370600273	-0.421195669	0.240484608	-0.446664231	0.019081149	-1.162018909	ERF107	Ethylene-responsive transcription factor ERF107 [Acc:Q9FKG2]	3
AT5G65683	-0.332668673	-0.254441107	-0.266640374	-0.593443669	1.202400815	-1.172186059	WAVH2	Probable E3 ubiquitin-protein ligase WAVH2 [Acc:Q0WQX9]	3
AT1G66620	-0.32566814	0.150486442	-0.842176372	-1.155961918	0.683149808	-1.218877592	AT1G66620	E3 ubiquitin-protein ligase SINA-like 2 [Acc:Q9C6H3]	3
AT3G07090	-0.216245431	-0.17788283	-0.744885837	-0.778710488	-0.598788118	-1.22129504	AT3G07090	PPPDE putative thiol peptidase family protein [Acc:Q9SFT9]	3
AT5G59870	-0.441487484	-0.113581146	-0.731122416	-0.653195587	-0.018527762	-1.225668845	HTA6	Probable histone H2A.7 [Acc:Q9FJE8]	3
AT4G38620	-0.56013446	-0.108276613	-0.171885353	-0.594378696	-0.284952241	-1.229403676	MYB4	Transcription repressor MYB4 [Acc:Q9SZP1]	3
AT5G23510	-0.656422648	-0.652807229	-0.533311117	-0.663660008	0.127913701	-1.260834307	AT5G23510	unknown protein	3
AT5G48370	-0.265140653	-0.011915394	-0.104528316	-0.189923954	-0.07657661	-1.283982276	AT5G48370	At5g48370 [Acc:Q9LK77]	3
AT3G23810	-0.72575011	-0.680234963	-0.90286379	-0.714925534	-0.699172936	-1.292955294	SAHH2	Adenosylhomocysteinase [Acc:A0A178VEP9]	3
AT5G03380	-0.371733481	-0.625851121	-0.867078449	-0.099920821	0.353944418	-1.29818044	HIPP06	Heavy metal-associated isoprenylated plant protein 6 [Acc:Q9LZF1]	3
AT4G09310	-0.654890608	-0.51097922	-0.737642532	-0.83802834	-0.494038972	-1.302962378	AT4G09310	At4g09200 [Acc:Q8RX25]	3
AT4G09200	-0.65489057	-0.510979202	-0.737642531	-0.838028329	-0.481138094	-1.302962419	AT4G09200	At4g09200 [Acc:Q8RX25]	3
AT1G04020	-0.290358608	-0.393126956	-0.223424021	-0.590907467	0.7480321	-1.310981874	ATBARD1	ROW1 [Acc:A0A384KH31]	3
AT4G27900	-0.38622931	-0.421768805	-0.161426346	-0.691134851	-0.307918104	-1.311676762	AT4G27900	CCT motif family protein [Acc:Q9SUE8]	3
AT3G53650	-0.031617498	0.449283793	-0.919723551	-0.416426691	-0.560419169	-1.329046574	AT3G53650	Histone H2B.8 [Acc:Q9LFF6]	3
AT1G73630	-0.158696136	0.573580711	-0.469948662	-1.049465637	-0.585886547	-1.331694982	CML26	Probable calcium-binding protein CML26 [Acc:Q9C9U8]	3
AT3G52630	-0.253887883	0.320083167	-0.931070246	-0.481130184	-0.66765082	-1.334781659	RPA3A	Replication protein A 14 kDa subunit A [Acc:Q9LXK1]	3
AT2G38940	0.303492941	0.519413038	-0.464672297	-1.299423118	0.7583749	-1.347324613	PHT1-4	PHT1 [Acc:A0A178VRB1]	3
AT2G07707	-0.087145572	1.004255119	-0.86688554	-0.525104457	0.748576648	-1.356031417	YMF19	ATPase subunit 8 [Acc:G1C2R4]	3
AT1G01370	-0.099038539	-0.361691188	-0.872826913	-0.763102279	0.954419356	-1.364710785	HTR12	Histone H3-like centromeric protein HTR12 [Acc:Q8RVQ9]	3
AT4G27560	-1.384888801	-0.855407237	-1.098711319	-0.774075883	-0.409069361	-1.379983634	UGT79B2	UDP-glycosyltransferase 79B2 [Acc:Q9T080]	3
AT5G17700	-0.620257887	-0.711555144	0.221508084	-0.638137746	-0.229005769	-1.389299079	DTX25	Protein DETOXIFICATION [Acc:A0A178UJQ3]	3
AT3G48490	-0.066287158	0.196773233	-0.798282677	-0.644827867	-0.522507424	-1.414923998	AT3G48490	At3g48490 [Acc:Q8LD28]	3
AT2G38310	-0.434281062	0.022473695	-0.071339634	-0.880804728	-0.541622359	-1.546324878	PYL4	Abscisic acid receptor PYL4 [Acc:O80920]	3
AT4G21870	-1.559687861	-1.362542341	-0.551327765	-0.981553173	-0.309987756	-1.6177064	HSP15.4	15.4 kDa class V heat shock protein [Acc:O49710]	3
AT1G76680	-0.685861302	0.089613473	-0.608795925	-0.574047121	-0.839133398	-1.680553204	OPR1	12-oxophytodienoate reductase 1 [Acc:F4I403]	3
AT1G10380	-0.078851888	0.25076661	-0.338210329	-1.259487704	-0.943156843	-1.697555592	AT1G10380	At1g10380/F14N23_32 [Acc:Q93ZB0]	3
AT2G20560	-0.173848819	-0.175442019	-0.797591535	-0.782450505	0.800629666	-1.708632291	AT2G20560	At2g20560/T13C7.15 [Acc:Q9SIL3]	3
AT4G19810	-0.280599875	0.577109154	-0.342449094	-0.856928175	0.522229142	-1.732794222	ChiC	Class V chitinase [Acc:O81862]	3
AT5G07580	-0.621345558	0.095710075	0.395639361	-0.675261264	0.420993525	-1.737748616	ERF106	Ethylene-responsive transcription factor ERF106 [Acc:Q9LY05]	3
AT4G20325	-0.30806373	0.122294495	-0.759137308	-0.380147849	0.837704913	-1.753795174	AT4G20325	Ribonuclease H2 subunit B [Acc:A8MRG8]	3
AT4G20780	-0.142576333	-0.018233509	-0.49643002	-0.678009338	0.454428962	-1.769312988	CML42	Calcium-binding protein CML42 [Acc:Q9SVG9]	3
AT3G10520	-0.015640512	0.315535785	-0.859756756	-0.731542847	0.60397396	-1.773772958	AHB2	Non-symbiotic hemoglobin 2 [Acc:O24521]	3
AT5G03210	1.104221311	0.799900532	-1.068481113	-1.197147646	0.222012114	-1.794686551	AT5G03210	A. t. genomic DNA, chromosome 5, P1 clone:MOK16 [Acc:Q9FYM8]	3
AT1G21110	0.09306457	-0.176442693	-0.720220536	-0.005113953	-0.959776739	-1.838337698	IGMT3	Indole glucosinolate O-methyltransferase 3 [Acc:Q9LPU6]	3
AT1G78000	0.008377999	-0.350468708	-0.112581813	-0.524996625	0.791862706	-1.86233439	SULTR1;2	Sulfate transporter 1.2 [Acc:Q9MAX3]	3
AT3G09020	-0.246302894	0.143792493	-0.570697376	-0.701082895	-0.557460631	-1.866961853	AT3G09020	Alpha 1,4-glycosyltransferase family protein [Acc:Q9S790]	3
AT4G16265	-0.573722575	0.015405962	-0.535342672	-1.095302274	0.544701093	-1.902183986	NRPB9B	RNA polymerases M/15 Kd subunit [AT4G16265]	3
AT3G63380	-0.927244664	-0.006583726	-1.021837135	-1.207243939	0.447715506	-2.041690958	ACA12	Calcium-transporting ATPase [Acc:A0A178VEV7]	3
AT3G26395	0.327155077	0.178497407	-0.316455132	-0.343074951	-0.916502055	-2.109690275	AT3G26395	None	3
AT5G23750	-0.107949345	-0.355557866	-0.465329805	-1.647380782	-0.242756537	-2.150727853	AT5G23750	Remorin family protein [AT5G23750]	3
AT2G02990	-0.46678258	-0.01988051	-1.360864321	-2.033442612	0.547090784	-2.409401144	RNS1	Ribonuclease 1 [Acc:P42813]	3
AT1G54790	-0.479615565	-0.278116144	-0.578660481	-0.749775586	1.157571796	-2.452109967	AT1G54790	GDSL esterase/lipase At1g54790 [Acc:Q3ECP6]	3
AT5G48920	-0.407385258	-0.325559047	-0.588128571	-0.878905977	0.057969549	-2.455164786	TED7	At5g48920 [Acc:Q9FI79]	3
AT3G10040	-0.530294916	-0.448804687	-0.17615947	-1.616212411	0.675065889	-2.539238044	AT3G10040	Sequence-specific DNA binding transcription factor [Acc:Q8RWY5]	3
AT1G31200	-0.476747901	-0.05795064	-0.852917714	-0.474788287	-1.147257497	-2.582149053	ATPP2-A9	phloem protein 2-A9 [AT1G31200]	3
AT1G64170	-1.321391606	-0.700503477	-0.847544829	-2.514697232	0.736001651	-2.640869803	CHX16	Cation/H(+) antiporter 16 [Acc:Q1HDT3]	3
AT4G20230	-0.350069825	-0.216174536	0.769006606	-1.797353044	-0.473571888	-2.828075443	AT4G20230	Terpenoid cyclases/Protein prenyltransferases superfamily [AT4G20230]	3
AT5G20790	0.432593555	1.17069033	-0.075857148	-2.043598199	-0.258353387	-3.218507718	AT5G20790	unknown protein	3
AT4G30650	-0.727415599	-0.082636013	-0.691388304	-1.764988634	0.200703681	-3.408357677	AT4G30650	UPF0057 membrane protein At4g30650 [Acc:Q9M095]	3
AT5G24655	-0.449412805	0.196524792	-1.221240807	-0.698547866	-0.118731754	-3.615150263	LSU4	Protein RESPONSE TO LOW SULFUR 4 [Acc:Q8L8S2]	3
AT1G17860	0.236358632	0.530582393	-0.190462879	-0.104621609	-0.861967375	-4.033614207	KTI2	Kunitz trypsin inhibitor 2 [Acc:Q9LMU2]	3
AT1G18940	0.273955176	-0.386819644	-0.775770603	-0.722993301	-0.954708444	-5.170790351	AT1G18940	F14D16.8 [Acc:Q9LMD4]	3
AT4G02040	-0.748240488	0.406189895	-1.544419262	0.170547248	0.455397657	-8.044693376	AT4G02040	At4g02040 [Acc:O04248]	3
AT2G44260	-0.582235274	-0.568389727	-0.661554503	-1.27540948	-0.714905437	-9.614964824	AT2G44260	Plant protein of unknown function (DUF946) [AT2G44260]	3
AT1G11090	-0.187553351	-0.208104186	0.306820815	0.689653659	0.841239165	-9.615137622	AT1G11090	Alpha/beta-Hydrolases superfamily protein [Acc:O04083]	3
AT1G71000	-0.057791263	0.110754764	-1.493982277	-4.100376513	-0.742092471	-11.08866814	AT1G71000	Chaperone DnaJ-domain superfamily protein [Acc:F4I8D6]	3
AT5G44110	-0.323606913	-0.705924608	-1.40829589	-0.567711703	-0.593181547	-0.764017507	ABCI21	ABC transporter I family member 21 [Acc:Q9XF19]	4
AT4G14640	-0.535947435	-0.099464898	-1.410029687	0	0	0	CML8	Calmodulin-like protein 8 [Acc:O23320]	4
AT5G54165	-0.383693344	-0.192512462	-1.420162975	-0.164852729	0.284200514	-1.354315959	AT5G54165	Avr9/Cf-9 rapidly elicited protein [Acc:Q570N6]	4
AT1G74010	-1.018435832	-0.331337135	-1.432017139	-1.596778291	-0.440406281	-1.060920767	AT1G74010	At1g74010 [Acc:Q9C9C2]	4
AT5G23360	-0.946007713	-0.293911667	-1.433346968	-0.329467652	0.510078784	0.266272905	AT5G23360	GEM-like protein 7 [Acc:Q9FMW5]	4
AT1G79470	-0.73825967	-0.557143311	-1.438447757	-0.237365705	-0.298357984	-0.267756513	IMPDH	Inosine-5'-monophosphate dehydrogenase 1 [Acc:P47996]	4
AT1G53950	-0.342425363	-0.060957299	-1.444143657	0	0	0	AT1G53950	F15I1.4 protein [Acc:Q9SYF2]	4
AT3G45430	-0.343390272	-0.129175497	-1.445823163	-0.160833866	0.321964566	-0.403790063	LECRK15	Probable L-type lectin-domain containing receptor kinase I.5 [Acc:Q9M1G4]	4
AT3G51680	-0.475167479	-0.391369122	-1.447665231	0	0	0	SDR2A	Short-chain dehydrogenase reductase 2a [Acc:Q9SCU0]	4
AT1G54000	-0.776529515	-0.4770762	-1.449894733	-0.252942393	0.724484574	0.65290989	GLL22	GDSL esterase/lipase 22 [Acc:Q1H583]	4
AT4G26790	-0.147009478	-0.943895748	-1.45234246	-0.335582798	0.815491813	-0.916811968	AT4G26790	GDSL esterase/lipase At4g26790 [Acc:Q8VY93]	4
AT2G38750	-0.07510671	-0.187431706	-1.45463846	-0.701084481	0.269158178	-0.887419887	ANN4	Annexin D4 [Acc:Q9ZVJ6]	4
AT4G08780	-1.424170346	-0.613626575	-1.457831696	-1.913067564	-0.578964025	-2.514357466	PER38	Peroxidase 38 [Acc:Q9LDA4]	4
AT1G01480	-1.009952556	-0.913605938	-1.459450571	-1.236263224	0.486808519	-1.107454713	ACS2	1-aminocyclopropane-1-carboxylate synthase 2 [Acc:Q06402]	4
AT4G22080	-0.283264282	-0.743387995	-1.4616659	0	0	0	RHS14	Probable pectate lyase 16 [Acc:O65456]	4
AT3G59068	-0.928790983	0.418123189	-1.462799568	0.029534373	0.240757893	-0.589239522	AT3G59068	Potential natural antisense gene, locus overlaps with AT3G59070	4
AT1G13590	0.249405345	0.061341047	-1.469072267	-0.327512161	0.556516974	-0.269301593	PSK1	Phytosulfokines 1 [Acc:Q9LMY9]	4
AT3G09680	-0.627033774	-0.051563837	-1.470426425	0	0	0	RPS23A	40S ribosomal protein S23-1 [Acc:Q9SF35]	4
AT5G22410	-0.440303113	-0.158779527	-1.471469064	0	0	0	PER60	Peroxidase 60 [Acc:Q9FMR0]	4
AT1G63930	-1.023485054	-0.548919583	-1.473040544	0	0	0	ROH1	At1g63930 [Acc:Q9CAK4]	4
AT1G01453	-0.862903142	0.21422717	-1.479461114	0	0	0	AT1G01453	unknown protein	4
AT2G20562	-0.423049488	0.642781563	-1.479532626	-0.072643698	0.495458515	-0.351464487	AT2G20562	Putative uncharacterized protein [Acc:Q1G309]	4
AT1G66800	-0.232281921	-0.75993372	-1.479734697	0	0	0	AT1G66800	NAD(P)-binding Rossmann-fold superfamily protein [Acc:F4HQ07]	4
AT5G23990	-0.280191976	-0.794847071	-1.491806273	0	0	0	ATFRO5	ferric reduction oxidase 5 [AT5G23990]	4
AT5G10210	-0.731990847	-0.74193279	-1.494034547	0	0	0	AT5G10210	At5g10210 [Acc:Q9LX09]	4
AT2G18450	-0.254817124	-0.490166841	-1.498532662	-1.351218195	-0.945261082	-1.319339106	SDH1-2	Succinate dehydrogenase [ubiquinone] flavoprotein subunit, mitochon	4
AT3G54150	-1.00179793	-0.471404493	-1.500805492	-0.358318354	-0.982826337	-1.459974966	AT3G54150	Embryonic abundant protein-like [Acc:Q9M389]	4
AT4G36880	-0.262669466	-0.258657643	-1.505118891	0.455875043	-0.654033071	-0.898767973	CP1	CP1 [Acc:A0A178UST0]	4
AT1G57590	-0.41021008	-0.218836521	-1.509369283	0	0	0	PAE2	Pectin acetylesterase 2 [Acc:F4I839]	4
AT1G01750	-0.493760838	-0.08574896	-1.51434838	1.012722961	1.196856165	0.751169165	ADF11	actin depolymerizing factor 11 [AT1G01750]	4
AT3G48520	-0.387937092	-0.16946069	-1.516356276	-0.235019462	0.109725306	0.227633106	CYP94B3	CYP94B3 [Acc:A0A178V8H3]	4
AT3G19710	-1.279085655	-0.474710933	-1.518200725	-0.678833089	0.261018213	-0.091286199	BCAT4	Methionine aminotransferase BCAT4 [Acc:Q9LE06]	4
AT4G18510	-0.718639772	-0.656194129	-1.520631171	0	0	0	CLE2	CLE2 [Acc:A0A178UVM8]	4

AT5G57220	-0.895007366	-0.336094035	-1.523414603	0.020210257	-0.001881821	-0.523211173	CYP81F2	Cytochrome P450 81F2 [Acc:Q9LVD6]	4
AT3G16390	-0.6520252	-0.999407464	-1.523894443	0	0	0	NSP3	Nitrile-specifier protein 3 [Acc:O04318]	4
AT3G20557	0.248056293	-0.728987221	-1.524182869	0	0	0	VUP3	Vascular-related unknown protein 3 [Acc:Q56YT7]	4
AT3G11930	-0.745273441	-0.575158198	-1.52724337	-0.190358115	0.436927444	0.034965978	AT3G11930	AT3G11930 protein [Acc:B9DG73]	4
AT3G61930	-0.064486496	-0.524582591	-1.527632934	0	0	0	AT3G61930	Uncharacterized protein At3g61930 [Acc:Q9M272]	4
AT3G27400	-1.270981289	-0.777905352	-1.544013026	-1.680670423	0.911651972	-1.45610226	AT3G27400	Pectate lyase [Acc:A0A1I9LNU6]	4
AT4G08770	-0.915895081	-0.462169412	-1.550861761	-1.31495977	-0.361575161	-0.552313501	PER37	Peroxidase 37 [Acc:Q9LDN9]	4
AT1G18100	-0.447979871	-0.547668617	-1.553649104	0	0	0	MFT	Protein MOTHER of FT and TFL1 [Acc:Q9XFK7]	4
AT5G44550	-0.437931691	-0.766892582	-1.554720881	-0.880408994	0.045295108	-0.807486023	AT5G44550	CASP-like protein 1B1 [Acc:Q9FI10]	4
AT5G02575	-1.352987196	-0.013260718	-1.589974411	-1.651893187	0.177055191	-1.9040093	AT5G02575	None	4
AT2G21100	0.203899445	-0.051822881	-1.59450562	0	0	0	DIR23	Dirigent protein 23 [Acc:Q84TH6]	4
AT2G43870	-0.476254869	-0.467621066	-1.607820597	0.305055222	0.381942582	-0.74016627	AT2G43870	Pectin lyase-like superfamily protein [Acc:O22817]	4
AT5G23810	-0.648580033	-0.91332444	-1.611494165	-0.323434736	-0.156789025	-0.27012934	AAP7	Probable amino acid permease 7 [Acc:Q9FF99]	4
AT4G38410	-0.045713749	-0.293253764	-1.6150655	0	0	0	AT4G38410	At4g38410 [Acc:Q8GY17]	4
AT2G08685	-0.84400105	-0.899335649	-1.616766854	0	0	0	AT2G08685	None	4
AT2G43120	-1.162380696	-0.48330451	-1.622824152	0.159689078	-0.095503167	-0.166005872	AT2G43120	RmlC-like cupins superfamily protein [AT2G43120]	4
AT5G47220	-0.393427816	-0.020604878	-1.624513656	0.225031223	-0.542993325	-0.648065718	ERF2	ERF2 [Acc:A0A178UCP3]	4
AT3G14440	-1.067907258	-0.027907232	-1.639171886	1.116203896	0.759119838	0.559314491	NCED3	9-cis-epoxycarotenoid dioxygenase NCED3, chloroplastic [Acc:Q9LRR7]	4
AT5G54490	-0.578245519	-0.435976893	-1.660972611	0.476650457	-0.011602405	-0.479981182	PBP1	PBP1 [Acc:A0A178URF6]	4
AT1G17180	-0.910393699	0.223524144	-1.666047755	-0.161649178	1.197411051	-0.352867605	GSTU25	Glutathione S-transferase U25 [Acc:Q9SHH7]	4
AT5G39580	-0.582231762	-0.451935308	-1.678660956	-0.001372683	-0.560351199	-0.218022798	PER62	Peroxidase 62 [Acc:Q9FKA4]	4
AT1G55290	-0.783956076	-0.571482682	-1.712819628	0.514640855	-0.444761668	0.22775762	F6'H2	Feruloyl CoA ortho-hydroxylase 2 [Acc:Q9C899]	4
AT4G21840	-0.213772803	-0.330284997	-1.713421937	0	0	0	MSRB8	MSRB8 [Acc:A0A178V0W9]	4
AT5G57530	-0.470207083	-0.480109499	-1.71893632	0	0	0	XTH12	Probable xyloglucan endotransglucosylase/hydrolase 12 [Acc:Q9FKL9]	4
AT1G59725	-0.367103319	-0.672413999	-1.735546876	0	0	0	AT1G59725	DNAJ heat shock family protein [Acc:Q9XIF5]	4
AT5G59240	-0.434984286	-0.299292123	-1.746568289	0.37487347	1.044736471	-0.503211145	RPS8B	40S ribosomal protein S8 [Acc:Q0WSK1]	4
AT1G51620	-0.768475262	-0.74675218	-1.759244233	-0.386198881	-1.219506281	0.005800878	AT1G51620	Protein kinase superfamily protein [Acc:Q9C8H4]	4
AT1G65500	-0.319552744	-0.189384525	-1.769529653	0.345234117	0.468534849	-0.377169499	AT1G65500	F5I14.4 protein [Acc:O04468]	4
AT3G24500	-0.854510013	-0.679064882	-1.771901929	-0.124993335	0.306353927	-0.087151665	MBF1C	MBF1C [Acc:A0A178VDH9]	4
AT1G15385	-0.268757493	-0.173940917	-1.788368797	0	0	0	AT1G15385	Cotton fiber protein [Acc:Q8L7A6]	4
AT5G06090	0.394214009	-0.97989009	-1.789920528	0	0	0	GPAT7	GPAT7 [Acc:A0A178URK3]	4
AT3G62930	-0.550487231	-0.440236848	-1.80370434	0.24602115	0.431338705	0.24313928	GRXS6	Monothiol glutaredoxin-S6 [Acc:Q9LYC8]	4
AT2G37430	-0.898448483	-0.117270738	-1.80458832	0	0	0	ZAT11	ZAT11 [Acc:A0A178VNJ8]	4
AT5G05270	-0.996613226	-0.966598041	-1.812560792	-0.613633035	-0.124939702	-0.857109417	CHI3	Probable chalcone--flavonone isomerase 3 [Acc:Q8VZW3]	4
AT3G09405	-1.912584074	-1.249647204	-1.825553253	-0.275782085	-0.449115756	0.295867387	PAE4	Pectin acetylesterase 4 [Acc:Q9SR23]	4
AT3G03190	-1.160291469	-0.442631239	-1.850334978	-1.381549712	-0.422212714	-0.872951242	GSTF11	Glutathione S-transferase F11 [Acc:Q96324]	4
AT3G07490	-1.294841627	0.266845625	-1.857477996	0	0	0	CML3	CML3 [Acc:A0A178V7U1]	4
AT1G76650	-1.277791566	-1.276944997	-1.862554475	0.371183031	-0.887339697	0.459122696	CML38	CML38 [Acc:A0A178WMC5]	4
AT4G28110	-0.391395695	-1.335837436	-1.878258814	0	0	0	AtMYB41	MYB41 [Acc:A0A178V452]	4
AT5G02780	-1.39344519	-0.795787313	-1.887235487	0	0	0	GSTL1	Glutathione S-transferase L1 [Acc:Q6NLB0]	4
AT5G36130	-0.556028516	-0.644654803	-1.903842212	0	0	0	AT5G36130	None	4
AT5G15022	-1.075200492	-0.314267862	-1.908279812	0	0	0	AT5G15022	Potential natural antisense gene, locus overlaps with AT5G15030	4
AT1G07795	-0.097214832	-0.073603743	-1.91403945	0	0	0	AT1G07795	At1g07795 [Acc:Q29PW8]	4
AT3G04300	-0.636858389	-0.722203548	-1.919134253	0.467756441	1.194524614	-0.152068333	AT3G04300	At3g04300 [Acc:Q9M8Y6]	4
AT4G10510	-1.508189263	-1.040651076	-1.932354976	0	0	0	SBT3.7	Subtilisin-like protease SBT3.7 [Acc:Q9SZY2]	4
AT3G27070	-1.032238437	-0.37277891	-1.938413401	0	0	0	TOM20-1	Translocase outer membrane 20-1 [Acc:F4JEW8]	4
AT4G22217	-0.06825101	-0.053638644	-1.944658833	0	0	0	AT4G22217	Defensin-like protein 100 [Acc:Q3E6U0]	4
AT2G22880	0.488380523	-0.007569585	-1.954497679	-0.237516949	-0.210903794	-1.456592805	AT2G22880	At2g22880 [Acc:O81005]	4
AT1G73965	-1.01263144	-0.146957818	-1.960278745	0	0	0	CLE13	CLAVATA3/ESR (CLE)-related protein 13 [Acc:Q6NMF0]	4
AT3G16150	-0.757496532	-0.999722589	-1.967137425	-0.694151738	-1.011328958	-1.870380752	AT3G16150	ASPGB1 [Acc:A0A384KV24]	4
AT5G07990	-0.982221025	-1.040807845	-1.977350083	0.221087717	0.081338161	0.195040739	CYP75B1	TT7 [Acc:A0A178UNZ9]	4
AT1G14205	-0.226287473	-0.823596212	-2.005999758	0.067373338	0.214656378	0.196464612	AT1G14205	Ribosomal L18p/L5e family protein [AT1G14205]	4
AT4G18170	-0.924618515	-1.046366264	-2.010766865	0.073606744	0.616614869	-0.081707493	WRKY28	WRKY transcription factor [Acc:A0A178V3M3]	4
AT3G22740	-1.599079028	-1.355851329	-2.018625006	-0.695398085	0.515129315	0.166929276	HMT3	Homocysteine S-methyltransferase 3 [Acc:Q8LAX0]	4
AT1G68350	-0.786708913	-1.248298629	-2.038695166	0	0	0	AT1G68350	At1g68350 [Acc:Q9C9H0]	4
AT4G22470	-0.520903236	-1.202700994	-2.047321734	-2.752283253	-1.080861636	-1.033920723	AT4G22470	Protease inhibitor/seed storage/lipid transfer protein (LTP) family	4
AT1G30990	-0.656649861	-1.08354752	-2.053778642	0	0	0	AT1G30990	F17F8.9 [Acc:Q9FYJ0]	4
AT1G08090	-1.495722951	-1.27580903	-2.058151633	0	0	0	NRT2.1	High-affinity nitrate transporter 2.1 [Acc:O82811]	4
AT4G06880	0.223606068	-0.759202866	-2.072211168	0	0	0	AT4G06880	None	4
AT5G11440	-0.510266381	-0.540655974	-2.086823234	0	0	0	CID5	Polyadenylate-binding protein-interacting protein 5 [Acc:Q9LYE5]	4
AT2G43000	-1.604385608	0.137445829	-2.125869767	0.151995865	0.244189471	-0.750409	JUB1	Transcription factor JUNGBRUNNEN 1 [Acc:Q9SK55]	4
AT5G04120	0.531092087	-0.185082405	-2.13073909	0	0	0	IPSP	Metal-independent phosphoserine phosphatase [Acc:F4KI56]	4
AT3G26830	-1.212771584	-0.901301599	-2.14368376	-0.146101544	0.137684111	-1.551229304	CYP71B15	Bifunctional dihydrocamalexate synthase/camalexin synthase [Acc:Q9LW27]	4
AT1G62280	-1.039577657	-0.7282177	-2.161969777	0	0	0	SLAH1	SLAH1 [Acc:A0A178W8P4]	4
AT1G13480	-0.744164935	-0.968795539	-2.168257168	0	0	0	AT1G13480	At1g13480 [Acc:Q56Y29]	4
AT3G14260	-1.000973478	-0.974581931	-2.329865198	0	0	0	AT3G14260	Protein LURP-one-related 11 [Acc:Q9LUM1]	4
AT2G41480	-1.21126361	-0.922386471	-2.334335182	-1.259452814	1.232886183	-0.358646067	PER25	Peroxidase 25 [Acc:O80822]	4
AT5G59990	-0.438297425	-1.231588231	-2.34669028	1.377896424	0.733597836	0.83317892	AT5G59990	CCT motif family protein [Acc:Q9FJD8]	4
AT1G67980	-1.302711348	-1.180794799	-2.408602143	-0.888132547	0.136558529	-3.936783699	CCOAMT	Putative caffeoyl-CoA O-methyltransferase At1g67980 [Acc:Q9C9W3]	4
AT4G11393	-1.124004402	-0.781505783	-2.448659989	0	0	0	AT4G11393	Putative defensin-like protein 202 [Acc:Q2V3J6]	4
AT2G08780	0.249838242	0.710312652	-2.514108699	-0.228684986	-1.908571657	-1.488793648	AT2G08780	None	4
AT4G22214	-0.495624787	-0.3770176	-2.620409691	0	0	0	AT4G22214	Defensin-like protein 99 [Acc:Q7X6T3]	4
AT3G05950	-1.763909909	-0.725933867	-2.668328119	0	0	0	AT3G05950	Germin-like protein subfamily 1 member 7 [Acc:Q9SFF9]	4
AT3G60270	-1.844716006	0.247969321	-2.699636084	0	0	0	AT3G60270	Cupredoxin superfamily protein [Acc:Q9LY37]	4
AT3G47380	-1.165843354	-1.506817726	-2.723292842	-0.254337769	-0.300054014	-0.650993541	PMEI11	Pectinesterase inhibitor 11 [Acc:Q9STY5]	4
AT4G06775	-0.594441172	-1.881976158	-2.74225535	0	0	0	AT4G06775	None	4
AT2G27535	-2.010253026	-2.148080347	-2.75905983	0	0	0	AT2G27535	Ribosomal protein L10A family protein [Acc:Q8VYN3]	4
AT5G56080	-1.726110274	-1.919854863	-2.769360756	0	0	0	NAS2	NAS2 [Acc:A0A178ULG9]	4
AT1G22900	-1.188472673	-1.179090311	-2.828528709	-1.468485744	-1.89440372	-1.311268723	DIR11	Dirigent protein 11 [Acc:Q67YM6]	4
AT1G10585	-1.048153477	-0.483236449	-2.858729436	-1.1104737	-0.19195469	-1.998874489	BHLH167	Transcription factor bHLH167 [Acc:F4I4E1]	4
AT5G22555	-1.23575943	-1.306718927	-2.969250054	0	0	0	AT5G22555	unknown protein; Ha. [AT5G22555]	4
AT2G02010	-2.670365489	-1.759056622	-3.038603488	0.37463245	-0.12980975	-0.305335101	GAD4	glutamate decarboxylase 4 [AT2G02010]	4
AT5G43068	-0.546381347	-0.945592184	-3.05214289	0	0	0	AT5G43068	Transmembrane protein [Acc:A0A1P8BEV7]	4
AT5G61890	-0.417987314	-1.362108302	-3.078778541	-1.154278303	-0.251735481	-0.133100492	ERF114	Ethylene-responsive transcription factor ERF114 [Acc:Q9FH54]	4
AT4G33905	-0.408598264	0.059350174	-3.081622523	0	0	0	AT4G33905	AT4g33900 [Acc:Q9FPH4]	4
AT3G55970	-2.754826292	-2.353135492	-3.120977973	-2.108800551	0.737560443	-1.498168598	JRG21	JRG21 [Acc:A0A178V764]	4
AT4G31970	-2.491996551	-0.476888916	-3.279441651	0	0	0	CYP82C2	Cytochrome P450 82C2 [Acc:O49394]	4
AT2G24195	-0.835773329	-1.951897373	-3.281273994	0	0	0	AT2G24195	Transmembrane protein [Acc:A0A1P8B1G7]	4
AT2G30750	-2.58649388	-0.904165032	-3.43071559	-1.034042286	0.853997214	-1.319764736	CYP71A12	Cytochrome P450 71A12 [Acc:O49340]	4
AT1G69930	-1.83337231	-1.286339225	-3.437538742	-1.017298006	0.453272013	-0.880595917	GSTU11	Glutathione S-transferase U11 [Acc:Q9CAS6]	4
AT1G49570	-0.683303176	-1.372928066	-3.666709304	0	0	0	PER10	Peroxidase [Acc:A0A178WK78]	4
AT5G05340	-0.266231949	-0.350249799	-3.689902397	0	0	0	PER52	Peroxidase 52 [Acc:Q9FLC0]	4
AT1G69920	-1.587861204	-2.472659167	-4.799018331	0	0	0	GSTU12	Glutathione S-transferase U12 [Acc:Q6NMS0]	4
AT5G52390	-1.383047971	-2.066483856	-5.35906874	-1.584074675	4.302166529	-2.137614606	AT5G52390	PAR1 protein [Acc:Q9FHC1]	4
AT3G22840	-1.077401343	-1.064502329	-2.166505266	-0.345903576	0.218387394	-0.572191718	ELIP1	ELIP1 [Acc:A0A178VCA8]	5
AT5G10230	-0.419895216	-1.080492914	-1.826657425	0	0	0	ANNAT7	Annexin D7 [Acc:Q9LX07]	5
AT5G10625	-0.974157218	-1.082044274	-1.167372813	-0.548190982	0.304182963	-0.345154817	FLP2	Flowering-promoting factor 1-like protein 2 [Acc:Q9LXB5]	5
AT1G64390	-1.26760773	-1.103235382	-1.287976754	0.472681781	0.546115241	0.465749679	AtGH9C2	Endoglucanase 6 [Acc:Q42059]	5
AT5G08640	-0.962638247	-1.124624654	-1.571627212	-0.469004552	-0.36803813	-0.522691821	FLS1	Flavonol synthase/flavanone 3-hydroxylase [Acc:Q96330]	5
AT5G49350	-0.467083726	-1.126501607	-1.099111803	0	0	0	AT5G49350	At5g49350 [Acc:Q84Q60]	5
AT1G29270	-0.99282805	-1.173216868	-1.295722168	-0.506205396	0.891046968	0.177292941	AT1G29270	unknown protein	5
AT3G15340	-0.742524755	-1.220985059	-1.154355688	-0.749187436	-0.707700357	-1.424629917	PPI2	PPI2 [Acc:A0A178VC28]	5
AT2G34790	-1.146869865	-1.274232873	-1.631086035	-0.872115948	-0.439550653	-0.848530876	MEE23	Berberine bridge enzyme-like 15 [Acc:O64743]	5
AT1G49430	-0.041260383	-1.303808884	-1.259143947	-0.231794267	-0.171728721	0.033845179	LACS2	Long chain acyl-CoA synthetase 2 [Acc:Q9XIA9]	5
AT2G03830	-0.902176198	-1.310044207	-1.381120345	0	0	0	RGF8	Probable root meristem growth factor 8 [Acc:Q9SI57]	5
AT3G44550	-0.486889486	-1.339567781	-1.349593511	0	0	0	FAR5	Probable fatty acyl-CoA reductase 5 [Acc:Q0WRB0]	5
AT3G04370	-0.260492045	-1.432361702	-1.47287144	0	0	0	CRRSP39	Cysteine-rich repeat secretory protein 39 [Acc:Q6E263]	5
AT1G67110	-1.152080329	-1.503496818	-1.331368655	0	0	0	CYP735A2	Cytokinin hydroxylase [Acc:Q9ZW95]	5
AT5G49620	-1.314999171	-1.55688537	-1.664958955	0	0	0	AtMYB78	myb domain protein 78 [AT5G49620]	5
AT1G59730	-0.603281163	-1.639691294	-2.5289511	0	0	0	TRX7	Thioredoxin [Acc:A0A178WRN3]	5
AT1G55940	-0.718610506	-1.639989671	-1.156153511	0	0	0	CYP708A1	cytochrome P450, family 708, subfamily A, polypeptide 1 [AT1G55940]	5
AT4G35160	-0.848089611	-1.697794263	-1.515288911	0	0	0	ASMT	Acetylserotonin O-methyltransferase [Acc:Q9T003]	5
AT1G30760	-1.859478372	-1.821661452	-2.718060218	-3.364396186	0.401645987	-0.618621465	AT1G30760	FAD-binding Berberine family protein [AT1G30760]	5
AT5G12270	-1.421199301	-1.936774639	-2.143150912	0	0	0	AT5G12270	1-AMINOCYCLOPROPANE-1-CARBOXYLATE OXIDASE-like [Acc:Q94CL5]	5
AT1G01580	-1.355544583	-2.285457849	-3.259856622	-0.491101405	-0.879446019	-0.310280702	FRO2	ferric reduction oxidase 2 [AT1G01580]	5
AT4G19690	-2.242780274	-2.796673981	-3.460337508	0	0	0	IRT1	Fe(2+) transport protein 1 [Acc:Q38856]	5
AT5G19890	-1.861775731	-3.070502216	-2.89855186	0	0	0	PER59	Peroxidase 59 [Acc:Q39034]	5
AT1G64160	-2.159582504	-3.552050714	-5.529939606	0	0	0	DIR5	Dirigent protein 5 [Acc:Q9SH66]	5
AT2G15780	-2.024448835	-3.919219486	-5.570754038	0	0	0	AT2G15780	At2g15780/F19G14.22 [Acc:Q9XIL1]	5
AT3G09440	-1.283623938	-1.21149046	-2.130375621	-2.356645056	-2.30490148	-3.205255705	HSP70-3	Heat shock protein 70 (Hsp 70) family protein [Acc:A0A178VI76]	6
AT5G05410	-1.465562496	-1.607289911	-2.193693063	-1.581852891	-2.408766409	-1.584601687	DREB2A	Dehydration-responsive element-binding protein 2A [Acc:O82132]	6
AT1G72416	-2.143391533	-2.276459521	-2.490559391	-2.232593635	-2.021950994	-3.136726636	AT1G72416	Chaperone DnaJ-domain superfamily protein [Acc:B3H5X6]	6
AT3G14200	-2.210697987	-2.066235381	-2.485750529	-1.627656131	-1.792967001	-2.301223258	AT3G14200	Chaperone DnaJ-domain superfamily protein [Acc:Q9LJG5]	6
AT1G14200	-2.315524458	-2.26604664	-3.297080823	-2.478758302	-2.904281191	-3.275951508	AT1G14200	At1g14200 [Acc:Q9XI67]	6
AT3G51910	-2.931172854	-2.931642232	-2.992279945	-1.803804978	-2.20718564	-2.792734728	HSFA7A	Heat stress transcription factor A-7a [Acc:Q9SV12]	6
AT3G16050	-1.067696815	-0.919366056	-1.57789074	-1.200201863	-1.121214859	-1.630224667	PDX12	Pyridoxal 5'-phosphate synthase-like subunit PDX1.2 [Acc:Q9ZNR6]	6
AT1G66080	-1.180220374	-0.865918555	-1.852267877	-1.093150352	-1.302879156	-2.470367594	AT1G66080	At1g66080 [Acc:Q9C8D7]	6
AT5G62430	-0.978215907	-1.408230774	-2.193500755	-1.46939912	-1.139399613	-1.257107071	CDF1	Cyclic dof factor 1 [Acc:Q8W1E3]	6
AT5G62360	-1.369191664	-1.819533666	-2.466560824	-2.273880239	-2.847196167	-2.195173235	AT5G62360	Plant invertase/pectin methylesterase inhibitor superfamily [Acc:Q9LVA3]	6
AT2G26150	-1.138655235	-1.494756058	-1.382194481	-1.653024437	-1.363534873	-1.746588165	HSFA2	Heat stress transcription factor A-2 [Acc:O80982]	6
AT2G08770	-0.608316748	2.508265698	-2.986554835	-0.973911447	-0.572071149	-2.43834648	AT2G08770	None	6
AT1G21120	-0.819428464	-1.110483916	-2.509645666	-1.826386239	-0.907653213	-1.982756032	AT1G21120	O-methyltransferase family protein [AT1G21120]	6
AT5G04340	-0.859530506	-0.806674797	-1.717021953	-1.527789256	-1.365388108	-2.528730187	ZAT6	Zinc finger protein ZAT6 [Acc:O22533]	6
AT5G56010	-0.988265609	-0.819622108	-2.024050145	-1.5800542	-1.641102422	-2.076834392	HSP90-3	Heat shock protein 90-3 [Acc:P51818]	6
AT2G27402	-0.044420289	-0.69173903	-2.335108025	-1.16774706	-0.943315768	-1.986845008	AT2G27402	At2g27402 [Acc:Q8GX30]	6
AT1G74030	-0.344348057	-0.166027496	-1.018520629	-1.265431773	-0.960418415	-1.248585114	ENO1	Enolase 1, chloroplastic [Acc:Q9C9C4]	6
AT3G17611	-0.803514022	-0.596808233	-1.344059585	-1.382317858	-0.831156866	-1.496164764	RBL14	Rhomboid-like protein 14, mitochondrial [Acc:Q8RXW0]	6
AT1G24575	-1.12875693	-0.38212484	-1.272539906	-1.41449658	0.268843726	-1.406458979	AT1G24575	At1g24575 [Acc:Q8LAL6]	6
AT5G56030	-0.929982262	-0.741100653	-1.522002072	-0.972082747	-1.044235144	-1.065564024	HSP81-2	Heat shock protein 81-2 [Acc:F4K6B6]	6
AT1G20693	-0.975326937	-0.997028542	-1.424105896	-0.709626775	-1.09249771	-1.17555852	HMGB2	High mobility group B protein 2 [Acc:O49596]	6
AT5G65040	-0.336172416	-0.411760416	-1.130167268	-0.547545868	0.010076604	-1.785541013	IRM1	Protein INCREASED RESISTANCE TO MYZUS PERSICAE 1 [Acc:Q9LV75]	6
AT3G28930	-0.571929148	-0.121518671	-1.173094702	-0.554692712	-0.541146203	-1.063368235	AIG2A	Protein AIG2 A [Acc:P54121]	6
AT3G46940	-0.380160137	0.455130573	-1.1888072	-1.072792985	0.38698675	-1.375161244	DUT1	dUTP-PYROPHOSPHATASE-LIKE 1 [Acc:A0A1I9LM85]	6
AT1G01600	0.138295588	0.047050133	-1.266001936	-0.753036063	-0.973494918	-1.750179671	CYP86A4	Cytochrome P450 86A4 [Acc:Q9LMM1]	6
AT3G12965	-0.202021766	0.2276622	-1.282508966	-0.323932734	-0.706519179	-1.09090949	AT3G12965	other RNA [AT3G12965]	6
AT1G26800	-0.789779872	-0.70115867	-1.298157658	-0.810124965	1.013202946	-1.564459392	MPSR1	E3 ubiquitin-protein ligase MPSR1 [Acc:Q9LQX2]	6
AT5G18290	-0.730218397	-0.585263011	-1.298972896	-1.261305582	-0.186105333	-1.802650641	SIP1-2	Probable aquaporin SIP1-2 [Acc:Q9FK43]	6
AT1G79075	0.102312109	0.802443521	-1.379466072	-0.032011911	-3.351208807	-1.125341028	AT1G79075	other RNA [AT1G79075]	6
AT2G16060	-0.893232858	-0.170501808	-1.442934092	-0.263975296	0.799089042	-1.918622877	AHB1	NSHB1 [Acc:A0A384KL50]	6
AT4G22710	-1.71883488	-1.673865374	-1.832908384	-0.275753951	-0.279299785	-1.778274894	CYP706A2	Cytochrome P450 - like protein [Acc:O49652]	6
AT1G02930	-0.869295068	-0.219465067	-2.125704676	-0.710185246	-0.963712738	-1.05428109	GSTF6	Glutathione S-transferase F6 [Acc:P42760]	6
AT2G28210	-1.715206428	-1.593851305	-2.434109234	-0.805685198	-0.661301903	-2.251468438	ATACA2	ATACA2 [Acc:A0A178VW72]	6
AT1G68290	-1.591187789	-1.140805114	-2.771499746	-1.664129645	-1.418886855	-2.97263484	ENDO2	Endonuclease 2 [Acc:Q9C9G4]	6
AT5G53870	1.139593924	1.494486364	2.894370812	-0.127974629	1.468310036	0.214838695	ENODL1	At5g53870 [Acc:Q9FN39]	7
AT1G50960	-0.390534081	-0.100209262	2.761334635	0	0	0	GA2OX7	Gibberellin 2-beta-dioxygenase 7 [Acc:Q9C6I4]	7
AT5G66110	1.93864112	1.778945226	2.549865082	0.577281041	1.001893365	0.440986345	HIPP27	Heavy metal-associated isoprenylated plant protein 27 [Acc:Q67ZW1]	7
AT1G03620	0.696865839	-0.757155491	2.484210972	0.301478913	0.097914596	0.828662163	AT1G03620	ELMO/CED-12 family protein [AT1G03620]	7
AT5G59305	1.990429935	0.645871805	2.459816821	0	0	0	CLE46	CLAVATA3/ESR (CLE)-related protein 46 [Acc:Q8LDN4]	7
AT3G51220	1.106282733	0.410557355	2.439950534	0.15646436	-0.149078505	-1.06783612	AT3G51220	WEB family protein At3g51220 [Acc:Q9SD24]	7

AT3G08115	0.364735112	1.400854415	2.279186195	0	0	0	AT3G08115	None	7
AT5G22860	0.632659711	0.157006422	2.274470987	-0.061023856	0.014534307	-0.362672155	AT5G22860	Prolylcarboxypeptidase-like protein [Acc:Q9FFC2]	7
AT3G54500	1.078821489	-0.112962163	2.244643871	0.116500434	0.278370147	0.868034165	AT3G54500	dentin sialophosphoprotein-related	7
AT2G34420	1.300630061	1.561258192	2.19331617	0.378549538	0.306012242	0.46822374	LHB1B2	Chlorophyll a-b binding protein, chloroplastic [Acc:Q39141]	7
AT5G01190	0.415373403	0.269012891	2.145637471	0.000149375	-0.0043079	-1.116105071	LAC10	Laccase-10 [Acc:Q6ID18]	7
AT3G59060	0.805500864	0.605043732	2.045381088	0.280447388	0.105985218	0.41025658	PIF5	Transcription factor PIF5 [Acc:Q84LH8]	7
AT5G10970	0.706097261	0.858919981	2.020656314	0	0	0	AT5G10970	C2H2 and C2HC zinc fingers superfamily protein [Acc:Q9LEU3]	7
AT5G65590	-0.137000926	0.327988396	2.01575477	0.099059401	-0.222161657	-0.459415297	DOF5.7	Dof zinc finger protein DOF5.7 [Acc:Q9LSL6]	7
AT5G54270	1.270950144	1.000832871	1.991305058	0.001668651	0.348361223	0.295566494	LHCB3	Chlorophyll a-b binding protein 3, chloroplastic [Acc:Q9S7M0]	7
AT2G22990	0.570851258	-0.125484546	1.932212688	0.143127056	0.353384799	0.697039997	SNG1	sinapoylglucose 1 [AT2G22990]	7
AT1G78490	-0.230241036	0.735902084	1.889328367	0.055057512	0.085204374	-0.164250478	CYP708A3	CYP708A3 [Acc:A0A178WMR8]	7
AT5G14920	-0.244733388	-0.031664536	1.874067976	0.185752688	-0.102528164	-0.51764989	GASA14	Gibberellin-regulated protein 14 [Acc:Q9LFR3]	7
AT1G48700	1.872304212	0.999185888	1.854821498	0	0	0	AT1G48700	2-oxoglutarate (2OG) and Fe(II)-dependent oxygenase superfamily	7
AT3G08940	1.343239498	0.914952791	1.822768047	0.066416439	0.139359049	0.085109317	LHCB4.2	Chlorophyll a-b binding protein CP29.2, chloroplastic [Acc:Q9XF88]	7
AT2G15880	0.526643457	-0.558661147	1.766989322	0.621150317	-0.413835161	0.092042336	PEX3	Pollen-specific leucine-rich repeat extensin-like protein 3 [Acc:Q9XIL9]	7
AT5G24770	0.059624056	0.463249664	1.732240192	-1.061131855	0.526784958	-0.727214203	VSP2	Vegetative storage protein 2 [Acc:O82122]	7
AT4G20235	0.227234751	0.002962315	1.72986578	0	0	0	CYP71A28	cytochrome P450, family 71, subfamily A, polypeptide 28 [AT4G20235]	7
AT1G52220	0.921126926	1.005756888	1.707002769	-0.191898246	0.302738794	-0.261682067	CURT1C	CURT1C [Acc:A0A178WBD4]	7
AT1G72645	0.157112149	-0.788379731	1.684162285	0.450296702	0.95385538	0.766086753	AT1G72645	At1g72645 [Acc:Q8RXY2]	7
AT3G23290	0.428962973	0.60781634	1.681500973	-0.13206087	0.436960344	0.143911262	LSH4	LSH4 [Acc:A0A178VHD2]	7
AT4G18020	0.702198818	0.145948233	1.675173108	0.336475813	0.258899891	0.592641	APRR2	Two-component response regulator-like APRR2 [Acc:Q6LA43]	7
AT4G37970	0.207730019	0.689066354	1.659295536	-0.588471978	0.376969665	-2.104918166	CAD6	Probable cinnamyl alcohol dehydrogenase 6 [Acc:O65621]	7
AT1G07440	0.206850162	0.651728469	1.65635593	0.26899459	0.055619656	0.263227271	AT1G07440	Tropinone reductase homolog At1g07440 [Acc:P0DKI3]	7
AT3G56260	0.728228309	-0.069500879	1.653656772	0.382814562	1.061963299	0.164659917	AT3G56260	At3g56260 [Acc:Q6NLT4]	7
AT3G22410	0.653006495	0.520410159	1.642290795	0.340296805	0.255809712	0.15083649	AT3G22410	Sec14p-like phosphatidylinositol transfer family protein [Acc:Q9LUV9]	7
AT2G35260	0.497514693	0.257796448	1.640601343	0.129445906	0.060604617	0.295833037	AT2G35260	At2g35260/T4C15.7 [Acc:O82167]	7
AT1G77090	0.659929122	0.250751027	1.625850796	-0.026791101	-0.211703474	0.105107632	PPD4	PsbP domain-containing protein 4, chloroplastic [Acc:O49292]	7
AT1G61520	1.041493885	0.851633408	1.615083493	0.066626412	0.029580702	0.179509544	LHCA3	Chlorophyll a-b binding protein, chloroplastic [Acc:A0A178W5Y6]	7
AT1G02300	0.453040507	-1.137316526	1.596355315	0.537277342	0.238132921	0.65119492	CATHB1	Cathepsin B-like protease 1 [Acc:F4HVZ1]	7
AT3G54260	-0.020274118	0.029985104	1.592054222	0.11786202	0.398329839	-0.314453378	TBL36	Protein trichome birefringence-like 36 [Acc:Q940H3]	7
AT4G27440	0.247898255	0.502989522	1.573983345	0.126711553	-0.701433732	-0.108493777	PORB	Protochlorophyllide reductase B, chloroplastic [Acc:P21218]	7
AT4G30270	0.671000962	0.501539847	1.557406551	0.273167773	0.224185084	-0.071912773	XTH24	Xyloglucan endotransglucosylase/hydrolase protein 24 [Acc:P24806]	7
AT2G20180	0.859104519	0.281173079	1.546307433	-0.009021578	0.119011302	0.281584713	PIF1	Transcription factor PIF1 [Acc:Q8GZM7]	7
AT4G32810	0.484911766	-0.09384057	1.541746533	-2.553088155	-3.067665356	0.247713219	CCD8	carotenoid cleavage dioxygenase 8 [AT4G32810]	7
AT5G10946	0.441096515	0.255683452	1.540584674	0.464402236	0.839086453	0.671129082	AT5G10946	unknown protein	7
AT1G20620	0.024037899	0.126521567	1.533906673	-0.088200569	-0.205737085	-0.173187861	CAT3	catalase 3 [AT1G20620]	7
AT1G74470	0.731649796	0.496922087	1.529784777	-0.114418794	-0.009195816	0.156186506	CHLP	Geranylgeranyl diphosphate reductase, chloroplastic [Acc:Q9CA67]	7
AT1G22990	1.00171399	0.497747536	1.529059817	0	0	0	HIPP22	HIPP22 [Acc:A0A178WF89]	7
AT2G29650	0.521119241	-0.206700565	1.527736625	-0.296043244	-0.431118341	-0.013045814	ANTR1	Sodium-dependent phosphate transport 1, chloroplastic [Acc:O82390]	7
AT1G06520	-0.105057135	-0.481170646	1.524542767	-0.060948023	0.242037449	0.050201471	GPAT1	Glycerol-3-phosphate acyltransferase 1 [Acc:Q9SHJ5]	7
AT5G44572	0.483372038	0.40641353	1.521190992	-0.168590503	0.500562491	-0.54554159	AT5G44572	Transmembrane protein [Acc:Q0WML3]	7
AT3G17040	0.421685303	-0.093400547	1.517348315	-0.001908296	0.071311051	0.279316811	HCF107	Protein high chlorophyll fluorescent 107 [Acc:Q8RWG2]	7
AT3G50820	1.603625223	0.81155534	1.516460281	0.018618983	0.125999775	0.143427033	PSBO2	Oxygen-evolving enhancer protein 1-2, chloroplastic [Acc:Q9S841]	7
AT1G19720	0.016981549	-0.447542726	1.510887273	-0.000362088	-0.539885587	0.404813984	DYW7	Pentatricopeptide repeat-containing protein At1g19720 [Acc:Q9FXH1]	7
AT3G16520	0.375066214	-0.049271021	1.504255714	-0.045601191	0.240238124	0.172865147	UGT88A1	UDP-glycosyltransferase 88A1 [Acc:Q9LK73]	7
AT2G20260	0.839912734	0.860959061	1.473663995	0.009605613	-0.081952152	-0.108536774	PSAE2	Photosystem I reaction center subunit IV B, chloroplastic [Acc:Q9S714]	7
AT5G23000	0.115474968	0.34139473	1.468369321	0	0	0	RAX1	Transcription factor RAX1 [Acc:Q9FG68]	7
AT3G56940	0.776187592	0.261641948	1.468103276	-0.194025444	-0.013338973	0.102469546	CRD1	Magnesium-protoporphyrin IX monomethyl ester [oxidative] cyclase, chlo	7
AT1G69230	-0.061844579	0.315557516	1.465253223	0.436069124	0.108738273	0.077911503	SP1L2	Protein SPIRAL1-like 2 [Acc:Q9LE54]	7
AT4G35440	-0.036177748	-0.207486314	1.45988653	0.177246546	-0.596845235	0.296172246	CLC-E	Chloride channel protein [Acc:F4JN11]	7
AT5G61290	0.712463965	-0.016127209	1.456518274	-0.630385219	-0.476067609	-0.118252629	AT5G61290	Flavin-containing monooxygenase FMO GS-OX-like 8 [Acc:Q9FLK4]	7
AT5G06510	0.29064011	-0.241106427	1.443136304	0.15019981	-0.082509276	0.16389971	NFYA10	NF-YA10 [Acc:A0A178URE3]	7
AT1G43040	0.392689312	1.273183962	1.44108751	0	0	0	AT1G43040	SAUR-like auxin-responsive protein family [AT1G43040]	7
AT4G33000	0.575146915	0.21515831	1.440217505	-0.276783639	-0.312730809	-0.429178237	CBL10	Calcineurin B-like 10 [Acc:M5BEI3]	7
AT5G01530	0.968328939	0.6806237	1.434944138	-0.154664406	-0.17675139	-0.192886987	LHCB4.1	Chlorophyll a-b binding protein CP29.1, chloroplastic [Acc:Q07473]	7
AT5G19530	0.339238848	0.225235	1.433736102	0.309379113	-0.008125671	0.051078214	ACL5	Thermospermine synthase ACAULIS5 [Acc:Q9S7X6]	7
AT5G54530	0.492463152	-0.186807582	1.425144003	0.719609624	0.111137401	0.146293876	AT5G54530	Uncharacterized protein At5g54530/MRB17_3 [Acc:Q8GYV1]	7
AT5G14370	0.501851906	0.488349949	1.420105407	0.227463508	0.259217052	-0.035049438	AT5G14370	CCT motif family protein [Acc:Q949T9]	7
AT3G56250	0.212897661	-0.46038385	1.416819792	-1.109144021	-0.17562944	0.341686877	AT3G56250	unknown protein; Ha. [AT3G56250]	7
AT3G53960	0.465325395	0.065649715	1.416282824	-0.074368996	-0.187103238	0.096937165	NPF5.7	Protein NRT1/ PTR FAMILY 5.7 [Acc:Q9M331]	7
AT3G10405	0.521627362	0.280265881	1.414470778	0.086196364	0.221942105	0.190610907	AT3G10405	At3g10405 [Acc:A0JQ78]	7
AT4G16410	0.642582513	0.184012749	1.413408563	0.12050185	-0.030274579	-0.082185615	AT4G16410	Transmembrane protein [Acc:Q8VZK4]	7
AT1G06680	0.988995744	0.785742388	1.407591871	-0.137362117	-0.010982316	-0.123183732	PSBP1	Oxygen-evolving enhancer protein 2-1, chloroplastic [Acc:Q42029]	7
AT4G23496	-0.092117926	0.209120191	1.397023247	0	0	0	SP1L5	Protein SPIRAL1-like 5 [Acc:Q8LGD1]	7
AT3G57190	0.586584176	0.460839755	1.390428745	0.235293734	-0.217400402	0.298309383	PRFB3	PrfB3 [Acc:A0A178VAE2]	7
AT5G46110	0.649129668	-0.148269865	1.388980555	-0.402279267	-0.25454879	-0.122885974	APE2	Glucose-6-phosphate/phosphate translocator-like [Acc:A0A219I0W9]	7
AT3G20820	0.462911542	0.67066859	1.38773683	0.452880159	0.696698896	0.55461806	AT3G20820	Leucine-rich repeat (LRR) family protein [Acc:Q9LT39]	7
AT5G19940	0.70527083	0.382812311	1.380971652	-0.040604483	0.190539207	0.199306876	PAP8	Probable plastid-lipid-associated protein 8, chloroplastic [Acc:Q941D3]	7
AT1G15820	1.354770583	0.914757163	1.377910526	0.076374411	0.188591557	0.063381983	LHCB6	Chlorophyll a-b binding protein, chloroplastic [Acc:Q9LMQ2]	7
AT5G18930	0.489115196	0.12322006	1.376231824	0.073207913	0.054801372	-0.127613413	SAMDC4	S-adenosylmethionine decarboxylase proenzyme 4 [Acc:Q3E9D5]	7
AT1G62770	-0.046204127	-0.251072853	1.37447303	0.686568478	-0.063220849	-0.064908647	PMEI9	Pectinesterase inhibitor 9 [Acc:Q9SI72]	7
AT1G14700	1.00507177	0.250942021	1.36982753	0.067804208	0.083374778	0.358855146	PAP3	Purple acid phosphatase 3 [Acc:Q8H129]	7
AT2G01990	0.193635116	-0.058323238	1.36338628	0.386632301	0.175757267	0.831479853	AT2G01990	unknown protein	7
AT1G21350	0.827783261	0.507598164	1.362460713	0.431292627	0.596316854	0.607459585	AT1G21350	Thioredoxin superfamily protein [AT1G21350]	7
AT1G35140	0.042288366	-0.913349757	1.349759158	0.780606763	0.772627043	0.860317119	EXL1	Protein EXORDIUM-like 1 [Acc:Q9C6E4]	7
AT1G25440	0.641907193	0.336462801	1.348322182	-0.315132356	-0.268354259	-0.112432764	COL16	Zinc finger protein CONSTANS-LIKE 16 [Acc:Q8RWD0]	7
AT3G18890	-0.037139645	-0.333507879	1.346397875	-0.299671148	-0.47494614	-0.018033868	TIC62	Protein TIC 62, chloroplastic [Acc:Q8H0U5]	7
AT4G14540	0.792255516	0.933834634	1.344745921	0.062950241	0.33216641	0.283795035	NFYB3	Nuclear transcription factor Y subunit B-3 [Acc:O23310]	7
AT3G13810	0.212451029	-0.065089689	1.342162183	-0.002347144	-0.073332823	-0.362389977	AtIDD11	indeterminate(ID)-domain 11 [AT3G13810]	7
AT4G01050	0.412886406	0.302803877	1.337620591	-0.168423001	-0.119300389	-0.073328314	TROL	thylakoid rhodanese-like [AT4G01050]	7
AT5G49440	0.837222685	0.786421288	1.335971979	0.455993593	0.548130054	0.49653681	AT5G49440	AT5g49440/K7J8_12 [Acc:Q9FGX3]	7
AT1G29240	0.102287737	0.0452041	1.333199959	0.53160065	0.375199387	0.50042561	AT1G29240	F28N24.8 protein [Acc:Q9LP52]	7
AT3G28100	0.506909398	0.144611743	1.313701046	-0.509970671	0.50959112	0.216612908	AT3G28100	WAT1-related protein At3g28100 [Acc:Q9LRS5]	7
AT2G41190	0.987973319	1.035882295	1.302392221	-0.536574713	0.612555283	-0.32586062	AVT1A	Amino acid transporter AVT1A [Acc:O80668]	7
AT4G13280	-0.088801632	-1.089321767	1.3012605	0	0	0	TPS12	(Z)-gamma-bisabolene synthase 1 [Acc:Q9T0J9]	7
AT5G13630	0.776050471	0.344641089	1.296068759	-0.470290964	-0.402495142	-0.117674918	CHLH	Magnesium-chelatase subunit ChlH, chloroplastic [Acc:Q9FNB0]	7
AT4G19420	0.343590165	0.388444987	1.293309322	0.073360196	0.565327826	0.30560645	AT4G19420	Pectinacetylesterase family protein [AT4G19420]	7
AT5G39530	0.400500953	0.022972852	1.292138084	-0.106287483	0.129163373	0.254750554	AT5G39530	Uncharacterized protein At5g39530/MUL8_210 [Acc:Q8GWM5]	7
AT1G60000	0.785235539	0.63929899	1.279184102	0.177873651	0.12697645	-0.080119329	AT1G60000	AT1G60000 protein [Acc:Q9ZUJ3]	7
AT1G67910	0.27941453	0.44534319	1.266345689	0.132282128	-0.102063711	0.161472276	AT1G67910	AT1G67910 protein [Acc:Q9C9V7]	7
AT2G33850	-0.437654256	-0.02563936	1.264441933	-0.290700859	0.409238129	-0.590288075	AT2G33850	E6-like protein [Acc:P93019]	7
AT2G21187	0.971088309	-0.06502993	1.263316959	0.234511996	1.015002449	0.583729557	AT2G21187	other RNA [AT2G21187]	7
AT1G35830	0.179031698	-0.102397366	1.254927989	0.383878213	0.445847396	0.09426057	AT1G35830	VQ motif-containing protein [Acc:Q9C8B9]	7
AT1G19540	0.120216149	-0.679272515	1.254582905	0.679741314	0.147443433	0.062395401	AT1G19540	At1g19540 [Acc:Q29PX7]	7
AT4G37800	-0.343963205	0.057049649	1.249898975	-0.741372235	0.401542205	-0.798552833	XTH7	Probable xyloglucan endotransglucosylase/hydrolase 7 [Acc:Q8LER3]	7
AT1G72060	0.783863693	0.840416951	1.248095974	0.801355776	0.669856377	0.498611988	AT1G72060	Serine-type endopeptidase inhibitor [Acc:Q9C7G9]	7
AT1G76800	0.915192958	0.555233993	1.247770272	-0.19179483	0.372433626	0.221773093	AT1G76800	Vacuolar iron transporter homolog 2 [Acc:Q9SRD3]	7
AT3G06750	0.206988968	0.433817275	1.244232595	0.416410989	0.81236749	0.795945939	AT3G06750	F3E22.11 protein [Acc:Q9M7Y1]	7
AT1G54200	0.409485915	0.070816691	1.243450296	-0.173854286	0.871819711	-0.036819671	AT1G54200	Protein BIG GRAIN 1-like B [Acc:Q9SLL2]	7
AT1G69160	-0.230361185	0.494285873	1.243143759	0.162039799	0.268073636	0.04650193	AT1G69160	Protein BIG GRAIN 1-like E [Acc:Q93Z37]	7
AT1G51400	0.632935721	0.985130314	1.237188171	0.057679583	0.478503027	0.177395205	AT1G51400	At1g51400/F5D21_10 [Acc:Q9SYE2]	7
AT3G50410	0.488188146	0.93314547	1.233929678	0.355322151	0.438690317	-0.411750741	DOF3.4	OBP1 [Acc:A0A178VFT5]	7
AT1G79760	0.56717764	0.750220572	1.230042136	0.226584153	-0.622193402	-0.57487944	DTA4	Downstream target of AGL15-4 [Acc:Q9MA02]	7
AT5G58330	0.814133376	0.299437799	1.226199973	-0.046460728	0.126013437	0.087998242	AT5G58330	Malate dehydrogenase [NADP], chloroplastic [Acc:Q8H1E2]	7
AT4G18050	0.096768348	-0.209307581	1.223772412	-0.205959825	-0.379593662	0.206135771	PGP9	P-glycoprotein 9 [AT4G18050]	7
AT4G16980	0.84969658	0.357182949	1.221484356	-0.245583205	0.329937966	0.108695118	AT4G16980	Arabinogalactan family protein [Acc:Q9FT76]	7
AT5G63580	-0.211170748	-0.258888621	1.21948927	0	0	0	FLS2	Putative inactive flavonol synthase 2 [Acc:B2GVM7]	7
AT5G23380	0.44677823	0.384880722	1.219267916	0.063444862	-0.273347745	0.118980504	AT5G23380	Gb [Acc:Q9FGE1]	7
AT5G20380	0.222458751	0.001993957	1.214436942	-0.088934902	0.092034391	0.244918886	PHT4;5	phosphate transporter 4;5 [AT5G20380]	7
AT2G47400	0.877732349	0.64550345	1.214200756	-0.020773694	0.101495549	-0.075655707	CP12-1	Calvin cycle protein CP12-1, chloroplastic [Acc:O22914]	7
AT3G26550	0.374793895	-0.214250258	1.210038715	0.572004226	1.348080602	1.283158703	AT3G26550	CHP-rich zinc finger protein-like [Acc:Q9LRV1]	7
AT1G02860	0.399385562	0.091788774	1.20482935	0.754677434	0.773263087	0.940555627	BAH1	E3 ubiquitin-protein ligase BAH1 [Acc:Q9SRX9]	7
AT4G14060	0.181189312	0.550065711	1.201980749	0	0	0	AT4G14060	AT4g14060/dl3070w [Acc:O23267]	7
AT1G78290	0.535669035	-0.584594789	1.199558513	-0.647830331	-0.203514755	-0.240515298	SRK2C	SRK2C [Acc:A0A178W6S8]	7
AT2G40920	0.365121051	0.191641361	1.19102599	0.120650541	0.074331411	0.059455878	AT2G40920	F-box/LRR-repeat protein At2g40920 [Acc:Q2V414]	7
AT5G51010	0.222920775	0.101708642	1.190717067	-0.272824579	-0.158123454	-0.149287862	AT5G51010	Rubredoxin-like superfamily protein [Acc:Q9FI47]	7
AT2G34490	0.214270081	0.654199303	1.190710114	-0.815636047	0.19317725	-0.65318044	CYP710A2	Cytochrome P450 710A2 [Acc:O64698]	7
AT1G60550	0.432026946	0.167061878	1.18851296	0.178797142	0.047110704	0.445548305	MENB	1,4-dihydroxy-2-naphthoyl-CoA synthase, peroxisomal [Acc:Q8GYN9]	7
AT4G02290	-0.088896712	-0.201689334	1.185610021	-0.056920781	-0.218679956	-0.06924067	AtGH9B13	Endoglucanase 17 [Acc:O81416]	7
AT4G37150	0.720731531	0.541582849	1.185063526	-0.404680336	0.742162818	-0.132938602	MES9	MES9 [Acc:A0A178V387]	7
AT1G67710	0.129628995	-0.147239782	1.18297634	-0.354395337	-0.168301183	-0.002803582	ARR11	Two-component response regulator [Acc:Q0WRT0]	7
AT1G54500	0.69075845	0.321455949	1.182523705	-0.406168988	-0.339319623	-0.291518949	AT1G54500	At1g54500/F20D21_31 [Acc:Q9SLI4]	7
AT3G53420	0.875986321	0.934039014	1.182379612	0.273704833	0.910105249	0.445150005	PIP2-1	PIP2A [Acc:A0A178V8P4]	7
AT4G25080	0.538092438	0.418943757	1.181531699	0.08131592	0.113261036	0.137194148	CHLM	magnesium-protoporphyrin IX methyltransferase [AT4G25080]	7
AT2G31160	-0.038606196	0.307836025	1.180652209	-0.011243497	-0.31616582	-0.475650381	LSH3	Protein LIGHT-DEPENDENT SHORT HYPOCOTYLS 3 [Acc:O82268]	7
AT4G08150	-0.308750639	-0.801731976	1.178784988	-0.019574272	-0.041405971	0.410253587	KNAT1	Homeobox protein knotted-1-like 1 [Acc:P46639]	7
AT5G18100	0.901144443	0.8735991	1.177521831	0.492254028	0.269639839	0.431934946	CSD3	Superoxide dismutase [Cu-Zn] 3 [Acc:Q9FK60]	7
AT3G62740	-0.449990662	-0.9908947	1.176608995	0.969752059	0.325338541	1.652828664	BGLU7	Beta-glucosidase 7 [Acc:Q9LZJ1]	7
AT3G26220	0.473482626	0.741785784	1.175098851	0.327911351	0.794650752	0.377181275	CYP71B3	Cytochrome P450 71B3 [Acc:O65785]	7
AT5G52970	0.576821885	0.066938353	1.174218166	0.089081999	-0.05902547	0.047173193	AT5G52970	Thylakoid lumenal 15.0 kDa protein 2, chloroplastic [Acc:Q9LVV5]	7
AT1G21680	0.776158136	0.916123454	1.170407197	0.551708504	0.049412982	0.407081099	AT1G21680	DPP6 N-terminal domain-like protein [Acc:Q9XI10]	7
AT5G03720	0.357431013	0.490849543	1.169727177	0.660535443	0.119014436	0.282105542	HSFA3	HSFA3 [Acc:A0A178UHR4]	7
AT1G52870	0.613885045	-0.017461827	1.165817631	-0.252659234	0.131190378	0.346817516	AT1G52870	At1g52870/F14G24_14 [Acc:Q9C933]	7
AT5G35790	0.053745852	-0.416993325	1.163673993	-0.101411024	-0.043989044	0.052510743	G6PD1	Glucose-6-phosphate 1-dehydrogenase 1, chloroplastic [Acc:Q43727]	7
AT1G63240	0.022982297	0.180940961	1.162655712	0.208344174	0.097085501	0.028484168	AT1G63240	Uncharacterized protein At1g63240 [Acc:Q949Y1]	7
AT1G23740	0.905531263	0.56038809	1.162429912	-0.458991276	0.088820391	-0.223246054	AOR	NADPH-dependent alkenal/one oxidoreductase, chloroplastic [A:Q9ZUC1]	7
AT5G60490	0.227050044	0.340985128	1.161166233	-0.471915433	0.008517625	-0.136848823	FLA12	Fasciclin-like arabinogalactan protein 12 [Acc:Q8LEE9]	7
AT2G40460	-0.058490417	0.266389431	1.160918961	-0.829115318	-0.099052844	-0.730738922	NPF5.1	Protein NRT1/ PTR FAMILY 5.1 [Acc:Q8VZR7]	7
AT2G21050	0.389172341	0.560330892	1.156216797	-0.120067462	0.037255871	-0.027330609	LAX2	Auxin transporter-like protein 2 [Acc:Q9S836]	7
AT1G13700	0.179877856	0.242467182	1.153634279	0.372515134	0.345633514	0.232585355	PGL1	Probable 6-phosphogluconolactonase 1 [Acc:Q9LMX8]	7
AT4G25707	0.715837435	-0.591172961	1.153608935	0.426711054	0.426163577	0.486315489	AT4G25707	unknown protein; Ha. [AT4G25707]	7
AT2G36000	0.276648038	0.270256472	1.152724334	0.157778915	0.086603282	0.165414225	AT2G36000	At2g36000/F11F19.9 [Acc:Q9SJ50]	7
AT2G40530	0.085359213	0.68450339	1.152108298	1.629954029	1.164576708	1.557578486	CEP15	Precursor of CEP15 [Acc:O22882]	7
AT4G34030	0.381920754	-0.18922202	1.147260739	0.398299469	0.216214915	0.646386427	MCCB	Methylcrotonoyl-CoA carboxylase beta chain, mitochondrial [Acc:Q9LDD8]	7
AT4G03210	0.274379682	0.349072589	1.144857846	-0.417933126	-0.147037706	-0.654474746	XTH9	Xyloglucan endotransglucosylase/hydrolase (Fragment) [Acc:C0SVH2]	7
AT2G39310	0.796634056	0.412310347	1.143887525	0.143488833	0.959756102	0.33182219	JAL22	Jacalin-related lectin 22 [Acc:O80950]	7
AT5G48775	0.496373451	0.094487303	1.139512951	-0.156891518	0.044944095	0.038388	AT5G48775	other RNA [AT5G48775]	7
AT4G37550	0.014821803	-0.450954987	1.138554962	-0.079005801	-0.071799114	-0.038548445	AT4G37550	Acetamidase/Formamidase family protein [AT4G37550]	7
AT3G25710	0.267232279	0.70183051	1.137221222	0.192158279	-0.446089046	0.416824116	BHLH32	Transcription factor AIG1 [Acc:Q9LS08]	7
AT1G17460	0.220298149	0.436354017	1.135091333	0.596339666	0.036218705	0.450980131	TRFL3	TRF-like 3 [Acc:F4I7K3]	7
AT1G68585	0.151128518	0.517605528	1.131750557	0.104617628	0.446633364	-0.044860162	AT1G68585	At1g68585 [Acc:Q8LGG7]	7
AT5G03680	-0.003904824	-0.014053629	1.130275412	-0.187492954	0.406136591	-0.036054261	PTL	Trihelix transcription factor PTL [Acc:Q9LZS0]	7
AT1G64430	0.383340879	-0.592397809	1.127420777	0.148282765	-0.117889604	0.359512008	AT1G64430	Pentatricopeptide repeat (PPR) superfamily protein [Acc:Q8VYZ8]	7
AT5G51110	0.526022795	0.174716632	1.12429192	-0.242170125	-0.309801901	-0.242004967	ATP1	Probable pterin-4-alpha-carbinolamine dehydratase, chloroplastic [A:Q9LU63]	7
AT5G25140	1.187338334	0.319568562	1.124028732	-0.225477641	1.124091505	0.561646919	CYP71B13	Cytochrome P450 71B13 [Acc:P58050]	7
AT5G48620	-0.014627131	-0.791942793	1.122991551	0.117397998	-0.250370275	0.306913592	RPP8L4	Probable disease resistance RPP8-like protein 4 [Acc:Q9FJK8]	7
AT2G44290	0.815583778	0.521021371	1.120296871	-0.064679879	-0.265450522	0.400180073	YLS3	Protein YLS3 [Acc:O64864]	7
AT5G48800	-0.007023425	0.087270275	1.117763956	0.140485661	0.207666413	0.150268119	AT5G48800	BTB/POZ domain-containing protein At5g48800 [Acc:Q9FKB6]	7
AT3G21090	-0.217742468	0.011218299	1.117599349	0.315223484	0.277066428	-0.039249832	ABCG15	ABC transporter G family member 15 [Acc:Q8RWI9]	7

AT3G10200	-0.007549676	-0.158332732	1.117019393	-0.076845425	-0.299831004	-0.083035274	AT3G10200	S-adenosyl-L-methionine-dependent methyltransferases superfamily protein	7
AT4G18780	0.311019057	0.52210076	1.115279559	-0.311641123	-0.400857626	-0.116521926	CESA8	Cellulose synthase (Fragment) [Acc:A0A068FHR7]	7
AT3G03870	0.612235035	0.629822499	1.107264354	0.497729821	0.868727617	0.643599651	AT3G03870	F20H23.8 protein [Acc:Q9SRW4]	7
AT4G34160	-0.37160686	0.246900169	1.100148656	-0.054160656	0.192971451	-0.044118493	CYCD3-1	Cyclin-D3-1 [Acc:P42753]	7
AT5G67030	0.830304394	0.443737477	1.096207783	-0.584292186	-0.099118293	0.092041458	ZEP	Zeaxanthin epoxidase, chloroplastic [Acc:Q9FGC7]	7
AT1G01770	0.562450869	-0.39645238	1.096081722	0.349194182	0.17006028	0.605957858	AT1G01770	Propionyl-CoA carboxylase [Acc:Q8GZ85]	7
AT1G80060	0.411152027	-0.671875865	1.09105593	0.386773121	-0.157348691	-0.190971549	AT1G80060	At1g80060 [Acc:Q5XEN2]	7
AT3G18080	-0.058704998	0.052812526	1.090513982	-0.211182822	-0.159273413	0.108523111	BGLU44	Beta-glucosidase 44 [Acc:Q9LV33]	7
AT4G04630	-0.076865364	0.466005205	1.090252738	0.351802828	0.085103306	0.416968722	AT4G04630	Putative uncharacterized protein [Acc:Q8LFI5]	7
AT3G01860	0.014273206	-0.19799911	1.089825698	-0.226336571	-0.299463807	-0.398938117	AT3G01860	Uncharacterized protein At3g01860 [Acc:Q0WMJ7]	7
AT5G06290	0.707947183	0.796300913	1.089031877	-0.026596018	-0.038040846	0.074970052	2-Cys Prx B	2-Cys peroxiredoxin BAS1-like, chloroplastic [Acc:Q9C5R8]	7
AT5G14450	-0.025624713	0.186384203	1.087929958	0.090791477	0.111816976	-0.3267193	AT5G14450	GDSL esterase/lipase At5g14450 [Acc:Q9LY84]	7
AT3G52060	0.393143345	0.286838222	1.086754101	0.217035326	0.206013113	0.31994857	AT3G52060	GnTL [Acc:A0A384L2P4]	7
AT1G14730	-0.05310657	-0.04223657	1.083430832	0.859932695	0.91627095	0.391271663	CYB561C	Probable transmembrane ascorbate ferrireductase 3 [Acc:Q67ZF6]	7
AT5G38850	0.900317393	0.598111776	1.079737657	0.009252006	0.266827437	0.530462787	AT5G38850	Disease resistance protein (TIR-NBS-LRR class) [Acc:Q9FMB7]	7
AT1G13610	0.033184754	0.037453937	1.077627683	0	0	0	AT1G13610	Alpha/beta-Hydrolases superfamily protein [Acc:Q9FZ68]	7
AT4G19830	0.438465667	0.03943719	1.074536936	0.064584284	-0.272079719	-0.069793025	FKBP17-1	Peptidyl-prolyl cis-trans isomerase FKBP17-1, chloroplastic [Acc:O81864]	7
AT4G34480	0.540915874	0.559379291	1.071440524	0.419723791	0.60740468	0.852662253	AT4G34480	Glucan endo-1,3-beta-glucosidase 7 [Acc:Q9M069]	7
AT3G53950	0.315439599	0.574261073	1.07107715	0.448218775	0.40323179	0.242369756	AT3G53950	AT3g53950/F5K20_250 [Acc:Q9M332]	7
AT1G06040	0.512434749	0.689354897	1.070832536	0.017955839	0.179204803	0.071259645	BBX24	B-box zinc finger protein 24 [Acc:Q96288]	7
AT3G52840	-0.23031415	-0.651848756	1.066826115	-0.172500872	0.115276549	-0.033671191	BGAL2	Beta-galactosidase [Acc:A0A1I9LM56]	7
AT4G19950	0.292465968	0.144599618	1.063730036	1.106136318	1.430547874	0.733320773	AT4G19950	At4g19950 [Acc:Q6DBH7]	7
AT1G80640	-0.006805942	-0.045667521	1.06232374	-0.224310799	-0.107131826	-0.016245844	AT1G80640	Probable receptor-like protein kinase At1g80640 [Acc:Q0V7T5]	7
AT1G06570	0.794423575	0.574583206	1.059244861	0.164739302	0.136246975	0.434091311	HPD	4-hydroxyphenylpyruvate dioxygenase [Acc:P93836]	7
AT4G03230	0.359663623	-0.199161605	1.058646135	0.522525487	0.900461893	0.893191634	AT4G03230	S-locus lectin protein kinase family protein [AT4G03230]	7
AT3G10080	0.212749058	0.43597112	1.057966558	0.25676483	0.719813839	0.480975667	AT3G10080	RmlC-like cupins superfamily protein [Acc:A0A1I9LSI8]	7
AT1G02640	-0.090025395	-0.408705576	1.056406242	0.305158083	0.059281473	0.189443886	BXL2	Probable beta-D-xylosidase 2 [Acc:Q94KD8]	7
AT1G60390	-0.435011293	0.208739638	1.054871263	0.211929847	0.305193422	0.062551152	PGL2	Polygalacturonase 1 beta-like protein 2 [Acc:O80760]	7
AT3G47620	0.094996547	0.371924733	1.048502946	0.301508886	0.408290348	0.416227722	TCP14	Transcription factor TCP14 [Acc:Q93Z00]	7
AT1G56612	0.267983431	-1.017878056	1.046802913	0.532631265	0.180699155	0.437544586	AT1G56612	other RNA [AT1G56612]	7
AT3G51870	0.126611926	0.00286704	1.045209531	0.242121388	-0.069876784	0.163394973	EAAC	Probable envelope ADP,ATP carrier protein, chloroplastic [Acc:O65023]	7
AT3G20100	0.186556338	0.345596349	1.044373358	-0.072318104	0.771281098	0.190181419	CYP705A19	AT3g20100/MAL21_14 [Acc:Q9LJY8]	7
AT5G44190	0.869000999	0.039135156	1.044093763	-0.25413805	-0.169273998	0.124483696	GLK2	Transcription activator GLK2 [Acc:Q9FFH0]	7
AT3G56880	0.535075336	0.76706083	1.042785547	0.843248821	0.467500841	0.623008288	AT3G56880	VQ motif-containing protein [Acc:Q9LES0]	7
AT1G80160	0.713231203	0.008869989	1.030216446	0.083568386	0.501829186	0.408753533	AT1G80160	F18B13.24 protein [Acc:Q9SSC3]	7
AT2G28410	0.05009273	0.609158815	1.029851124	0.039713339	0.189154277	-0.336239985	AT2G28410	Expressed protein [Acc:Q9SKM9]	7
AT1G17020	0.805223116	0.712613646	1.029811481	-0.438865128	1.066635436	-0.058657345	SRG1	Protein SRG1 [Acc:Q39224]	7
AT2G04850	-0.314066905	-0.047728569	1.027685472	-0.181669744	-0.26447182	-0.627343364	AT2G04850	Cytochrome b561 and DOMON domain-containing protein [Acc:Q9SJ74]	7
AT5G01670	0.393297046	0.071711828	1.022615008	0.515985967	0.103125154	0.582979947	AT5G01670	NAD(P)-linked oxidoreductase superfamily protein [Acc:F4K9G7]	7
AT1G12280	0.26128911	-0.375348746	1.0219801	-0.035261275	-0.347106869	0.07631432	SUMM2	Disease resistance protein SUMM2 [Acc:P60838]	7
AT4G38020	0.391814232	0.258935248	1.020716332	0.225836208	0.127827782	0.105727906	AT4G38020	At4g38020 [Acc:Q8GYL4]	7
AT2G40640	0.025253168	-0.204855925	1.018001432	0.588140496	-0.027898197	0.452875989	PUB63	U-box domain-containing protein 63 [Acc:Q58FY4]	7
AT2G46220	0.560234586	0.240478323	1.014490756	0.238645648	0.197559412	0.379825156	AT2G46220	At2g46220/T3F17.13 [Acc:O82347]	7
AT1G06473	0.222923141	0.246043268	1.0141492	0.118578666	0.455388606	0.191273986	AT1G06473	None	7
AT4G28490	0.015485383	0.320348568	1.011987401	-0.006530674	0.382775084	0.109712436	RLK5	Receptor-like protein kinase 5 [Acc:P47735]	7
AT4G00780	0.808594008	0.871059647	1.011629797	-0.094774979	0.106528687	-0.017684176	AT4G00780	TRAF-like family protein [Acc:Q8VZ38]	7
AT4G14720	0.337933544	-0.157162615	1.009859823	0.448391082	-0.275566294	0.3047292	PPD2	TIFY domain/Divergent CCT motif family protein [AT4G14720]	7
AT1G73655	0.773165289	0.846884985	1.005857264	0.06828665	-0.107685502	-0.157360988	FKBP17-3	Peptidylprolyl isomerase [Acc:A0A178WAL2]	7
AT5G07030	-0.07773062	0.413172373	1.005100414	-0.338297402	-0.146964205	-0.327001352	AT5G07030	Eukaryotic aspartyl protease family protein [Acc:F4K5B9]	7
AT5G40910	0.011163002	-0.918526779	1.005063588	0.314637093	-0.148641025	0.341355892	AT5G40910	Disease resistance protein (TIR-NBS-LRR class) family [Acc:Q9FKR7]	7
AT2G33060	-0.014317091	-0.362159233	1.004949194	0.425493057	0.698084548	0.687128081	AtRLP27	Receptor like protein 27 [Acc:F4IUU1]	7
AT1G55510	0.341458823	-0.334327024	1.002116052	0.341889924	0.166453608	0.507187751	BCDH BETA1	2-oxoisovalerate dehydrogenase subunit beta 1, mitochondrial [Acc:Q9SAV3]	7
AT4G21480	0.50731934	0.706677647	1.001602222	0.468116799	-0.147452165	0.212726113	STP12	STP12 [Acc:A0A178V2V7]	7
AT1G02205	2.176504826	0.350418462	-0.473127302	-1.100833244	0.335755971	-1.49471132	CER1	Fatty acid hydroxylase superfamily [AT1G02205]	8
AT2G27390	7.179772418	-1.929317142	-1.929317142	0	0	0	AT2G27390	At2g27390 [Acc:Q9XIP3]	8
AT3G02480	3.013045827	1.094285451	0.039072931	0.716259621	1.84386937	-0.87693276	AT3G02480	AT3g02480/F16B3_11 [Acc:Q9M892]	8
AT5G65080	2.539584727	0.901327806	0.524842137	3.698543178	0.218872981	-0.503968808	MAF5	K-box region and MADS-box transcription factor family protein [AT5G65080]	8
AT2G23110	2.173890691	1.704273437	1.652631601	1.621094633	2.274266198	1.941720128	AT2G23110	At2g23110 [Acc:O64820]	8
AT2G21820	1.941383806	1.527626452	0.957123463	-0.38669785	0.447215067	-0.40321346	AT2G21820	At2g21820 [Acc:Q9SJ17]	8
AT5G67140	1.598733677	1.153966765	0.907651288	-0.091408227	0.516641648	0.07608104	AT5G67140	F-box protein At5g67140 [Acc:Q9FH99]	8
AT5G45810	1.567944839	0.198100086	-0.087114433	0	0	0	CIPK19	Non-specific serine/threonine protein kinase [Acc:A0A178UQC9]	8
AT3G44326	1.477818725	1.169824684	0.789793659	0	0	0	AT3G44326	F-box protein At3g44326 [Acc:Q2V3R1]	8
AT4G40010	1.386976726	-0.003235329	0.395483707	-0.229453589	0.484205115	-0.832088793	SRK2F	Serine/threonine-protein kinase SRK2F [Acc:Q9SMQ4]	8
AT5G43570	1.337960792	0.560315994	0.662692833	0	0	0	AT5G43570	Serine protease inhibitor, potato inhibitor I-type family protein [AT5G43570]	8
AT2G01530	1.30763681	0.419158863	0.409763162	0.298920643	0.45107305	0.269706406	MLP329	Uncharacterized protein At2g01530 (Fragment) [Acc:C0SV41]	8
AT4G40070	1.252833337	0.940368747	0.472158804	-0.230055815	0.497562497	0.594917033	ATL32	RING-H2 finger protein ATL32 [Acc:Q8W571]	8
AT5G54300	1.244075259	0.552751057	0.878944167	0.64355107	0.459078949	0.251377703	AT5G54300	Cotton fiber expressed protein 1-like protein [Acc:Q9FL70]	8
AT4G32590	1.236103005	0.958521037	0.84613794	0.33950485	0.564679762	0.407416392	AT4G32590	2Fe-2S ferredoxin-like superfamily protein [Acc:F4JUE0]	8
AT1G64610	1.109557313	0.359426782	0.869727351	0.374840673	0.253717267	0.266734452	AT1G64610	Transducin/WD40 repeat-like superfamily protein [Acc:Q8VZK1]	8
AT2G40230	1.105413293	0.982527365	0.637824298	0.246536732	0.80997645	-0.692929746	AT2G40230	HXXXD-type acyl-transferase family protein [Acc:Q9XEF2]	8
AT5G24930	1.077465988	0.79742581	0.743843653	0.031477435	0.052544284	0.104029223	COL4	Zinc finger protein CONSTANS-LIKE 4 [Acc:Q940T9]	8
AT4G09760	1.059694881	0.42967768	0.865787042	-0.11051889	0.313625773	0.025592935	AT4G09760	Probable choline kinase 3 [Acc:Q9SZ92]	8
AT5G10300	1.050548811	0.979961412	0.971467736	-0.060851062	0.616775028	0.235653657	HNL	Alpha-hydroxynitrile lyase [Acc:Q9LFT6]	8
AT1G07150	1.024278181	0.613587807	0.427876035	0.515583319	0.804322391	0.238774641	MAPKKK13	F10K1.14 protein [Acc:Q9LMK8]	8
AT1G34760	-0.174755844	-0.74400658	-1.105886549	0.423255512	0.7944041	1.017842499	GRF11	general regulatory factor 11 [AT1G34760]	9
AT5G52882	-0.699737457	-0.576084674	-1.003833319	-0.654365816	-0.676632626	-1.014655158	AT5G52882	P-loop containing nucleoside triphosphate hydrolases superfamily	9
AT5G61030	-0.725368684	-0.000410839	-1.003986658	-0.533846326	0.064993339	-0.315095488	RBG3	GR-RBP3 [Acc:A0A178UBT5]	9
AT4G01480	-0.352909279	-0.121765336	-1.005530839	-0.851906792	-0.103698594	-0.045608057	PPA5	PPa5 [Acc:A0A178V127]	9
AT1G62380	-0.373212994	-0.233534999	-1.011113336	-0.10317793	0.306050925	-0.251914842	ACO2	1-aminocyclopropane-1-carboxylate oxidase 2 [Acc:Q41931]	9
AT3G46320	-0.349407571	0.368548159	-1.011216417	-0.814843606	0.054993772	-0.940504122	AT3G46320	Histone H4 [Acc:P59259]	9
AT5G39320	-0.438403476	0.010571046	-1.013603766	-0.603549646	-0.515291481	-0.750915344	UGD4	UDP-glucose 6-dehydrogenase 4 [Acc:Q9FM01]	9
AT1G02920	-0.309434384	0.351582667	-1.014248025	-0.082383013	0.843260131	-0.415291445	GSTF7	Glutathione S-transferase F7 [Acc:Q9SRY5]	9
AT1G12110	-0.273042413	0.225472372	-1.014991165	-0.876735119	-0.312894728	-0.846154792	NPF6.3	NRT1.1 [Acc:A0A178W8F7]	9
AT5G63980	-0.590189242	-0.211657425	-1.016296236	-0.395490874	-0.243265952	-0.362509411	SAL1	SAL1 phosphatase [Acc:Q42546]	9
AT5G61440	-0.693342646	-0.369178263	-1.019957579	0.428084967	-0.095922559	-0.026158781	ACHT5	Thioredoxin-like 1-2, chloroplastic [Acc:Q9XFI1]	9
AT5G45280	0.144676932	0.394377727	-1.022003963	0.154847163	-0.258572551	-0.291808163	PAE11	Pectin acetylesterase 11 [Acc:Q9FH82]	9
AT1G10682	-0.525008118	-0.572413999	-1.022129154	-0.323388961	0.283936724	-0.434089094	AT1G10682	other RNA [AT1G10682]	9
AT4G04940	-0.779003617	-0.614738579	-1.022175995	0.391929585	0.097040545	0.454967689	AT4G04940	Putative WD-repeat membrane protein [Acc:Q93YS7]	9
AT2G30930	-0.468159353	-0.113152864	-1.023510947	0.040288542	0.136395517	-0.534544197	AT2G30930	Expressed protein [Acc:O80858]	9
AT1G06475	-0.487625338	0.031476118	-1.023527918	-0.708021375	1.123930819	-1.95598894	AT1G06475	Transmembrane protein [Acc:Q6DSU2]	9
AT3G09940	-0.128696353	-0.153399824	-1.023673719	-1.261401538	0.780196033	-1.151768946	MDAR3	Monodehydroascorbate reductase 3 [Acc:Q9SR59]	9
AT4G19720	-0.071225578	-0.379080869	-1.024658403	0	0	0	AT4G19720	Glycosyl hydrolase family protein with chitinase insertion domain	9
AT3G18130	-0.414833841	0.026559658	-1.026964816	-0.565372509	-0.280086588	-0.486642311	RACK1C	Receptor for activated C kinase 1C [Acc:Q9LV28]	9
AT5G59850	-0.225304451	0.417789795	-1.028209591	-0.426767941	-0.005400522	-0.776011608	RPS15AF	40S ribosomal protein S15a-1 [Acc:P42798]	9
AT5G01765	-0.466643852	-0.539178271	-1.031385598	-0.034528521	0.199393711	-0.911643281	AT5G01765	None	9
AT5G40590	-0.343408711	-0.096734986	-1.036029575	0	0	0	AT5G40590	At5g40590 [Acc:Q9FM39]	9
AT1G58170	-0.755016898	-0.848633449	-1.036957807	-1.19874374	-0.092390404	-2.404761769	DIR19	Dirigent protein 19 [Acc:Q9C523]	9
AT2G19690	0.165206278	0.557110508	-1.039151013	-0.906584722	0.295075643	-0.416753915	PLA2-BETA	Phospholipase A2-beta [Acc:F4ITF6]	9
AT1G22065	-0.839121095	0.077841883	-1.043617383	0	0	0	AT1G22065	Putative uncharacterized protein [Acc:Q1G3T6]	9
AT1G31280	-0.865859684	-0.931002754	-1.044579559	-0.161334049	-0.286739699	-0.566788362	AGO2	Protein argonaute 2 [Acc:Q9SHF3]	9
AT3G27880	-0.999340879	-0.38964244	-1.049335127	0.072327019	0.252855463	-0.147434672	AT3G27880	At3g27880 [Acc:Q9LK89]	9
AT1G44800	-1.114575518	-0.708437998	-1.04966246	-0.943156174	0.36123594	-0.41996965	AT1G44800	WAT1-related protein At1g44800 [Acc:Q9LPF1]	9
AT5G66390	-0.072590232	-0.219765328	-1.049713442	0	0	0	PER72	Peroxidase [Acc:Q0WT45]	9
AT5G52220	-0.126247247	0.244602432	-1.056667661	-1.834038106	-0.149233655	-1.635904841	AT5G52220	Chromosome transmission fidelity-like protein [Acc:F4KEU4]	9
AT2G07687	0.079201228	0.617206068	-1.056695769	-0.194389722	-0.101578914	-0.389855583	AT2G07687	Cytochrome c oxidase, subunit III [AT2G07687]	9
AT5G14750	-0.335464933	0.009322311	-1.064772031	0.315640481	-0.037045059	0.19325928	WER	WER1 [Acc:A0A384KIP7]	9
AT4G13430	-0.82283445	-0.516821954	-1.065470416	-0.432771111	-0.368966111	-0.387845729	IIL1	3-isopropylmalate dehydratase large subunit, chloroplastic [Acc:Q94AR8]	9
AT4G25630	-0.540412019	-0.402290867	-1.069257134	-0.175658875	-0.048929077	-0.229959123	MED36A	Mediator of RNA polymerase II transcription subunit 36a [Acc:Q94AH9]	9
AT2G04170	-0.272250775	-0.172648531	-1.069834368	-1.193268995	-0.910492216	-0.109650821	AT2G04170	TRAF-like family protein [Acc:Q6RF45]	9
AT2G22000	-0.355413447	-0.407288838	-1.070952134	0.413668385	0.579091305	0.000224194	PEP6	Elicitor peptide 6 [Acc:Q9SIZ9]	9
AT1G20696	-0.318414597	-0.17314604	-1.073718476	-0.569582978	-0.640224309	-0.717998653	HMGB3	high mobility group B3 [AT1G20696]	9
AT1G22410	-0.600959656	-0.429595498	-1.074631521	-0.565631807	-0.509011655	-0.799423649	AT1G22410	Phospho-2-dehydro-3-deoxyheptonate aldolase [Acc:Q9SK84]	9
AT3G48340	-0.010424329	0.115814974	-1.074634579	0	0	0	CEP2	KDEL-tailed cysteine endopeptidase CEP2 [Acc:Q9STL4]	9
AT5G09530	0.01829643	-0.318780633	-1.075278644	0.684550461	1.017776723	0.30199799	PELPK1	PRP10 [Acc:A0A178UP14]	9
AT2G28960	-0.611006017	-0.760310365	-1.077689185	0	0	0	AT2G28960	Probable LRR receptor-like serine/threonine-protein kinase [Acc:C0LGL4]	9
AT5G09800	0.110317368	0.134999072	-1.081616737	0.308516754	0.668655588	0.279587111	PUB28	U-box domain-containing protein 28 [Acc:Q9LXE3]	9
AT2G46410	0.128938348	-0.01319318	-1.082745545	-0.516157577	0.576705632	-0.323461164	CPC	Transcription factor CPC [Acc:O22059]	9
AT2G46140	0.241760972	0.530094167	-1.084738301	-0.218027593	-0.136555968	-0.72595905	AT2G46140	Desiccation-related protein At2g46140 [Acc:O82355]	9
AT1G61360	-0.487382162	-0.237810487	-1.085468579	-0.165078272	-0.080043944	-0.806955057	AT1G61360	Serine/threonine-protein kinase [Acc:A0A178W9H1]	9
AT5G44820	-0.295471292	-0.576405549	-1.087169228	-0.709700229	-0.084272943	1.132979552	AT5G44820	At5g44820 [Acc:Q147R7]	9
AT5G45670	-0.967574362	-0.349439439	-1.088215554	0.355456172	0.489041393	0.322802973	AT5G45670	GDSL esterase/lipase At5g45670 [Acc:Q9FK75]	9
AT1G08580	-0.557202093	0.055792593	-1.088454111	-0.050365338	0.142134177	-0.136351144	AT1G08580	At1g08580 [Acc:Q9FRS7]	9
AT1G71750	-0.719829209	-0.211822125	-1.08870146	-0.023031151	-0.144553449	-0.782363679	HGPT	Hypoxanthine phosphoribosyltransferase [Acc:F4IA25]	9
AT5G59970	-0.116813028	0.37312103	-1.091559836	-0.162737877	-0.299170031	-0.92837827	AT5G59970	Histone H4 [Acc:Q6NR90]	9
AT5G23190	0.496742802	-0.332132252	-1.092340295	0	0	0	CYP86B1	Cytochrome P450 86B1 [Acc:Q9FMY1]	9
AT4G35630	-0.585708107	-0.089477109	-1.093116685	-0.30991051	-0.151881121	-0.726250887	PSAT1	Phosphoserine aminotransferase 1, chloroplastic [Acc:Q96255]	9
AT1G66160	-0.21292281	-0.027776249	-1.093755518	0.349424735	0.751641312	0.791843956	PUB20	U-box domain-containing protein 20 [Acc:Q9C8D1]	9
AT4G34810	0.201512766	0.195487843	-1.093871904	-1.231695354	-0.460244232	-1.380750779	AT4G34810	SAUR-like auxin-responsive protein family [AT4G34810]	9
AT3G08520	-0.061271252	0.821878226	-1.093991785	0.171496753	0.147859277	-0.630603229	RPL41G	60S ribosomal protein L41 [Acc:P62120]	9
AT5G52250	-0.23943534	-0.003998864	-1.098588253	0.327293966	0.125583113	0.323811127	RUP1	WD repeat-containing protein RUP1 [Acc:Q9LTJ6]	9
AT3G62460	-0.89674581	-0.362422248	-1.098762316	-0.488797769	-0.28688167	-0.727103975	AT3G62460	AT3g62460/T12C14_160 [Acc:Q9LZP4]	9
AT4G19550	-0.590538101	-0.899261283	-1.098809628	-0.000858718	-0.334025964	-0.550949761	AT4G19550	Zinc ion binding / transcription regulator [Acc:F4JT86]	9
AT5G26340	-0.18638876	-0.279941803	-1.100931892	0.261503801	-0.170625139	-0.319176938	STP13	STP13 [Acc:A0A178UHA7]	9
AT1G26270	-0.657272121	-0.745497514	-1.100976603	-0.044290751	-0.31421656	-0.297021224	PI4KG5	Phosphatidylinositol 4-kinase gamma 5 [Acc:Q9C671]	9
AT5G35320	-0.912164553	-0.82894366	-1.102481117	-0.421484672	-0.646614189	-0.75114956	AT5G35320	DBH-like monooxygenase [Acc:O65233]	9
AT1G52245	-0.493858651	-0.641183417	-1.103712581	0.113182179	0.083518564	0.140222954	AT1G52245	Dynein light chain [Acc:Q9M810]	9
AT3G54640	-0.606744747	-0.46013795	-1.103839428	-0.469342866	-0.19217443	-0.77476358	TSA1	Tryptophan synthase alpha chain, chloroplastic [Acc:Q42529]	9
AT1G74385	-0.376624285	0.548171335	-1.105030364	-0.917700099	-0.089292843	-0.497861526	AT1G74385	None	9
AT4G26200	-0.483075716	-0.087906755	-1.106622134	-0.635419065	-0.492696466	0.013007261	ACS7	1-aminocyclopropane-1-carboxylate synthase 7 [Acc:Q9STR4]	9
AT4G30320	0.419793263	-0.033263615	-1.117229604	0	0	0	AT4G30320	CAP (Cysteine-rich secretory proteins, Antigen 5, and Pathogenesis-related 1	9
AT1G71695	-0.075331694	0.024900014	-1.12167585	0.072453002	0.231781497	-0.167475536	PER12	Peroxidase [Acc:A0A178WB34]	9
AT2G47550	-1.035152479	-0.891660328	-1.126821869	0	0	0	PME20	Probable pectinesterase/pectinesterase inhibitor 20 [Acc:O22256]	9
AT2G19590	-1.045344526	-0.88107583	-1.12688375	-0.272419989	0.882658789	-0.910346419	ACO1	1-aminocyclopropane-1-carboxylate oxidase 1 [Acc:Q9ZUN4]	9
AT2G29460	-0.39089162	0.001109866	-1.12842641	0.571809879	1.003425171	-0.107243786	GSTU4	Glutathione S-transferase U4 [Acc:Q9ZW27]	9
AT4G15270	-0.136372228	-0.250592557	-1.130419435	0	0	0	AT4G15270	Glucosyltransferase-like protein (Fragment) [Acc:W8PV66]	9
AT2G32960	-0.641741092	-0.255658598	-1.130692069	-0.693777934	-0.285793329	-0.599403445	DSP2	Tyrosine-protein phosphatase DSP2 [Acc:Q84MD6]	9
AT2G04400	-0.528604563	-0.724725589	-1.13147186	-0.07676106	-0.060145022	-0.507919242	IGPS	Indole-3-glycerol phosphate synthase, chloroplastic [Acc:P49572]	9
AT3G13610	-0.441871703	0.013884251	-1.134385277	0.427658803	1.358411205	0.775007692	F6'H1	Feruloyl CoA ortho-hydroxylase 1 [Acc:Q9LHN8]	9
AT4G11350	-0.205705598	-0.571024833	-1.136685936	0	0	0	AT4G11350	At4g11350 [Acc:B6IDH2]	9
AT2G29450	-0.411186491	-0.564672644	-1.13999179	-0.184214707	0.413679588	-0.342156068	GSTU5	GSTU5 [Acc:A0A178VPP0]	9
AT2G47140	0.183089557	0.129201107	-1.1462329	0	0	0	SDR3B	Short-chain dehydrogenase reductase 3b [Acc:Q94K41]	9
AT1G68825	-0.34622566	0.009526094	-1.150446692	2.112019104	1.833001281	0.14118371	DVL5	DVL5 [Acc:Q6X5T8]	9
AT1G19020	-0.587148615	0.238962847	-1.150577715	0.388261621	-0.060421626	-0.786810572	AT1G19020	CDP-diacylglycerol-glycerol-3-phosphate 3-phosphatidyltransferase [Q8VYY6]	9
AT1G66280	-0.513606679	-0.258949449	-1.152460543	1.131775604	2.098984048	1.876528799	BGLU22	Beta-glucosidase 22 [Acc:Q9C8Y9]	9
AT3G54625	-0.050197466	-0.241199962	-1.153646716	-0.510827619	-0.130283912	-0.491485046	AT3G54625	other RNA [AT3G54625]	9
AT5G60950	0.031556047	0.295487046	-1.15811598	-0.416571306	0.339299883	-0.421930009	COBL5	COBRA-like protein 5 [Acc:Q9FME5]	9
AT5G04310	-0.554915038	0.302381203	-1.158729271	-0.112579286	-0.325784385	-0.522748009	AT5G04310	Pectate lyase [Acc:F4JW80]	9
AT4G02270	0.259243029	0.090831143	-1.159970221	0	0	0	RHS13	At4g02270 [Acc:O81417]	9
AT3G20015	-0.753519592	-0.474009395	-1.1640721	0.253669239	0.166893102	0.164038623	ASPG2	Protein ASPARTIC PROTEASE IN GUARD CELL 2 [Acc:Q9LHE3]	9

AT1G55205	-0.67088512	-0.150284065	-1.164256498	-0.102694238	0.193848363	-0.148040549	AT1G55205	unknown protein	9
AT3G50900	-0.340171039	0.186507014	-1.168765218	0.305732515	0.511290973	0.198080889	AT3G50900	At3g50900 [Acc:Q9SVK9]	9
AT5G28640	-0.599287107	-0.68045086	-1.169771599	-0.373262933	-0.194543064	-0.353225456	GIF1	GRF1-interacting factor 1 [Acc:Q8L8A5]	9
AT1G17190	-0.322125418	0.076727295	-1.171452271	-0.596027531	-0.669630269	-0.430857733	GSTU26	Glutathione S-transferase U26 [Acc:Q9SHH8]	9
AT2G31790	-0.865855888	-0.491291869	-1.172511294	-0.160432264	0.158754665	-0.128428234	UGT74C1	Glycosyltransferase (Fragment) [Acc:W8Q6Z8]	9
AT3G48460	-0.037894516	0.068143347	-1.173926441	-0.250911555	0.744677872	-1.055425116	AT3G48460	GDSL esterase/lipase At3g48460 [Acc:Q9STM6]	9
AT1G68850	-0.001364945	-0.445817841	-1.177787951	0	0	0	PER11	Peroxidase 11 [Acc:Q96519]	9
AT2G15310	-1.137160283	-0.043769993	-1.177918902	-0.350000836	-0.419669873	-1.475624735	ATARFB1A	Probable ADP-ribosylation factor At2g15310 [Acc:Q9SHU5]	9
AT5G15520	-0.169620919	0.293386996	-1.181393131	-0.147119295	-0.179532597	-0.364725297	RPS19B	40S ribosomal protein S19-2 [Acc:Q9LF30]	9
AT1G26840	-0.521109413	0.164341574	-1.181743014	-0.976078172	-0.88176419	-0.251423479	ORC6	Origin of replication complex subunit 6 [Acc:Q9ZVH3]	9
AT1G66270	-0.454850258	-0.324711131	-1.18271154	0.473249822	1.592357905	0.727883369	BGLU21	BGLU21 [Acc:A0A178WA93]	9
AT5G48540	-0.547420454	0.031948871	-1.183746863	-0.409129254	0.11666923	-0.157841445	CRRSP55	Cysteine-rich repeat secretory protein 55 [Acc:Q9LV60]	9
AT5G11740	-0.684323963	-0.131995186	-1.188024405	-0.108817309	0.527500907	-0.184409883	AGP15	ATAGP15 [Acc:C0SVP3]	9
AT1G26255	-1.028770375	0.153534631	-1.188189328	0	0	0	AT1G26255	None	9
AT1G63410	-0.052261711	-0.232266356	-1.188564705	0	0	0	AT1G63410	Protein LURP-one-related 4 [Acc:Q9SH27]	9
AT1G05250	-0.147638667	-0.347062656	-1.189229139	0	0	0	PER2	Peroxidase 1 [Acc:P0DI10]	9
AT4G37490	-0.591957474	-0.481514741	-1.18981695	-0.575369034	-0.398591539	-0.11289853	CYCB1-1	Cyclin-B1-1 [Acc:P30183]	9
AT1G11540	-0.618007587	0.145829869	-1.19143658	0	0	0	AT1G11540	Sulfite exporter TauE/SafE family protein [AT1G11540]	9
AT1G09932	-0.395267142	-0.460502982	-1.191929211	-0.856123717	-0.669034477	-1.067449583	AT1G09932	Phosphoglycerate mutase family protein [Acc:Q8GWG7]	9
AT1G62980	0.035744958	0.105571576	-1.199589973	0	0	0	EXPA18	Expansin-A18 [Acc:Q9LQ07]	9
AT3G56020	-0.011258454	0.552336211	-1.203741461	-0.187750728	0.199159274	-0.97334976	RPL41G	60S ribosomal protein L41 [Acc:P62120]	9
AT1G14220	-0.384913761	0.316434609	-1.2043299	0	0	0	AT1G14220	F7A19.32 protein [Acc:Q9XI64]	9
AT2G43510	-0.047102994	-0.020621926	-1.20444019	-0.804306545	0.696524197	-1.018062045	ATTI1	TI1 [Acc:A0A178VRC4]	9
AT2G38110	-0.277747035	-0.91749118	-1.208125633	0.299359152	-0.110530074	-0.13866658	GPAT6	Glycerol-3-phosphate 2-O-acyltransferase 6 [Acc:O80437]	9
AT1G74830	-0.069194372	-0.058000055	-1.210803862	2.142599313	1.800014044	1.661352452	MYOB6	Probable myosin-binding protein 6 [Acc:F4HVS6]	9
AT2G46600	-0.422988484	-0.095215435	-1.211100473	-0.130632137	0.022991462	-0.330473018	KIC	Calcium-binding protein KIC [Acc:Q9ZPX9]	9
AT5G48880	-0.861453487	-0.83732794	-1.21178922	-0.672085732	-0.768573814	-0.990153798	KAT5	PKT2 [Acc:A0A178UEC0]	9
AT3G11120	-0.230914849	0.217297817	-1.216047816	-0.208640691	0.263738638	-0.922861653	RPL41G	60S ribosomal protein L41 [Acc:P62120]	9
AT3G03130	-0.804599202	-0.569645117	-1.216221384	-0.457228954	0.326509153	-0.486304032	AT3G03130	LisH domain-like protein [Acc:Q9M9N2]	9
AT1G77330	-0.032805024	-0.138987779	-1.221829362	-0.244757939	0.227238285	-0.180853177	AT1G77330	1-aminocyclopropane-1-carboxylate oxidase 5 [Acc:Q0WPW4]	9
AT3G23325	-0.410141591	0.351365131	-1.223069425	-0.325385796	-0.219680753	-0.78954552	AT3G23325	Uncharacterized protein At3g23325 [Acc:Q9LW64]	9
AT5G63270	-0.175182905	0.173644259	-1.224882181	0	0	0	AT5G63270	RPM1-interacting protein 4 (RIN4) family protein [Acc:Q9FMJ6]	9
AT4G30530	-0.494089644	-0.304953578	-1.225804923	-0.440078277	0.157533331	-0.66657409	GGP1	GGP1 [Acc:A0A178V143]	9
AT4G30140	-0.113795437	-0.927297544	-1.226200755	-0.549001443	-0.902006561	-0.359359247	CDEF1	CDEF1 [Acc:A0A178V2L8]	9
AT5G25250	-0.709740486	-0.179649941	-1.226272945	-0.632950199	-0.272595493	-0.467671618	FLOT1	Flotillin-like protein 1 [Acc:Q501E6]	9
AT5G24850	-0.465304482	-0.948820009	-1.226814483	-0.215651904	-0.085886366	-0.123195676	CRYD	Cryptochrome DASH, chloroplastic/mitochondrial [Acc:Q84KJ5]	9
AT5G43540	0.385203225	-0.45447512	-1.228133131	0	0	0	AT5G43540	C2H2 and C2HC zinc fingers superfamily protein [Acc:Q9FIY6]	9
AT1G11185	-0.554009043	-0.24568805	-1.228692859	0	0	0	AT1G11185	other RNA [AT1G11185]	9
AT2G22860	-0.1884775	-1.048142992	-1.231143536	0.266378719	0.525503237	0.035552535	PSK2	Phytosulfokines 2 [Acc:O81003]	9
AT1G26250	-1.167409154	-0.038312382	-1.23158298	0	0	0	AT1G26250	Proline-rich extensin-like family protein [Acc:Q9C669]	9
AT5G60520	0.10947051	0.614623333	-1.232771293	0	0	0	AT5G60520	At5g60520 [Acc:Q9FKJ5]	9
AT5G40510	-0.269671537	-0.031319696	-1.233106165	0	0	0	AT5G40510	Sucrase/ferredoxin-like family protein [AT5G40510]	9
AT2G27840	-0.738716776	-0.401187071	-1.233954203	0.10914452	0.154237636	0.00581418	HDT4	Histone deacetylase HDT4 [Acc:Q9M4T3]	9
AT1G05260	-0.347329763	-0.31090537	-1.237154176	1.266480433	2.456957448	2.732152358	PER3	Peroxidase [Acc:Q0WSR2]	9
AT3G55120	-0.77461423	-0.740432888	-1.239612663	-0.006427208	0.517887689	0.27979267	CHI1	Chalcone--flavonone isomerase 1 [Acc:P41088]	9
AT4G24140	-0.319119005	-0.807269004	-1.239617428	-0.276186841	-0.42317978	-0.191842555	BDG3	Probable lysophospholipase BODYGUARD 3 [Acc:O22977]	9
AT5G23830	-0.615177365	-0.10068501	-1.241291277	-0.519472838	1.06630308	1.265058283	AT5G23830	MD-2-related lipid recognition domain-containing protein [Acc:Q9FF97]	9
AT5G49910	-0.793729858	-0.871564707	-1.244470803	-0.034417491	0.100730805	-0.06174349	HSP70-7	Heat shock 70 kDa protein 7, chloroplastic [Acc:Q9LTX9]	9
AT5G64810	-0.328181376	0.313978264	-1.245802725	0	0	0	WRKY51	Probable WRKY transcription factor 51 [Acc:Q93WU9]	9
AT5G42510	-0.260025647	0.139823463	-1.248524665	0	0	0	DIR1	Dirigent protein 1 [Acc:Q9FIG6]	9
AT2G47540	-0.487869658	0.09049627	-1.24941502	0	0	0	AT2G47540	Pollen Ole e 1 allergen and extensin family protein [Acc:O22257]	9
AT2G16760	-0.022977171	0.068445731	-1.250769709	0	0	0	AT2G16760	Calcium-dependent phosphotriesterase superfamily [Acc:Q9SLE2]	9
AT4G29690	-0.465518788	0.028149014	-1.254193532	1.376044803	1.510482316	1.45647027	AT4G29690	Alkaline-phosphatase-like family protein [Acc:Q9SU82]	9
AT5G06990	0.09369198	0.093448948	-1.255148303	1.032309733	0.311199294	0.512294257	AT5G06990	Expressed protein [Acc:Q9FL47]	9
AT5G38900	-0.113613983	0.110577725	-1.263353956	-0.974393005	0.445658926	-0.207916393	AT5G38900	Thioredoxin superfamily protein [AT5G38900]	9
AT2G30130	-0.399510317	0.359922251	-1.264638726	0	0	0	LBD12	PCK1 [Acc:A0A178VRK1]	9
AT4G26010	-0.115705452	-0.271571571	-1.265105204	0	0	0	AT4G26010	Peroxidase superfamily protein [AT4G26010]	9
AT2G45860	-0.139826891	-0.450013139	-1.265344772	0.159034698	0.103046448	-0.355609929	AT2G45860	At2g45860 [Acc:O80833]	9
AT2G37360	-0.330500463	-0.825432615	-1.268737162	-5.991079622	-0.085776833	5.220589661	ABCG2	ABCG2 [Acc:A0A178VW33]	9
AT1G02360	-0.69994699	-0.328034139	-1.271276091	-0.557311036	-0.080387848	-0.501283566	AT1G02360	Chitinase family protein [AT1G02360]	9
AT4G00680	-0.166246828	-0.167753909	-1.27262378	0	0	0	ADF8	actin depolymerizing factor 8 [AT4G00680]	9
AT4G24275	-0.42435257	-0.054815775	-1.275140076	0.381388179	0.710130849	0.138310427	AT4G24275	At4g24275 [Acc:Q84R17]	9
AT5G58860	-0.236436742	-0.678497475	-1.278696853	0	0	0	CYP86A1	Cytochrome P450 86A1 [Acc:P48422]	9
AT3G60330	-0.364909935	-0.826923064	-1.281548393	0.756249402	-0.463284036	1.214484864	AHA7	Plasma membrane ATPase [Acc:A0A1I9LS30]	9
AT2G23540	-0.249714383	-0.701945891	-1.289491249	0	0	0	AT2G23540	GDSL esterase/lipase At2g23540 [Acc:O80470]	9
AT1G33280	0.230620367	0.507569851	-1.294978902	0	0	0	BRN1	NAC015 [Acc:A0A178W4M7]	9
AT3G14940	-0.423202563	-0.4153789	-1.298165315	-0.618572229	-1.00273682	0.048362165	PPC3	Phosphoenolpyruvate carboxylase 3 [Acc:Q84VW9]	9
AT4G13310	-0.316741715	-0.679158028	-1.298473026	0	0	0	CYP71A20	Cytochrome P450 71A20 [Acc:Q9T0K2]	9
AT1G05730	-0.151054498	0.291762345	-1.303067016	0.381435212	0.319985749	0.860937709	AT1G05730	FAM136A-like protein (DUF842) [Acc:Q5BQ23]	9
AT5G59090	-0.378556304	-0.482281541	-1.305748996	-0.836206942	0.38360503	0.1456988	SBT4.12	Subtilisin-like protease SBT4.12 [Acc:Q8L7D2]	9
AT5G50300	-0.52583042	-0.335797979	-1.308902123	0	0	0	AZG2	Xanthine/uracil permease family protein [AT5G50300]	9
AT5G24140	0.139757334	-0.642058345	-1.313223367	0	0	0	SQP2	squalene monooxygenase 2 [AT5G24140]	9
AT5G43870	-0.76630918	-1.150558482	-1.314544917	-0.26951245	0.347094266	-0.18383663	AT5G43870	At5g43870 [Acc:Q9LKC2]	9
AT2G04025	-0.354160419	-0.690582853	-1.315946889	0	0	0	RGF3	Root meristem growth factor 3 [Acc:Q6ID76]	9
AT1G74460	-0.178427937	-0.26287214	-1.316701977	0	0	0	AT1G74460	GDSL esterase/lipase At1g74460 [Acc:Q9CA68]	9
AT4G15280	-0.234832412	-0.214684066	-1.319112368	0	0	0	UGT71B5	UDP-glucosyl transferase 71B5 [AT4G15280]	9
AT1G14160	0.077448854	-0.643929317	-1.32043168	0	0	0	AT1G14160	CASP-like protein 1A1 [Acc:Q9XI72]	9
AT5G09480	-0.464222238	-0.800085267	-1.322322124	0	0	0	AT5G09480	At5g09480 [Acc:Q9FY67]	9
AT3G01505	-0.507995228	-0.529860161	-1.323393267	-1.026487676	-0.237340577	-0.957478223	AT3G01505	None	9
AT4G19980	-0.827857155	-0.097633205	-1.323659341	0	0	0	AT4G19980	Putative uncharacterized protein [Acc:Q3E9Y5]	9
AT5G58750	-0.206704533	-0.68357077	-1.324182799	0	0	0	AT5G58750	At5g58750 [Acc:Q9LUY6]	9
AT3G51600	-0.307893454	-0.045322785	-1.327361747	-0.055643601	0.138419988	-0.50081873	LTP5	Non-specific lipid-transfer protein 5 [Acc:Q9XFS7]	9
AT2G02950	-1.164171114	-0.763087569	-1.33178633	-0.038105115	0.279403511	-0.154453928	PKS1	Protein PHYTOCHROME KINASE SUBSTRATE 1 [Acc:Q9SWI1]	9
AT4G24670	-0.355472421	-0.195333657	-1.332132472	0.19451109	0.474373101	0.353864048	TAR2	Tryptophan aminotransferase-related protein 2 [Acc:Q94A02]	9
AT1G23140	-0.668681846	-0.532695968	-1.332357416	-0.472885159	0.359052297	-1.040804718	CAR8	Protein C2-DOMAIN ABA-RELATED 8 [Acc:O49303]	9
AT1G53680	-0.266200575	-0.16796476	-1.332589787	0.943480926	1.108515222	1.391259256	GSTU28	Glutathione S-transferase U28 [Acc:Q9C8M3]	9
AT5G67400	-0.3510054	-0.374817187	-1.335296704	0	0	0	PER73	Peroxidase [Acc:A3KPG1]	9
AT4G24930	-0.127287509	-0.474876871	-1.337498549	-0.326100155	-0.267955663	-0.6037842	AT4G24930	Thylakoid lumenal 17.9 kDa protein, chloroplastic [Acc:Q9SW33]	9
AT1G66200	-0.213474237	-0.175217083	-1.34316513	-0.310462057	-0.013618531	-0.106237036	ATGSR2	Glutamine synthetase [Acc:F4ID91]	9
AT1G23410	-0.271270806	0.433293984	-1.346887079	0	0	0	RPS27AA	Ubiquitin-40S ribosomal protein S27a-1 [Acc:P59271]	9
AT1G13420	-1.373465682	-1.005054665	-1.347789941	0	0	0	SOT8	Cytosolic sulfotransferase 8 [Acc:Q9FX56]	9
AT4G02520	-0.750670302	-0.497678716	-1.348078604	-0.125906958	0.143728647	-0.070440545	GSTF2	Glutathione S-transferase F2 [Acc:P46422]	9
AT1G20160	-0.21339526	-0.021165695	-1.352141431	0.017783864	0.502086856	0.263340657	CRSP	CO(2)-response secreted protease [Acc:Q9LNU1]	9
AT2G32220	-0.089048104	0.455297734	-1.353531097	0.551786724	0.470793084	0.146982314	RPL27A	60S ribosomal protein L27-1 [Acc:Q9SKX8]	9
AT3G48450	-0.752378468	-0.87964928	-1.356452357	0	0	0	AT3G48450	At3g48450 [Acc:Q8LCF4]	9
AT4G32950	-1.661292538	-0.627077829	-1.360369762	-1.923248653	-1.141594512	-2.967380204	AT4G32950	Probable protein phosphatase 2C 61 [Acc:O82637]	9
AT5G06740	-0.92279313	-0.337777491	-1.364131423	0	0	0	AT5G06740	Concanavalin A-like lectin protein kinase family protein [AT5G06740]	9
AT4G19030	-0.227652663	0.154186281	-1.366626992	0	0	0	NIP1-1	NLM1 [Acc:A0A384L612]	9
AT4G25220	0.067920619	-0.392316146	-1.384451909	0	0	0	RHS15	Putative glycerol-3-phosphate transporter 2 [Acc:Q9SB41]	9
AT5G43380	-1.181740936	-1.18975729	-1.392322974	-0.670798987	-0.393418263	-0.472834355	TOPP7	Serine/threonine-protein phosphatase PP1 isozyme 7 [Acc:O82733]	9
AT1G02400	1.305033183	0.569533545	0.481324877	2.003077558	1.138131812	2.05334478	GA2OX6	Gibberellin 2-beta-dioxygenase 6 [Acc:Q9FZ21]	10
AT3G09032	-0.356855865	-0.366731335	0.152804001	2.164280618	1.551448585	1.846008726	AT3G09032	At3g09032 [Acc:Q6ID69]	10
AT3G24520	0.144679315	-0.408747307	-0.129238284	1.61959099	1.207548755	1.557187867	HSFC1	Heat stress transcription factor C-1 [Acc:Q9LV52]	10
AT4G14860	0.215599651	0.142973597	-0.444433845	1.559197754	1.182343919	1.921826828	OFP11	Transcription repressor OFP11 [Acc:O23341]	10
AT5G13870	0.38059154	0.945402866	0.133169754	1.497953222	0.456574087	1.306572584	XTH5	Xyloglucan endotransglucosylase/hydrolase [Acc:Q67Z90]	10
AT5G08240	0.296034704	0.415910039	0.165103427	1.479235051	0.919249008	1.188972002	AT5G08240	At5g08240 [Acc:Q9LEY3]	10
AT4G01670	0.746938929	0.72378784	0.006813817	1.470835142	0.615888705	1.524635948	AT4G01670	AT4G01670 protein [Acc:Q8GXD3]	10
AT4G29780	0.518852155	0.167111165	0.343600835	1.440301801	1.02097363	1.704474849	AT4G29780	Nuclease [Acc:Q84J48]	10
AT4G27260	0.763524154	-0.153157737	0.598373055	1.269086698	0.856219022	1.233170399	GH3.5	Indole-3-acetic acid-amido synthetase GH3.5 [Acc:O81829]	10
AT3G11420	-0.011152242	-0.101802798	-0.180370015	1.194142524	0.835796016	1.023538336	AT3G11420	At3g11420 [Acc:Q9CAY4]	10
AT5G06865	0.936882812	0.40712241	0.325100353	1.107747862	0.844546858	1.032512263	AT5G06865	other RNA [AT5G06865]	10
AT2G15730	0.26403438	-0.342993552	0.293267851	1.097867978	0.963969066	1.059275364	AT2G15730	At2g15730 [Acc:Q6NM29]	10
AT1G70210	-0.673749171	-0.751623196	-0.580575259	1.043876322	0.747755371	1.039231706	CYCD1-1	Cyclin-D1-1 [Acc:P42751]	10
AT4G33920	-0.418380351	-0.220856352	-0.430171676	1.007349222	0.723559329	1.163436735	AT4G33920	Probable protein phosphatase 2C 63 [Acc:O81760]	10
AT2G21180	1.152515029	1.370281658	1.129413133	0.752629728	1.085049281	0.717648378	AT2G21180	At2g21180/F26H11.6 [Acc:Q9SKP5]	11
AT5G02810	2.368592321	1.540000493	3.121995692	1.101077496	-0.186430448	1.391710507	APRR7	Two-component response regulator-like APRR7 [Acc:Q93WK5]	11
AT5G59310	3.633171016	3.191085238	3.484838586	-4.231734481	1.090229997	-3.761503381	LTP4	Non-specific lipid-transfer protein 4 [Acc:Q9LLR6]	11
AT2G36690	2.267007115	1.329327118	1.794874266	-0.959531228	-0.746971796	-0.300202647	AT2G36690	2-oxoglutarate (2OG) and Fe(II)-dependent oxygenase	11
AT1G16030	2.011317392	2.982080519	2.59890412	-0.403813329	0.176428896	-0.80078788	HSP70-5	Hsp70b [Acc:A0A178W9N7]	11
AT3G21520	1.812322512	2.201173371	1.847755572	0	0	0	DMP1	Protein DMP1 [Acc:Q9LVF4]	11
AT3G14620	1.812056563	1.729229105	1.933181228	0.67617652	0.874979246	0.526785968	CYP72A8	AT3g14620/MIE1_12 [Acc:Q9LUD2]	11
AT2G45220	1.753503167	1.359277051	1.491996545	-1.523321351	-0.101629433	-1.400762237	PME17	Probable pectinesterase/pectinesterase inhibitor 17 [Acc:O22149]	11
AT5G64170	1.636696191	1.518105816	1.760262885	0.329203675	0.549731986	0.553611526	LNK1	Protein LNK1 [Acc:A8MQN2]	11
AT2G21130	1.532124573	1.979560375	1.031381917	0.95492867	0.597079621	0.396087198	CYP19-2	Peptidyl-prolyl cis-trans isomerase CYP19-2 [Acc:Q9SKQ0]	11
AT5G57345	1.52900095	1.39595357	1.453336336	0.065325846	0.362230473	0.408988371	AT5G57345	At5g57345 [Acc:Q8LAD4]	11
AT2G43050	1.38490605	1.955137693	2.031130037	-0.450123585	-0.470966577	-0.4669341	PME16	Probable pectinesterase/pectinesterase inhibitor 16 [Acc:Q9SKX2]	11
AT1G53560	1.336472246	1.303433735	1.128948801	0.429587892	0.70502937	0.412625313	AT1G53560	At1g53560 [Acc:Q9LPH1]	11
AT1G22770	1.155735074	1.177153397	1.316926387	0.832964466	-0.423550233	0.496688262	GI	Protein GIGANTEA [Acc:Q9SQI2]	11
AT3G47500	1.003110041	1.086420155	1.431842934	-0.261066686	-0.298439713	-0.026951771	CDF3	Cyclic dof factor 3 [Acc:Q8LFV3]	11
AT5G56600	1.005674575	1.705248872	1.320284874	0.746691364	1.150404455	0.662578079	PRO5	Profilin-5 [Acc:Q9FE63]	11
AT2G46790	1.002818775	1.4928662	1.341229843	0.931702295	1.226764215	1.081308848	APRR9	Two-component response regulator-like APRR9 [Acc:Q8L500]	11
AT4G33720	-0.481336153	4.098788168	2.813569209	0	0	0	AT4G33720	AT4g33720/T16L1_210 [Acc:O81888]	11
AT1G70830	2.926243913	3.145674755	4.586486958	-0.124306939	0.152108642	-0.290732129	MLP28	MLP-like protein 28 [Acc:Q9SSK9]	11
AT5G07330	1.406978317	2.305216685	1.964978138	0	0	0	AT5G07330	At5g07330 [Acc:Q9LY27]	11
AT5G67430	1.660325838	1.893830057	1.891702571	0	0	0	AT5G67430	Acyl-CoA N-acyltransferases (NAT) superfamily protein [Acc:Q9FN10]	11
AT1G03070	1.140244053	1.845265302	1.438955219	0.787045218	1.221701126	1.172629867	LFG4	Protein LIFEGUARD 4 [Acc:Q9SA63]	11
AT1G05347	0.820064749	1.668424751	1.326685556	0.293502541	1.542049744	1.17899241	AT1G05347	None	11
AT1G28660	1.062224228	1.633298397	1.511136487	-0.312665595	0.219106962	-0.346067933	AT1G28660	GDSL esterase/lipase At1g28660 [Acc:Q9FPE4]	11
AT2G21320	0.59629458	1.398744245	1.277043088	0.078508223	0.36801236	-0.63655567	BBX18	B-box zinc finger protein 18 [Acc:Q9SJU5]	11
AT5G39090	1.155748122	1.396938677	1.221700578	0.027383765	0.163404869	-0.342858707	AT5G39090	Acyltransferase-like protein [Acc:Q9FID1]	11
AT3G12320	1.01545948	1.381826817	1.271423174	0.137371112	0.436795809	0.121010333	LNK3	LNK3 [Acc:A0A178VJK8]	11
AT5G06980	1.135535458	1.376925543	1.940125171	0.160568317	0.263339738	0.317537735	AT5G06980	unknown protein	11
AT1G47655	0.611115482	1.123764185	1.690156417	0.645212442	0.262705356	0.558784576	DOF1.6	Dof zinc finger protein DOF1.6 [Acc:Q9SX97]	11
AT5G01880	1.013959714	1.102172764	1.294418698	0.078848851	1.015122632	0.689268586	ATL74	RING-H2 finger protein ATL74 [Acc:Q9LZV8]	11
AT3G06470	0.437194879	1.04359374	1.052182035	0.116114315	0.289008961	0.159696773	AT3G06470	F24P17.3 protein [Acc:Q9SQV0]	11
AT2G26690	0.879442882	1.013729651	1.472436139	0.630306165	0.948624055	0.836044898	NPF6.2	Protein NRT1/ PTR FAMILY 6.2 [Acc:Q9SZY4]	11
AT5G10435	10.24649167	10.59381593		0	0	0	AT5G10435	Transmembrane protein [Acc:A0A1P8BC53]	12
AT1G15405	-0.92326292	4.14679001	-1.364609202	-2.028781669	-0.441676853	-2.481198936	AT1G15405	other RNA [AT1G15405]	12
AT2G03230	0.767374749	3.986566014	-0.425822306	0	0	0	AT2G03230	GCK domain-containing protein [Acc:O81051]	12
AT5G04305	-0.72172439	3.880508058	-5.178513639	0	0	0	AT5G04305	None	12
AT2G09465	-0.105540682	3.413527915	-2.323762459	0	0	0	AT2G09465	None	12
AT5G01515	-0.066876297	3.339130448	-3.516785879	0	0	0	AT5G01515	None	12
AT3G01715	0.774588398	3.103484077	-1.892220753	0	0	0	AT3G01715	None	12
AT5G09125	0.122846116	2.625396882	-1.369849915	0.039389323	-0.580614136	-1.577564359	AT5G09125	None	12
AT5G09585	-0.198323435	2.571355083	-1.355097506	0.207906041	0.503312066	-0.271762692	U2.5	U2.5; snRNA [AT5G09585]	12
AT3G12510	0.838064542	2.487857963	0.744824611	1.797926273	2.325600924	-6.650387515	AT3G12510	At3g12510 [Acc:Q9LHF8]	12
AT2G08760	-0.433353304	2.470425842	-1.883001231	-1.055919462	1.038211058	-0.491412847	AT2G08760	None	12
AT1G56600	1.915624393	2.338280677	0.638761116	0.005362712	1.058556441	-1.518009711	GOLS2	Galactinol synthase 2 [Acc:Q9FXB2]	12
AT2G06835	-1.582176858	2.299842276	-1.177738707	0	0	0	AT2G06835	None	12

AT1G09950	0.344134471	2.29789886	0.446412899	1.320933833	-0.056702674	1.359985577	RAS1	At1g09950 [Acc:O04515]	12
AT5G53902	0.727159133	2.225174089	0.573562517	0.047015086	0.093879593	-0.961813661	U3B	U3B; snoRNA [AT5G53902]	12
AT2G41342	0.459097011	2.225118082	1.193746736	0	0	0	AT2G41342	Putative uncharacterized protein [Acc:Q1G3R7]	12
AT1G07803	1.44860435	2.108004516	0.4800246	0.118098675	0.980385093	-0.60605285	AT1G07803	None	12
AT1G07430	1.560594174	2.087193809	1.251464372	0.131804952	0.551069929	-0.807331127	AIP1	Protein phosphatase 2C 3 [Acc:Q9LNW3]	12
AT5G09065	0.58025366	2.043265183	-0.216067136	0.532478409	0.927912373	0.124963158	AT5G09065	None	12
AT5G59220	1.744524406	1.930994827	1.166112403	-0.050794407	0.857196207	-0.21193047	SAG113	Probable protein phosphatase 2C 78 [Acc:Q9FIF5]	12
AT3G48510	1.47120478	1.924840561	0.137750327	0	0	0	AT3G48510	At3g48510 [Acc:Q9SMP6]	12
AT3G57765	0.520666225	1.921866653	-0.47367805	0.261118607	-0.209841197	-1.181322985	U2.3	U2.3; snRNA [AT3G57765]	12
AT4G39404	1.331220657	1.90036702	0.252510491	0	0	0	AT4G39404	other RNA [AT4G39404]	12
AT4G06795	0.968175554	1.887055264	-0.316685032	0	0	0	AT4G06795	None	12
AT1G09363	0.704950201	1.849853919	0.340180828	0.164562764	-0.439083717	-0.663860169	AT1G09363	None	12
AT3G03270	0.300856642	1.835518248	0.589587025	-0.079428448	0.745852512	0.249817219	AT3G03270	AT3G03270 protein [Acc:Q8LFK2]	12
AT5G03285	1.001970857	1.830838508	0.768855008	0.897864281	1.505443473	1.615971167	AT5G03285	other RNA [AT5G03285]	12
AT3G56825	-0.039590354	1.807221483	-0.72932732	0.373092811	0.410855381	-1.550789914	U2.4	U2.4; snRNA [AT3G56825]	12
AT4G29610	0.006978753	1.791483893	-0.438293253	2.143754547	-1.287057699	1.025931455	CDA6	Cytidine deaminase 6 [Acc:Q9SU86]	12
AT5G23230	1.287050642	1.759602629	1.347365501	1.255369167	0.954740259	1.106419614	NIC2	Nicotinamidase 2 [Acc:Q9FMX7]	12
AT5G54075	-0.18988929	1.720664577	-0.696651961	0.179128313	0.357779422	-1.282176319	U3D	U3D; snoRNA [AT5G54075]	12
AT2G18193	0.979039819	1.696662075	0.548311354	1.342791347	1.420585894	0.412399997	AT2G18193	AAA-ATPase At2g18193 [Acc:Q8GW96]	12
AT4G00165	0.78347427	1.657854014	0.762443475	-0.229413644	-0.235341959	-0.399023067	AT4G00165	Putative lipid-binding protein At4g00165 [Acc:Q8RW93]	12
AT1G47600	0.627293575	1.649757062	-1.338492781	0	0	0	TGG4	Myrosinase 4 [Acc:Q8GRX1]	12
AT1G34047	0.362756951	1.64268015	1.363739537	0	0	0	AT1G34047	Defensin-like (DEFL) family protein [Acc:F4HT37]	12
AT5G47450	1.42532572	1.608182039	1.036747959	-0.074523413	2.444441467	2.980212784	TIP2-3	Aquaporin TIP2-3 [Acc:Q9FGL2]	12
AT3G41762	0.349341862	1.574142009	-0.558849774	0.833999972	0.219775873	-0.28853574	AT3G41762	unknown protein. [AT3G41762]	12
AT5G65990	1.136657971	1.543502678	0.767088576	0.204716357	0.511172786	0.25526219	AVT3A	Amino acid transporter AVT3A [Acc:Q9FKY3]	12
AT4G36010	0.68121445	1.521337725	0.725515666	0.974901363	0.894004234	1.021455592	AT4G36010	Pathogenesis-related thaumatin superfamily [Acc:O65638]	12
AT2G01008	0.110500528	1.504435416	-0.368114744	1.027637541	-0.332155165	-0.747366229	AT2G01008	unknown	12
AT5G58070	0.818760543	1.497766353	0.676897138	0.43784536	0.832269194	0.733821069	TIL	TIL [Acc:A0A178US04]	12
AT4G12495	-0.187170336	1.491834229	-0.645400335	-1.25510758	-1.26173553	-1.503850506	AT4G12495	Transmembrane protein [Acc:A0A1P8B684]	12
AT3G08590	0.475011977	1.485825046	0.254637811	0.195144339	0.396647542	0.254497171	AT3G08590	IPGAM2 [Acc:A0A384KAQ5]	12
AT1G11700	0.706864095	1.462969335	0.626363264	0.030086395	0.713311559	0.059512136	AT1G11700	At1g11700 [Acc:Q9SAA7]	12
AT5G27660	0.312869027	1.43811341	-0.694319695	0.300709048	0.54434851	-0.403268281	AT5G27660	DEG14 [Acc:A0A178UPC0]	12
AT5G56540	0.544564477	1.435144502	0.039317646	-0.328737015	0.146112763	-0.507143084	AGP14	Arabinogalactan peptide 14 [Acc:Q9LVC0]	12
AT5G59320	0.409964894	1.395177344	0.37570736	-1.506680054	0.699148905	-2.558967132	LTP3	Non-specific lipid-transfer protein 3 [Acc:Q9LLR7]	12
AT2G28710	0.92629612	1.390006757	0.81846793	0.596343205	0.155676469	-0.222961319	AT2G28710	At2g28710 [Acc:Q9SI97]	12
AT1G09800	-0.083669501	1.387956999	-0.26154383	-0.101197484	0.089706103	-0.572350428	AT1G09800	tRNA pseudouridine synthase [Acc:F4I2L0]	12
AT1G16825	0.329855243	1.382134961	0.187000652	0.214309876	0.06974204	-0.192346443	RTNLB23	Reticulon-like protein B23 [Acc:P0C941]	12
AT5G50175	0.988510812	1.379829625	0.088906423	0	0	0	AT5G50175	Transmembrane protein [Acc:Q2V301]	12
AT1G24600	1.076222245	1.370651058	-0.287181339	-0.278000458	0.474506932	-0.68087613	AT1G24600	At1g24600 [Acc:Q9FYK4]	12
AT5G10990	0.717107474	1.363935362	0.082970104	0	0	0	AT5G10990	At5g10990 [Acc:Q9LEU2]	12
AT3G62990	0.30778088	1.357681674	0.182141394	0	0	0	AT3G62990	At3g62990 [Acc:A0JPZ2]	12
AT3G28740	0.784507396	1.353730127	0.603864765	0.517877273	1.263159917	0.767891813	CYP81D11	Cytochrome P450 81D11 [Acc:Q9LHA1]	12
AT1G07600	1.289186781	1.352235289	-0.860972484	0.209058767	0.370261116	0.020917676	MT1A	Metallothionein-like protein 1A [Acc:P43392]	12
AT1G16820	0.553536834	1.343168665	0.212605218	0.329670061	0.309087823	-0.502426215	AT1G16820	Vacuolar ATP synthase catalytic subunit-related / [Acc:F4I600]	12
AT4G12490	0.165431185	1.304839583	-0.80756112	-0.526300338	-1.578936328	-1.499654949	AT4G12490	pEARLI1-like lipid transfer protein 2 [Acc:Q9SU34]	12
AT5G01550	1.067894779	1.297288028	0.651408747	-2.95621725	0.825009602	-1.290888778	LECRKA4.2	lectin receptor kinase a4.1 [AT5G01550]	12
AT3G06355	0.086510996	1.284085073	-0.49871928	0.708371117	-0.011301452	-0.18547286	AT3G06355	None	12
AT2G47730	0.741501077	1.281938694	0.578034169	0.747112939	0.96537276	0.69193224	GSTF8	Glutathione S-transferase F8, chloroplastic [Acc:Q96266]	12
AT5G50760	0.828773653	1.279685769	0.157529405	-0.304502149	0.840469066	0.692931168	AT5G50760	At5g50760 [Acc:Q9LUE5]	12
AT1G22985	0.933955321	1.278524355	0.67811293	0.836086994	0.421848266	0.684827155	ERF069	CRF7 [Acc:A0A178W874]	12
AT1G03660	0.411611156	1.273573395	-0.185178387	-0.053457591	0.499421475	-1.989989244	AT1G03660	Ankyrin-repeat containing protein [Acc:F4I2G0]	12
AT3G50350	0.634776485	1.226122581	0.506200276	-1.702006847	-0.807950638	-1.574163528	AT3G50350	Membrane insertase, putative (DUF1685) [Acc:Q9SND2]	12
AT3G59370	0.825981177	1.224476409	-0.043206444	0	0	0	AT3G59370	At3g59370 [Acc:Q9LX34]	12
AT1G72240	0.722350026	1.213268014	0.10845962	0.392936178	0.64994297	-0.702314063	AT1G72240	Uncharacterized protein T9N14.5 [Acc:Q9C7T1]	12
AT3G22540	0.756046944	1.213176684	0.074609436	0	0	0	AT3G22540	At3g22540 [Acc:Q9LJ92]	12
AT1G66230	0.782759889	1.208777971	0.792765075	0.143021151	0.334881724	-0.450302652	MYB20	Transcription factor MYB20 [Acc:Q9C7U7]	12
AT5G64180	0.669015956	1.199857281	0.718081232	0.467717771	0.582300837	0.405854687	AT5G64180	Dbj [Acc:Q9FMG6]	12
AT1G05560	0.238051116	1.171229112	0.896027632	0.669336447	1.014060335	0.791550484	UGT1	UDP-glucosyltransferase 75B1 [AT1G05560]	12
AT1G55990	1.089473384	1.165946349	-0.372454129	0	0	0	AT1G55990	Glycine-rich protein [Acc:F4I3I2]	12
AT5G11975	0.711410479	1.165340289	-0.175038989	-0.368779714	0.144963928	-0.809187853	AT5G11975	None	12
AT1G67810	0.320763694	1.16513856	0.429049679	0.168409003	0.47548191	0.183193278	SUFE2	SufE-like protein 2, chloroplastic [Acc:Q9FXE3]	12
AT1G65520	0.607738502	1.161788064	0.25220469	-0.050658921	0.236921491	0.021907184	ECI1	Enoyl-CoA delta isomerase 1, peroxisomal [Acc:O04469]	12
AT3G50850	0.670099517	1.129256552	0.131348405	-0.628513392	-0.410773987	-1.187465496	AT3G50850	At3g50850 [Acc:Q9SVL4]	12
AT1G65980	0.764916572	1.129078873	0.739722909	0.607857062	0.966441148	0.627292944	PRXIIB	Peroxiredoxin-2B [Acc:Q9XEX2]	12
AT5G03030	0.414656591	1.126513908	0.201070745	0.154360105	0.120975514	-0.066358786	AT5G03030	Chaperone DnaJ-domain superfamily protein [AT5G03030]	12
AT1G03220	0.819744486	1.123235034	0.699965427	0.713835082	0.92460432	0.703227407	AT1G03220	Eukaryotic aspartyl protease family protein [Acc:Q9ZVS4]	12
AT1G73480	0.902907706	1.120054628	0.416807118	0.996124036	0.800366169	0.855391223	AT1G73480	Alpha/beta-Hydrolases superfamily protein [Acc:Q94AM5]	12
AT5G02220	0.559099132	1.116043693	-0.22534987	-0.125685358	0.946408803	0.401316308	SMR4	Cyclin-dependent protein kinase inhibitor SMR4 [Acc:Q1JPP8]	12
AT5G15770	-0.26806184	1.115895338	-0.370454813	0.850094538	1.197192088	0.732465526	GNA1	Glucosamine 6-phosphate N-acetyltransferase [Acc:Q9LFU9]	12
AT5G62340	0.473796382	1.115645983	0.03697771	0	0	0	AT5G62340	At5g62340 [Acc:Q9LVA5]	12
AT5G23460	0.01944767	1.11281238	0.680505689	-0.119722086	0.288928232	0.301947173	AT5G23460	Uncharacterized protein At5g23460 [Acc:Q9FHL2]	12
AT2G07671	0.151972732	1.10971372	-0.627220737	-0.001950692	0.069660686	-0.718062988	AT2G07671	ATP synthase subunit 9, mitochondrial [Acc:Q304C3]	12
AT2G35910	0.293862745	1.108103909	0.257294174	0.114886316	-0.057480647	0.152097889	ATL70	RING-H2 finger protein ATL70 [Acc:Q8RX29]	12
AT3G41768	0.052707338	1.104440175	-0.7287674	-0.533603941	-0.034940714	-0.793557804	AT3G41768	rRNA [AT3G41768]	12
AT2G37750	0.882413476	1.101242262	0.20065309	0.395567904	0.872456858	-0.243528821	AT2G37750	Expressed protein [Acc:O80943]	12
AT5G45095	0.403954629	1.098267848	0.884378024	0	0	0	AT5G45095	Putative uncharacterized protein [Acc:Q8GZ89]	12
AT2G30550	0.864258286	1.093334484	0.36316693	0.264835337	0.948935716	0.200797203	AT2G30550	Phospholipase A1-Igamma2, chloroplastic [Acc:Q3EBR6]	12
AT4G33550	0.245255539	1.087421529	-0.185627191	-0.651959693	0.449145886	-1.73871418	AT4G33550	At4g33550 [Acc:Q6NLG8]	12
AT4G20000	0.964114703	1.080739456	0.25657182	-0.341294971	0.478042852	-0.187407204	AT4G20000	At4g20000 [Acc:O49427]	12
AT3G08690	0.785959187	1.079456068	0.757991034	0.383270786	0.933116956	0.569922759	UBC11	Ubiquitin-conjugating enzyme E2 11 [Acc:P35134]	12
AT3G01430	0.765033336	1.075171083	0.489101306	0.320668255	0.437307594	0.103352413	AT3G01430	NHL domain protein [Acc:Q9SGH5]	12
AT1G07130	0.534737441	1.064705142	0.112805769	-0.089928619	0.358090566	-0.845578721	STN1	CST complex subunit STN1 [Acc:Q9LMK5]	12
AT2G41380	0.772659941	1.061622871	0.076869002	-0.098659755	0.14952764	-0.364583877	AT2G41380	At2g41380 [Acc:Q9ZVC3]	12
AT5G66985	-0.182471954	1.060896433	-0.176783335	-2.070871107	-0.374612554	-1.60488531	AT5G66985	At5g66985 [Acc:Q8L9T9]	12
AT2G01520	0.906298045	1.050026536	0.691142885	0.431376654	1.40966756	1.342586957	MLP328	MLP-like protein 328 [Acc:Q9ZVF3]	12
AT5G16060	0.274005312	1.048663114	-0.184440028	0.152563553	0.251052795	-0.289349126	AT5G16060	COX assembly mitochondrial protein [Acc:A0A178UR08]	12
AT5G22890	0.660868359	1.047950743	0.557211373	-0.895187004	0.74909791	1.017725732	STOP2	Protein SENSITIVE TO PROTON RHIZOTOXICITY 2 [Acc:Q0WT24]	12
AT5G06330	0.788618115	1.046302967	0.715793291	0	0	0	AT5G06330	At5g06330 [Acc:Q9FNH5]	12
AT4G00170	-0.032793151	1.04313953	-0.325116323	0.104688747	0.231449382	0.296028542	PVA13	Vesicle-associated protein 1-3 [Acc:Q84WW5]	12
AT5G03545	0.2940879	1.0427045	0.518734215	-0.15296677	0.188768135	-0.907565823	AT4	At5g03545 [Acc:Q9LZD2]	12
AT3G47836	0.180966807	1.041850111	-0.282292615	0.23342445	0.374684138	-0.446278506	AT3G47836	Transmembrane protein [Acc:Q3EAN0]	12
AT5G15802	0.467323202	1.034680836	0.213425363	0.111616966	0.256038769	-0.195153989	AT5G15802	Chaperone [Acc:Q8GYN2]	12
AT3G25882	0.638433659	1.031354437	-0.663044636	1.563490833	1.380053065	1.342640147	NIMIN-2	NIMIN-2 [Acc:A0A178VAB1]	12
AT4G37320	0.691785383	1.030933589	0.493335683	0.466413978	0.384585036	0.279588275	CYP81D5	Cytochrome P450, family 81, subfamily D, polypeptide 5 [Acc:O23156]	12
AT4G27030	0.772062284	1.029462403	0.597897183	-0.411805974	0.127781319	-0.093213169	FAD4	FADA [Acc:A0A178UUY9]	12
AT2G27660	0.913939133	1.02866579	0.372880424	-1.382323699	-0.160266711	0.218550357	AT2G27660	Cysteine/Histidine-rich C1 domain family protein [Acc:Q9ZUW8]	12
AT5G01250	0.65205322	1.027931091	0.499578885	0	0	0	AT5G01250	Alpha 1,4-glycosyltransferase family protein [Acc:Q9LFB1]	12
AT4G09695	0.696087673	1.027859359	0.51541623	-0.233991939	-0.239891212	-0.412780445	AT4G09695	None	12
AT1G05300	0.74281734	1.022597424	0.294903259	-0.666561596	-0.105613739	-0.781607084	ZIP5	Zinc transporter 5 [Acc:O23039]	12
AT5G23220	0.869788835	1.015470918	0.086783222	0	0	0	NIC3	Nicotinamidase 3 [Acc:Q9FMX8]	12
AT1G05210	-0.062835169	1.014898539	-0.503244959	-0.025354718	-0.354932891	-0.46494085	AT1G05210	At1g05210 [Acc:O23047]	12
AT3G16240	0.5769595	1.01483674	0.482485303	0.339962628	0.56759029	0.27184531	TIP2-1	Aquaporin TIP2-1 [Acc:Q41951]	12
AT5G08330	0.338168286	1.013128039	0.355025873	0.178822491	0.272487172	0.197773938	TCP21	Transcription factor TCP21 [Acc:Q9FTA2]	12
AT1G77120	0.162385224	1.011650731	0.398500154	-1.087706017	0.365274826	-0.373217413	ADH1	Alcohol dehydrogenase class-P [Acc:P06525]	12
AT5G18790	0.135281822	1.005642313	-0.044575591	-0.446840119	-0.010152952	-0.585317439	AT5G18790	At3g06320 [Acc:Q9SQT5]	12
AT5G51810	0.347196333	0.676587954	0.881817667	1.23803512	-0.059855126	1.944762764	GA20OX2	Gibberellin 20 oxidase 2 [Acc:Q39111]	13
AT1G18300	0.078765666	-0.721223741	-0.13268422	0.639369565	0.814202236	1.94190548	NUDT4	Nudix hydrolase 4 [Acc:Q9LE73]	13
AT5G01100	0.440243844	0.069207579	0.53929051	0.979412312	-0.496324644	1.792733746	FRB1	FRB1 [Acc:A0A178U817]	13
AT4G19230	0.085402363	0.094814927	-0.162121233	0.667513837	-0.329959074	1.713356383	CYP707A1	Cytochrome P450, family 707, subfamily A, polypeptide 1 [Acc:A8MRX5]	13
AT2G17840	0.28196493	-0.016154777	-0.336265327	0.340459257	-1.786440708	1.561140127	ERD7	Protein EARLY-RESPONSIVE TO DEHYDRATION 7, chloroplastic	13
AT5G00980	0.300777438	0.573706342	0.663063547	0.477853962	-0.240607454	1.551534588	AT5G00980	None	13
AT5G24530	0.812908681	0.604891803	0.78595994	0.451011127	-0.168356285	1.495969398	DMR6	Protein DOWNY MILDEW RESISTANCE 6 [Acc:Q9FLV0]	13
AT1G75590	0.191890128	0.363673489	0.132122802	1.474659355	-1.720122352	1.494897495	AT1G75590	SAUR-like auxin-responsive protein family [Acc:F4HZ54]	13
AT4G26990	0.320290173	0.760922709	0.235408583	1.001163253	-0.20509577	1.453829714	AT4G26990	AT4g26990/F10M23_330 [Acc:Q8RY61]	13
AT4G16680	-0.055978726	-0.408357022	0.117694873	0.515148462	-0.332827581	1.353952172	AT4G16680	P-loop containing nucleoside triphosphate hydrolases superfamily	13
AT1G73540	0.23473109	0.072280704	0.153898499	0.707939111	-0.864782596	1.328365963	NUDT21	Nudix hydrolase 21, chloroplastic [Acc:Q8VY81]	13
AT5G51670	0.261600382	0.416265763	0.604170179	0.641418588	-0.136329987	1.31463999	AT5G51670	Similarity to unknown protein [Acc:Q9LTD7]	13
AT5G18780	0.449874312	-0.278660098	0.785496524	0.702127239	-0.137432042	1.203210102	AT5G18780	FBD-associated F-box protein At5g18780 [Acc:Q56YH2]	13
AT1G18740	0.038326294	0.171260693	-0.086059361	0.379854011	0.793269287	1.196598091	AT1G18740	At1g18740/F6A14_15 [Acc:Q9M9U5]	13
AT3G49645	0.222595108	0.116565659	0.903488001	0.776975463	-0.370101861	1.193859474	AT3G49645	FAD-binding protein [Acc:A0A1I9LMB9]	13
AT3G13470	0.881327125	0.956058681	0.906753602	0.960424018	0.668176662	1.160433406	CPN60B2	Chaperonin 60 subunit beta 2, chloroplastic [Acc:Q9LJE4]	13
AT3G27210	0.121476655	-0.157483849	-0.022379169	0.348675855	-0.541146202	1.15048816	Y-2	Uncharacterized protein At3g27210 [Acc:Q9LK32]	13
AT4G11280	-0.043801168	-0.236457925	0.245113461	0.824788246	-0.388160261	1.140039801	ACS6	1-aminocyclopropane-1-carboxylate synthase 6 [Acc:Q9SAR0]	13
AT3G13224	0.509071054	0.251218431	0.753048422	0.821528824	-0.745517294	1.135888822	AT3G13224	RNA-binding (RRM/RBD/RNP motifs) family protein [Acc:Q9LHL2]	13
AT2G30020	0.013906408	-0.037361396	-0.159896118	0.288812473	0.853865631	1.132230244	AT2G30020	PP2C-type phosphatase AP2C1 [Acc:F6LPR5]	13
AT1G31814	0.311115095	-0.058223481	0.06341777	0.786142587	0.918791173	1.131833886	FRL2	Inactive FRIGIDA-like protein 2 [Acc:Q9C6S2]	13
AT4G16000	-0.463201314	-0.363895757	0.235022571	0.536053783	-0.420377021	1.111446633	AT4G16000	Uncharacterized protein At4g16000 [Acc:Q0WWC4]	13
AT4G37590	0.182949772	-0.035624627	0.378557935	0.958114239	-0.380639144	1.108637024	NPY5	BTB/POZ domain-containing protein NPY5 [Acc:Q0WL52]	13
AT3G19240	0.244328624	0.270773371	0.293933612	0.543727602	-1.316238895	1.106832652	AT3G19240	Dem protein [Acc:Q9LJK5]	13
AT1G03470	0.075536534	-0.280305736	0.455251321	0.979635538	0.719475618	1.10202582	NET3A	NET3A [Acc:A0A178W5C1]	13
AT5G47070	-0.26125612	-0.491246261	0.299480557	0.916511143	0.074278346	1.082014772	PBL19	Probable serine/threonine-protein kinase PBL19 [Acc:Q9LTC0]	13
AT3G19970	-0.620730934	-0.014951451	-0.184167585	0.394529709	0.854959311	1.080434215	AT3G19970	AT3g19970/MZE19_2 [Acc:Q9LHE8]	13
AT5G65830	-0.064012202	-0.273270705	-0.236867388	0.515816939	-0.33230243	1.066652156	ATRLP57	Receptor-like protein 57 [Acc:Q9FH86]	13
AT1G74450	-0.032232566	0.142666914	-0.598398027	0.186340485	-0.556656964	1.063524629	AT1G74450	At1g74450/F1M20_13 [Acc:Q9CA69]	13
AT1G27410	0.117329427	-0.11066975	0.774600741	0.606197388	0.993839723	1.061732089	AT1G27410	DNA repair metallo-beta-lactamase family protein [Acc:Q9FZJ4]	13
AT3G55840	0.497517171	0.306281537	0.20463249	0.645724472	-0.563367699	1.036161298	HSPRO1	Nematode resistance protein-like HSPRO1 [Acc:Q9LY61]	13
AT3G01100	0.633700108	0.44298807	0.976432133	0.503240032	-0.801944193	1.033384336	HYP1	hypothetical protein 1 [AT3G01100]	13
AT1G43770	0.26308406	0.020211925	0.422237091	0.214155749	-0.736267293	1.031618894	AT1G43770	RING/FYVE/PHD zinc finger superfamily protein [Acc:B3H578]	13
AT4G11840	0.286260622	-0.400790991	0.617505185	0.804081846	-0.294409326	1.030013484	PLDGAMMA3	Phospholipase D gamma 3 [Acc:Q9T052]	13
AT3G07770	0.250326898	0.612231926	0.152967924	0.748504162	-0.633429961	1.028994277	HSP90-6	Heat shock protein 90-6, mitochondrial [Acc:F4JFN3]	13
AT1G72070	0.372058628	-0.19993189	0.798895385	1.064992547	-1.021363071	1.028369279	AT1G72070	Chaperone DnaJ-domain superfamily protein [Acc:F4IBN6]	13
AT2G27500	0.120533959	0.21463939	0.289719947	0.758739761	0.808759008	1.028281408	AT2G27500	Glucan endo-1,3-beta-glucosidase 14 [Acc:Q9ZQG9]	13
AT3G03310	0.262455162	0.286418214	0.686207734	0.54725577	-0.489613618	1.018159729	LCAT3	Phospholipase A(1) LCAT3 [Acc:Q93V61]	13
AT1G04390	0.086874356	-0.513269018	0.710020122	0.429469128	-0.755003464	1.008280954	AT1G04390	BTB/POZ domain-containing protein At1g04390 [Acc:P93820]	13
AT3G10720	0.24383142	0.563122532	-0.674575863	0.90908781	-0.534161698	1.007227413	PME25	Probable pectinesterase/pectinesterase inhibitor 25 [Acc:Q94CB1]	13
AT1G20440	1.09357891	0.79925533	0.4944244	0.755348998	1.495344594	1.280942808	COR47	RD17 [Acc:A0A384KK16]	14
AT3G01210	0.996717492	0.982385908	1.213242775	0.715271021	1.030870328	1.192757804	AT3G01210	RNA-binding (RRM/RBD/RNP motifs) family protein [Acc:Q9MAD0]	14
AT3G16800	0.960854938	0.823997002	1.034040709	0.670619774	1.102211354	1.031689154	AT3G16800	Probable protein phosphatase 2C 41 [Acc:Q9LRZ4]	14
AT4G30290	0.029522068	0.320505944	-0.619902337	1.325375443	2.757173589	2.346474787	XTH19	Xyloglucan endotransglucosylase/hydrolase [Acc:A0A178UU22]	14
AT4G01950	0.301873001	0.099985935	-0.278365098	0.902551037	1.227953996	1.642088667	ATGPAT3	glycerol-3-phosphate acyltransferase 3 [AT4G01950]	14
AT5G58770	0.608429875	0.676452074	0.369784088	0.997475413	1.878700494	1.530753544	AT5G58770	Dehydrodolichyl diphosphate synthase 2 [Acc:Q56Y11]	14
AT3G30180	0.214315111	-0.069200513	0.343013015	0.790432915	1.373134536	1.462580845	CYP85A2	Cytochrome P450 85A2 [Acc:Q940V4]	14
AT3G13600	0.230369171	0.286725252	-0.127341016	0.970689611	1.59853473	1.447300956	IQM2	Calmodulin-binding family protein [Acc:A0A178VF87]	14
AT4G01680	0.614377459	0.319456833	0.006965336	1.230818783	1.977174377	1.439292021	MYB55	Myb domain protein 55 [Acc:Q9ZSI4]	14
AT3G29575	0.61771862	0.323497103	-0.200807523	1.045579001	1.510334186	1.373790929	AFP3	Ninja-family protein AFP3 [Acc:Q94F39]	14
AT3G54000	0.213254769	-0.211440132	-0.31011207	0.977266802	1.27557936	1.371108801	AT3G54000	TIP41-like protein [Acc:Q84JE0]	14
AT5G07010	-0.742832974	-0.593584277	-0.356761496	0.919646607	1.768836491	1.368152302	SOT15	Sulfotransferase [Acc:A0A178UG65]	14
AT1G64640	0.1470488	0.618595515	0.454507287	0.994772532	1.646257742	1.367677565	ENODL8	ENODL8 [Acc:A0A178WAZ0]	14
AT1G20450	0.873083619	0.66500541	0.080527968	1.206201094	1.737418252	1.335205807	ERD10	Dehydrin ERD10 [Acc:P42759]	14

AT5G52050	-1.242210825	-0.367319904	-0.719894371	1.013602884	1.630504664	1.330349833	DTX50	Protein DETOXIFICATION 50 [Acc:Q9FJ87]	14
AT2G41640	0.499099772	0.672606021	0.477990227	0.626424944	1.122944965	1.322600217	AT2G41640	At2g41640/T32G6.16 [Acc:O22225]	14
AT3G50260	0.039379098	0.69857157	-0.011822283	0.898019315	1.424321733	1.261880125	ERF011	Ethylene-responsive transcription factor ERF011 [Acc:Q9SNE1]	14
AT2G21940	0.116784036	0.072060155	0.277524305	0.754827966	1.119000601	1.261568936	SK1	Shikimate kinase 1 [Acc:F4IIJ2]	14
AT3G19580	0.544322734	0.907611487	0.40185911	1.142936653	1.226086445	1.237269347	AZF2	ZF2 [Acc:A0A178VJ97]	14
AT5G64510	0.652170111	0.947786197	0.182548835	0.84155031	1.498619637	1.234432968	TIN1	Tunicamycin induced protein [Acc:Q84JN2]	14
AT3G15540	0.339048953	0.338234301	0.083653671	0.990681082	1.217890778	1.208854152	IAA19	Auxin-responsive protein [Acc:Q2VWA2]	14
AT3G12050	0.18876081	0.428142959	0.147520799	0.899990606	1.061606211	1.122999866	AT3G12050	Aha1 domain-containing protein [Acc:Q9LHL7]	14
AT3G03520	0.042182415	-0.267508836	0.388838407	0.83982262	1.556513085	1.119863724	NPC3	NPC3 [Acc:A0A178VBD6]	14
AT4G01410	0.088493479	0.537675436	-0.813201736	0.614522068	1.209082119	1.099813542	AT4G01410	At4g01410 [Acc:Q9M132]	14
AT1G49500	0.290385246	-0.237625662	0.605476277	0.807511066	1.206503475	1.0610744	AT1G49500	At1g49500/F13F21_6 [Acc:Q9XIB7]	14
AT1G04130	0.19729959	0.062794129	0.142875936	0.856665894	1.055857451	1.045935238	TPR2	Tetratricopeptide repeat (TPR)-like superfamily protein [Acc:F4I456]	14
AT5G58590	0.408485687	0.626872945	0.171275355	0.983754641	1.583256021	1.039726453	RANBP1C	At5g58590 [Acc:Q1WWI2]	14
AT5G28300	0.210857477	0.611564713	0.398441641	0.793451182	1.020821748	1.038385339	AT5G28300	Trihelix transcription factor GTL2 [Acc:Q8H181]	14
AT5G25190	0.266294798	0.518067682	0.508821156	1.260636182	1.569078755	1.028974601	ERF003	Ethylene-responsive transcription factor ERF003 [Acc:Q94AW5]	14
AT3G14590	0.621203178	-0.091081127	0.575336949	1.033283085	1.018917567	1.006079026	NTMC2TYPE6.2	Calcium-dependent lipid-binding (CaLB domain) family [Acc:B3H4I1]	14
AT1G09250	0.342093259	0.426779822	-0.497146959	0.802782501	1.33188697	1.001107134	BHLH149	Transcription factor bHLH149 [Acc:O80482]	14
AT5G56985	1.439937071	1.367079616	0.411495257	1.311920215	1.555215714	0.896343115	AT5G56985	None	15
AT5G64230	1.039307326	1.511402709	0.912386183	0.66692825	1.170668351	0.754370687	AT5G64230	unknown protein	15
AT2G19310	0.891837431	1.395093711	0.612480471	1.007581274	1.341685522	0.970855558	HSP18.5	18.5 kDa class IV heat shock protein [Acc:O64564]	15
AT4G39235	0.969103969	1.600696073	0.508469019	0.991394384	1.424868985	0.938271476	AT4G39235	At4g39235 [Acc:Q29Q00]	15
AT4G02980	0.860804295	1.084307473	0.877280486	0.847648219	1.349690676	0.833996108	ERABP1	Auxin-binding protein 1 [Acc:P33487]	15
AT2G33740	0.850502254	1.30450889	0.754980416	0.76931869	1.09578289	0.722290171	CUTA	Protein CutA, chloroplastic [Acc:P93009]	15
AT1G14980	0.724116675	1.382637912	0.003282831	0.748264894	1.139310189	0.408268941	CPN10	10 kDa chaperonin, mitochondrial [Acc:P34893]	15
AT1G04980	0.925210723	1.093017772	0.242779693	0.548229778	1.035847151	0.636788483	PDIL2-2	Protein disulfide-isomerase like 2-2 [Acc:Q9MAU6]	15
AT4G33666	0.063405052	1.262166486	-0.022587926	0.490850048	1.177218165	0.855346924	AT4G33666	Uncharacterized protein At4g33666 [Acc:Q94AJ7]	15
AT4G33660	0.799315068	1.572223651	1.070382611	0.478619019	1.03928872	0.7509046	AT4G33660	Adhesive/proline-rich-like protein [Acc:Q8LG30]	15
AT2G36830	0.886308163	1.140070197	0.625712434	0.441186476	1.177228725	0.646269063	TIP1-1	Aquaporin TIP1-1 [Acc:P25818]	15
AT5G12020	4.216717916	5.042931016	4.284589917	2.80768607	3.865039709	3.740363605	HSP17.6	17.6 kDa class II heat shock protein	16
AT3G07365	4.165475298	4.255887763	4.494544608	3.201843158	4.253090273	5.277760885	AT3G07365	None	16
AT1G53540	4.061489808	4.917347565	4.324765453	3.209090685	4.453172178	4.330712326	HSP17.6C	17.6 kDa class I heat shock protein 3	16
AT3G46230	3.900891361	4.595675571	3.832939339	3.079545851	4.434544341	3.924194209	HSP17.4A	17.4 kDa class I heat shock protein	16
AT4G25200	3.288786027	4.258889866	2.660263978	2.661418061	3.177234944	2.566494173	HSP23.6	HSP23.6-MITO	16
AT2G29500	3.093928203	3.587850616	2.839217862	2.849649952	3.961707296	3.437805374	HSP17.6B	17.6 kDa class I heat shock protein 2	16
AT1G72660	2.930664895	3.19514782	2.893987552	3.279768225	4.184636731	4.22886081	DRG2	Developmentally-regulated G-protein 2 [Acc:Q9CAI1]	16
AT3G09640	2.777945463	3.335523792	3.223333959	5.268203922	5.943868128	5.959524904	APX2	APX2 [Acc:A0A178VH43]	16
AT1G07400	2.72912522	3.078602642	2.152146182	1.342733437	2.005126572	1.571054824	HSP17.8	17.8 kDa class I heat shock protein [Acc:Q9LNW0]	16
AT5G48570	2.629963397	2.487406905	2.283606412	2.026393277	2.486236769	2.336482236	FKBP65	Peptidyl-prolyl cis-trans isomerase FKBP65 [Acc:Q9FJL3]	16
AT4G21320	2.389170399	2.60126434	2.765458609	2.57367465	3.072907353	3.143746964	HSA32	HSA32 [Acc:A0A178UV51]	16
AT3G53830	2.006511535	1.421121501	2.267395726	1.483392926	1.644589821	1.855468035	AT3G53830	Regulator of chromosome condensation (RCC1) family protein	16
AT2G47180	1.714636554	1.577774387	1.826593257	1.604718022	2.390303124	1.809239127	GOLS1	Galactinol synthase 1 [Acc:O22893]	16
AT3G24100	1.522007958	1.611206999	1.30397193	1.158339487	1.531791991	1.265851053	AT3G24100	Putative uncharacterized protein AT3g24100 [Acc:Q94K36]	16
AT5G12110	1.408043486	1.770589217	1.480163382	1.719297034	2.151240198	1.871159637	AT5G12110	At5g12110 [Acc:Q29PY2]	16
AT1G09140	1.3482563	1.267760953	1.336809448	1.39649929	1.723402137	1.737851845	SR30	Serine/arginine-rich splicing factor SR30 [Acc:Q9XFR5]	16
AT5G59720	1.247581157	2.34974104	2.443903287	1.865896046	3.1055698	3.101612335	HSP18.1	18.1 kDa class I heat shock protein [Acc:P19037]	16
AT2G17900	1.247011039	1.15389472	1.396939602	1.018768602	1.256603947	1.583600186	SDG37	SET domain group 37 [AT2G17900]	16
AT1G17870	1.23485232	1.246556652	1.577568389	1.20712569	2.143686865	2.314381863	EGY3	Probable zinc metallopeptidase EGY3, chloroplastic [Acc:Q9LMU1]	16
AT4G12400	1.178511755	1.037800567	1.105736817	1.806496271	2.362118136	2.394235056	HOP3	Hsp70-Hsp90 organizing protein 3 [Acc:Q9STH1]	16
AT5G47830	1.045100032	1.492456533	1.001849386	1.212580826	1.220113063	1.096054416	AT5G47830	unknown protein; Ha. [AT5G47830]	16
AT5G12030	3.523843547	4.419820501	3.622538185	1.878379372	2.836665884	2.438882933	HSP17.7	17.7 kDa class II heat shock protein [Acc:O81822]	16
AT4G27670	3.044988893	4.486063991	3.578685142	1.193172796	3.605151079	3.902706522	HSP21	HSP21 [Acc:A0A178UVU5]	16
AT4G10250	2.743393981	3.932565334	3.207527448	1.974340973	3.427122871	3.132515077	HSP22.0	22.0 kDa heat shock protein [Acc:Q38806]	16
AT1G05340	1.307937815	1.39275365	1.024712046	0.989304888	1.747794783	1.311923965	AT1G05340	Cysteine-rich TM module stress tolerance protein [Acc:O23035]	16
AT1G52560	1.759724025	2.571227196	1.98679889	1.854189094	2.82737858	1.149323239	HSP26.5	26.5 kDa heat shock protein, mitochondrial [Acc:Q9SSQ8]	16
AT4G23680	1.317619843	1.130265982	0.94062238	0.784658588	2.184290143	1.070711775	AT4G23680	AT4g23680/F9D16_150 [Acc:Q9SUQ9]	16
AT2G30480	1.187749571	0.612212991	1.711738661	1.717359588	1.68918519	1.943191579	AT2G30480	unknown protein; Ha. [AT2G30480]	16
AT5G37340	1.064659059	0.870766985	1.057340995	1.090186431	1.217677066	1.414475052	AT5G37340	ZPR1 zinc-finger domain protein [Acc:F4K746]	16
AT2G32120	0.998669468	1.317332767	1.352457495	1.001810447	1.505674139	1.375587608	HSP70-8	Heat shock 70 kDa protein 8 [Acc:Q9SKY8]	16
AT3G03780	-1.334833635	-1.138052547	-1.358276212	-1.274794802	-0.619364049	-0.945840822	MS2	5-methyltetrahydropteroyltriglutamate--homocysteine methyltransferase 2	17
AT3G30775	-1.115233953	-1.125538765	-1.170140669	-0.70925954	-0.98262808	-1.383088327	POX1	Proline dehydrogenase 1, mitochondrial [Acc:P92983]	17
AT4G11660	-1.580803989	-1.72031658	-2.119695159	-0.995211758	-0.650295224	-1.554585563	HSFB2B	Heat stress transcription factor B-2b [Acc:Q9T0D3]	17
AT3G51240	-1.118586925	-1.269922063	-1.735583121	-0.886897072	-0.471349833	-0.588906284	F3H	Naringenin,2-oxoglutarate 3-dioxygenase [Acc:Q9S818]	17
AT5G41080	-1.27182	-1.026614509	-1.881202323	0.039211786	-0.962940255	-0.006615936	GDPD2	Glycerophosphodiester phosphodiesterase GDPD2 [Acc:Q9FLM1]	17
AT2G19800	-1.416281446	-1.78249059	-1.184143957	-0.803375003	-1.460941203	-1.088003289	MIOX2	At2g19800 [Acc:B4F7Q2]	17
AT2G01150	-1.499880939	-1.158494968	-1.534765083	0.736236118	0.309087001	0.086694172	RHA2B	RING-H2 finger protein 2B [AT2G01150]	17
AT5G48070	-1.626476862	-1.354151108	-2.265611764	0	0	0	XTH20	Xyloglucan endotransglucosylase/hydrolase protein 20 [Acc:Q9FI31]	17
AT1G77530	-1.920376955	-2.037929235	-1.729753106	0	0	0	AT1G77530	O-methyltransferase family protein [Acc:Q9CAQ3]	17
AT1G77520	-2.30949384	-1.554438606	-2.008604937	0	0	0	AT1G77520	O-methyltransferase family protein [Acc:Q9CAQ4]	17
AT3G01260	-2.504099636	-2.275703173	-4.018824892	0	0	0	AT3G01260	Galactose mutarotase-like superfamily protein [Acc:F4J5Z7]	17
AT3G63350	-2.925820507	-1.867085748	-2.792690622	-1.093227377	-2.547609387	-3.451653508	HSFA7B	Heat stress transcription factor A-7b [Acc:Q9M1V5]	17
AT5G62020	-1.018929694	-0.914066663	-1.404082749	0.330695419	-0.316095309	-0.387654684	HSFB2A	HSFB2A [Acc:A0A384KDZ5]	17
AT5G23020	-1.198341922	-0.857996831	-1.68134366	-1.009730991	0.504135253	0.38458386	MAM3	Methylthioalkylmalate synthase 3, chloroplastic [Acc:Q9FN52]	17
AT5G59820	-1.205175705	-0.501604197	-1.401091102	-0.002467346	-0.112855001	-0.028720574	ZAT12	Zinc finger protein ZAT12 [Acc:Q42410]	17
AT5G52020	-1.274135878	-0.372131626	-1.612360591	-0.988483458	1.308909578	1.906222977	ERF025	Ethylene-responsive transcription factor ERF025 [Acc:Q9FJ90]	17
AT2G03090	-1.284783404	-0.797695854	-1.205445285	0.322985391	0.651659629	0.384999826	EXPA15	Expansin-A15 [Acc:O80622]	17
AT4G16370	-1.414452851	-0.469594137	-1.59498894	-0.603301894	-0.585097573	-0.538996273	ATOPT3	oligopeptide transporter [AT4G16370]	17
AT1G27140	-1.421020788	-0.384964707	-2.174562217	0	0	0	GSTU14	GSTU14 [Acc:A0A178WN03]	17
AT3G58990	-1.624696542	-0.772084681	-1.805891632	-1.273206359	0.026997098	-0.809240368	IPMI1	3-isopropylmalate dehydratase small subunit 2 [Acc:Q9LYT7]	17
AT1G05700	-1.733813268	-0.411822827	-1.542920361	-6.415561018	-0.482684874	-1.831646891	AT1G05700	Leucine-rich repeat transmembrane protein kinase protein [AT1G05700]	17
AT5G59680	-2.172714716	-0.800144123	-1.857364241	0	0	0	AT5G59680	Probable LRR receptor-like serine/threonine-protein kinase [Acc:Q9FN93]	17
AT2G32487	-2.872856586	-1.269446905	-2.33470248	-0.849222751	0.292972616	-0.731378046	AT2G32487	unknown protein	17
AT4G20970	-2.984233363	-1.840312552	-4.20841951	-2.646070562	-1.478889502	-3.2692883	BHLH162	Transcription factor bHLH162 [Acc:F4JIJ7]	17
AT1G26240	-3.579884297	-1.120444749	-3.753696791	0	0	0	AT1G26240	Proline-rich extensin-like family protein [Acc:Q9C668]	17
AT4G21400	-0.726941394	-2.628019252	-1.035961117	-0.43097371	-1.030863848	-0.565360492	CRK28	Cysteine-rich receptor-like protein kinase 28 [Acc:O65405]	18
AT2G40150	-0.202737441	-1.007904612	0.216068621	-0.29187846	0.393383565	0.245465775	TBL28	Protein trichome birefringence-like 28 [Acc:Q94K00]	18
AT3G15220	-0.284780878	-1.010600462	0.206491469	0.084499916	-0.195512966	0.249471483	AT3G15220	MAP kinase [Acc:Q9LDN6]	18
AT2G01130	-0.546206474	-1.010659965	-0.029678651	-0.475232265	-0.580365045	-0.162123583	AT2G01130	DExH-box ATP-dependent RNA helicase DExH5, mitochondrial [Acc:F4IM84]	18
AT3G15550	-0.748536395	-1.0161903	-0.320404131	-0.153867847	-0.084028245	-0.128122857	AT3G15550	Trichohyalin [Acc:Q9LRP9]	18
AT2G20440	-0.209667024	-1.021601923	-0.080604734	-0.561275533	0.262029524	0.167396853	AT2G20440	Ypt/Rab-GAP domain of gyp1p superfamily protein [Acc:F4IVD6]	18
AT4G15563	0.323062135	-1.027664647	0.052578449	-0.033345435	-0.017056404	0.684617753	AT4G15563	unknown protein; Ha. [AT4G15563]	18
AT3G51740	-0.728313935	-1.028824572	0.148835508	-0.230199702	-0.357549491	-0.29132202	IMK2	inactive leucine-rich repeat receptor-like protein kinase IMK2 [Acc:Q9SCT4]	18
AT2G32440	-0.470050247	-1.031433707	-0.150828837	0.055102386	-0.389604741	0.177962773	KAO2	Ent-kaurenoic acid oxidase 2 [Acc:Q9C5Y2]	18
AT3G51480	-0.286604838	-1.040305546	0.11781625	0.168849141	-0.122364643	-0.25239391	GLR3.6	Glutamate receptor 3.6 [Acc:Q84W41]	18
AT3G52340	-0.771463675	-1.041043092	-0.694576711	0.041131798	0.098793414	0.177601179	SPP2	Sucrose-6F-phosphate phosphohydrolase 2 [Acc:A0A1I9LQG1]	18
AT4G16957	0.035659974	-1.042397583	0.218764518	0.034360816	-0.032995745	-0.230911877	AT4G16957	SUPPRESSOR OF, CONSTITUTIVE protein [Acc:A0A1P8B3D3]	18
AT3G25820	-0.254729409	-1.045341664	-0.332352845	1.040598216	0.87686994	1.561724586	TPS27	1,8-cineole synthase 2, chloroplastic [Acc:P0DI77]	18
AT3G13730	-0.916598179	-1.060679494	-0.647699693	-0.048555152	-0.200964198	0.466656751	CYP90D1	CYP90D1 [Acc:A0A178VDZ8]	18
AT4G00520	-0.275470115	-1.064694249	-0.018558135	-0.672578716	-0.335829759	-0.567553476	AT4G00520	Acyl-CoA thioesterase family protein [AT4G00520]	18
AT2G29060	-0.400228139	-1.0683224	0.045361868	-0.287696706	-0.627368675	-0.625958896	SCL33	Scarecrow-like protein 33 [Acc:P0C883]	18
AT1G30820	-0.862518961	-1.073627483	-0.6726225	-0.118298765	-0.596824194	-0.118469345	AT1G30820	CTP synthase [Acc:F4I6G9]	18
AT3G11260	-1.186641738	-1.077941637	-1.030661023	-0.368295208	0.029743542	-1.123532224	WOX5	WUSCHEL-related homeobox 5 [Acc:Q8H1D2]	18
AT1G10570	-0.41947363	-1.07887149	-0.10287017	-0.334158358	-0.54945782	-0.333038786	ULP1C	Ubiquitin-like-specific protease 1C [Acc:Q8RWN0]	18
AT2G23030	0.068040161	-1.081557024	0.151201493	0.363258506	-0.894803376	-0.326582561	SRK2J	SNRK2.9 [Acc:A0A178VSC8]	18
AT1G50240	-0.857427864	-1.081842945	-0.254636708	-0.684193196	-0.968004839	-0.861730651	TIO	Serine/threonine-protein kinase TIO [Acc:Q2QAV0]	18
AT4G33260	-0.4577264	-1.09545282	0.374733455	0.015608805	-0.337586225	-0.116458596	CDC20-2	Cell division cycle 20.2, cofactor of APC complex [Acc:Q9S7I8]	18
AT2G18000	-0.199556485	-1.103964737	-0.491554726	0.446019346	0.819747424	-0.191374308	TAF14	Transcription initiation factor TFIID subunit 14 [Acc:F4IPK2]	18
AT2G18360	-0.490256765	-1.107671002	-0.328579058	-0.300646483	-0.207621225	-0.68532371	AT2G18360	Alpha/beta-Hydrolases superfamily protein [Acc:Q9ASW5]	18
AT1G18270	-0.466025673	-1.112161384	-0.280043458	-0.287324922	-0.52964094	-0.099724664	AT1G18270	Ketose-bisphosphate aldolase class-II family protein [Acc:F4IAP5]	18
AT3G44670	-0.356490659	-1.115511131	0.58468263	-0.01176973	-0.302906635	0.189624666	AT3G44670	Disease resistance protein (TIR-NBS-LRR class) family [Acc:F4J361]	18
AT5G40840	-0.199176922	-1.121697662	-0.363038124	-0.223288008	-0.4846961	-0.03776939	SYN2	Sister chromatid cohesion 1 protein 2 [Acc:Q9FQ20]	18
AT3G03300	-0.154422806	-1.124883325	0.455795133	-0.166059327	-0.42505797	0.242448997	DCL2	Endoribonuclease Dicer homolog 2 [Acc:Q3EBC8]	18
AT1G21650	-0.394533902	-1.12732418	-0.356204699	-0.702898545	-0.848759726	-0.108897336	SECA2	Protein translocase subunit SecA [Acc:F4HY36]	18
AT2G33770	-0.677867389	-1.130229247	0.202893944	-0.318285154	-0.215249334	-0.232418069	UBC24	Probable ubiquitin-conjugating enzyme E2 24 [Acc:Q8VY10]	18
AT5G63160	-0.56987563	-1.146043058	-0.343426553	0.64764499	0.034558114	0.666733061	BT1	BTB/POZ and TAZ domain-containing protein 1 [Acc:Q9FMK7]	18
AT1G77380	-0.920911108	-1.14859018	-0.96094532	0.29614358	0.570958748	0.587763851	AAP3	ATAAP3 [Acc:A0A178W3U1]	18
AT3G48360	-0.174870391	-1.153122387	0.20155516	0.924266902	0.305666221	0.783743238	BT2	BTB/POZ and TAZ domain-containing protein 2 [Acc:Q94BN0]	18
AT1G34670	-0.104031706	-1.153528181	-0.889644991	0	0	0	AtMYB93	Transcription factor MYB93 [Acc:Q9S9Z2]	18
AT1G49960	-0.182489252	-1.167947767	-0.95409261	0	0	0	NAT4	Nucleobase-ascorbate transporter 4 [Acc:P93039]	18
AT3G18400	-0.280124814	-1.168752457	-0.694977678	0	0	0	anac058	NAC domain containing protein 58 [Acc:Q9LS50]	18
AT5G65460	-0.501784488	-1.171914504	0.014521922	-0.410437145	-0.613812167	-0.291465612	KCA2	kinesin like protein for actin based chloroplast movement 2 [AT5G65460]	18
AT1G26260	-0.633971796	-1.220824058	-0.825437321	-0.436427353	-0.714743123	-0.389591977	BHLH76	Transcription factor bHLH76 [Acc:Q9C670]	18
AT4G36980	0.078567844	-1.225905399	0.562460184	0.409845161	0.280457513	0.589868314	AT4G36980	function unknown	18
AT2G34930	-0.851813957	-1.226569225	-0.398617583	0.596579836	0.549775294	0.772504161	AT2G34930	Disease resistance family protein / LRR family protein [Acc:O64757]	18
AT5G18065	-0.419830996	-1.229887628	-0.102000153	0.215026885	-0.258616476	-0.165935433	HTT3	unknown protein	18
AT3G54880	-0.441648375	-1.248617444	-0.881337144	-0.375680626	-0.249396852	-0.665885718	AT3G54880	At3g54880 [Acc:Q9SV37]	18
AT5G20250	-0.883445324	-1.256028528	-0.326199622	-0.116560951	-0.570319703	-0.048156788	DIN10	Raffinose synthase family protein [Acc:F4K470]	18
AT5G41370	-0.42932002	-1.260681495	0.126182614	-0.134243957	-0.334143022	0.063330248	XPB1	General transcription and DNA repair factor IIH helicase subunit XPB1	18
AT1G30410	-0.234277721	-1.265282731	0.076894868	-0.594258772	-0.701980105	-0.224505725	ATMRP13	multidrug resistance-associated protein 13 [AT1G30410]	18
AT2G22920	-0.936293199	-1.266737473	-0.238245446	0	0	0	SCPL12	Serine carboxypeptidase-like 12 [Acc:O81009]	18
AT2G28470	-0.228016561	-1.283252953	-0.988068159	-0.071723508	0.38719221	-0.025722447	BGAL8	Beta-galactosidase 8 [Acc:Q9SCV4]	18
AT1G05830	-0.303598081	-1.28359376	0.094101977	0.045180892	-0.579715238	0.085813105	ATX2	Histone-lysine N-methyltransferase ATX2 [Acc:P0CB22]	18
AT3G16260	-0.236964785	-1.288356457	0.340712107	-0.254371534	-0.586712148	-0.090548828	TRZ4	TRZ4 [Acc:A0A178V7J3]	18
AT1G25098	-0.53643425	-1.291073387	0.474733192	0.387868072	0.21134155	0.563838285	AT1G25098	other RNA [AT1G25098]	18
AT1G27370	-0.251356933	-1.298192831	0.317681134	0.143957799	-0.357821059	0.226809547	SPL10	Squamosa promoter-binding-like protein 10 [Acc:Q8S9L0]	18
AT4G37610	-0.600784675	-1.308327692	-0.391653855	0.669163966	0.20183058	0.660241376	BT5	BTB/POZ and TAZ domain-containing protein 5 [Acc:Q6EJ98]	18
AT5G60430	0.110527139	-1.313362748	0.253111603	0.33126997	0.12262	0.250686518	AT5G60430	Antiporter/ drug transporter [Acc:Q9FKK3]	18
AT3G57470	-0.388157835	-1.347753447	0.46448883	0.027494064	-0.417128806	0.468019548	AT3G57470	Insulin-degrading enzyme-like 2 [Acc:F4J3D9]	18
AT4G20320	-0.690302279	-1.348860897	-0.547720556	0.227737565	-0.431330787	-0.84824107	AT4G20320	CTP synthase [Acc:Q0WRY1]	18
AT5G37690	-0.375289652	-1.357508273	-1.012599132	0	0	0	AT5G37690	GDSL esterase/lipase At5g37690 [Acc:Q9FHQ1]	18
AT4G33770	-0.862151434	-1.363502666	-0.86827962	-0.768461912	-0.313937185	-0.196541662	ITPK2	Inositol-tetrakisphosphate 1-kinase 2 [Acc:O81893]	18
AT5G48220	-0.024055834	-1.37007557	0.274020704	-0.059315943	-0.492095595	-0.108721447	AT5G48220	AT5g48220/MIF21_11 [Acc:Q9LUB2]	18
AT4G36670	-0.229498683	-1.381400363	-0.115506645	-0.365530961	-0.734831537	-0.148229909	PLT6	At4g36670 [Acc:A4VCM1]	18
AT3G49070	-0.792031248	-1.402873189	-0.963181762	0	0	0	AT3G49070	UPF0496 protein At3g49070 [Acc:Q9SMU4]	18
AT5G62630	-1.055276845	-1.405175821	-0.634601426	-0.478608459	-0.136472636	0.15261884	HIPL2	HIPL2 protein [Acc:Q94F08]	18
AT1G15040	-0.930923892	-1.422047917	-0.941633432	0.347288517	-0.608369313	-0.119197225	GAT1_2.1	Putative glutamine amidotransferase GAT1_2.1 [Acc:Q8H0Z4]	18
AT5G44750	-0.210504254	-1.432229679	0.101280321	0.071067993	-0.481785804	0.265203722	REV1	DNA repair protein REV1 [Acc:A3EWL3]	18
AT4G04740	-0.199916532	-1.436719747	0.228833272	-0.084658281	-0.026504158	0.373330254	CPK23	Calcium-dependent protein kinase 23 [Acc:F4JGW8]	18
AT1G30420	-0.651401829	-1.455009577	-0.782767145	-0.175445624	-1.128463199	0.112716751	ABCC11	ABC transporter C family member 11 [Acc:Q9C8H1]	18
AT1G19397	0.38741558	-1.467779541	0.684821805	0.083704923	0.412886549	-0.052599263	AT1G19397	unknown protein; Ha. [AT1G19397]	18
AT2G16750	0.144862374	-1.503601868	-0.294434537	0.50639256	0.306078258	0.811238748	AT2G16750	At2g16750 [Acc:B5X4Z9]	18
AT1G65370	-0.518287561	-1.529337091	0.213945432	-0.046161105	-0.068387411	0.178268254	AT1G65370	At1g65370/T8F5_15 [Acc:Q940M1]	18
AT4G15530	-0.615212193	-1.552979496	-0.049265531	-0.468056401	-0.330209999	-0.262357108	PPDK	Pyruvate, phosphate dikinase 1, chloroplastic [Acc:O23404]	18
AT1G13670	-1.10831229	-1.553235799	-1.230052558	-0.547292523	-0.569196484	-0.663907169	AT1G13670	At1g13670 [Acc:Q9LMY0]	18
AT1G54400	-1.049196262	-1.618958055	-1.113976518	0	0	0	AT1G54400	HSP20-like chaperones superfamily protein [Acc:F4HWW7]	18
AT4G03205	-0.329378465	-1.629765692	0.859419405	0.253245977	-0.510107836	0.420662991	hemf2	Coproporphyrinogen III oxidase [AT4G03205]	18
AT2G28570	-0.253477868	-1.640995844	0.042130099	0.368857136	-0.09916914	0.516948131	AT2G28570	At2g28570 [Acc:Q9SK01]	18
AT1G73300	0.114840089	-2.229979274	0.030513791	0	0	0	SCPL2	Serine carboxypeptidase-like 2 [Acc:Q9CAU3]	18
AT4G24415	-1.235462242	-2.246204201	-1.379516581	-0.244726104	0.213197859	0.34028154	MIR824A	MIR824a; miRNA [AT4G24415]	18
AT1G51270	-0.634105762	-2.408211575	-0.811253525	-0.61856725	-0.69860387	0.422084017	AT1G51270	Vesicle-associated protein 1-4 [Acc:F4I818]	18

AT2G28500	-1.931446754	-2.649576331	-2.130291847	1.88981566	1.223688019	0.992893708	LBD11	LOB domain-containing protein 11 [Acc:Q9SK08]	18
AT4G08795	0.331608132	-2.861223619	0.246408105	-0.934030122	0.271178773	0.081958021	AT4G08795	None	18
AT2G28760	-0.203768126	-0.242949132	-0.356253476	-1.030175709	-0.447403013	-1.020537323	UXS6	UDP-XYL synthase 6 [AT2G28760]	19
AT4G18440	-0.532758647	-0.089185014	-0.62668271	-1.053770305	-0.593626516	-1.286469608	AT4G18440	Adenylosuccinate lyase [Acc:Q8RY94]	19
AT1G13110	0.342887957	0.161483515	0.23048367	-1.107200162	-0.905106846	-1.377595335	CYP71B7	CYP71B7 [Acc:A0A178WGC6]	19
AT5G37600	0.224900009	0.243905087	-0.454076215	-1.128183587	-0.736674447	-1.478061931	GLN1-1	Glutamine synthetase cytosolic isozyme 1-1 [Acc:Q56WN1]	19
AT2G30010	-0.661320804	-0.060378958	0.546947022	-1.13763003	-0.338185135	-1.119951332	TBL45	TRICHOME BIREFRINGENCE-LIKE 45 [AT2G30010]	19
AT3G17340	-0.514326582	-0.993852892	-0.592525216	-1.15778098	-0.956961762	-1.033447819	AT3G17340	ARM repeat superfamily protein [Acc:F4J549]	19
AT1G17745	-0.541928514	-0.063090046	-0.702471727	-1.173499283	0.043849848	-1.249673957	PGDH	D-3-phosphoglycerate dehydrogenase [AT1G17745]	19
AT1G72610	1.025141964	-1.079445878	-1.774895924	-1.197543265	-0.865489372	-1.447365226	GLP1	Germin-like protein subfamily 3 member 1 [Acc:P94040]	19
AT1G52400	0.180914856	-0.122154051	0.202156553	-1.211041528	-0.154312592	-1.265528365	BGLU18	Beta-D-glucopyranosyl abscisate beta-glucosidase [Acc:Q9SE50]	19
AT1G07370	-0.422392776	0.285933723	-0.94949226	-1.27967683	-0.493886051	-1.063992463	PCNA	Proliferating cellular nuclear antigen 1 [Acc:Q9M7Q7]	19
AT1G06160	-0.725710659	0.096729224	-0.217763436	-1.38693022	-0.760824675	-1.670092294	ERF094	Ethylene-responsive transcription factor ERF094 [Acc:Q9LND1]	19
AT4G29110	0.081754682	0.479740681	-0.264001268	-1.676148827	-0.83133574	-1.943792227	AT4G29110	At4g29110 [Acc:Q9SZE0]	19
AT5G24660	-1.641998262	-0.326842421	-1.126180519	-2.518785591	0.037091492	-2.408099451	LSU2	Protein RESPONSE TO LOW SULFUR 2 [Acc:Q9FIR9]	19
AT1G05523	1.478466435	-9.64718417	-0.387021781	9.514091626	10.65908742	0	AT1G05523	None	20
AT5G03890	-0.043575257	0.536405078	-0.602783189	2.213387599	0.612133953	0.271384831	AT5G03890	Emb [Acc:Q9LZC0]	20
AT3G06345	0.80081213	1.704211088	-0.004366884	1.769168029	-0.125390823	-0.753232525	AT3G06345	None	20
AT1G74490	0.381950897	0.574550446	0.070141452	1.690453877	0.613878877	0.998740027	AT1G74490	At1g74490 [Acc:Q7Y224]	20
AT4G28230	-0.244418797	-0.083973696	-0.377552118	1.661774812	0.969646614	0.824894793	AT4G28230	At4g28230 [Acc:Q9M0I4]	20
AT5G44210	0.280577022	0.576034662	-0.721019001	1.628379354	1.077477369	0.763493548	ERF9	Ethylene-responsive transcription factor 9 [Acc:Q9FE67]	20
AT4G28703	-0.620976424	-0.10265163	-0.989169158	1.559649911	-0.316939622	-0.241058661	AT4G28703	RmlC-like cupins superfamily protein [Acc:Q8LAH4]	20
AT1G70420	-0.511981601	-0.109134946	-0.533982826	1.545374216	0.487127992	0.528373723	AT1G70420	At1g70420/F17O7_4 [Acc:O64594]	20
AT4G05010	0.251373268	0.047462166	-0.399366428	1.383582131	1.059915729	0.522441503	AT4G05010	F-box protein At4g05010 [Acc:Q9S9T6]	20
AT4G28720	0.359797997	0.122370257	-0.282381169	1.365786001	1.209315504	1.5066378	YUC8	Flavin-containing monooxygenase [Acc:A0A178V2K9]	20
AT2G19810	0.122995908	0.268474448	0.675775288	1.314563478	-0.162176205	0.769961069	AT2G19810	TZF2 [Acc:A0A178VX61]	20
AT5G63650	0.047768057	0.203868776	-0.245965355	1.309458711	0.997916784	0.958898482	SRK2H	Serine/threonine-protein kinase SRK2H [Acc:Q9FFP9]	20
AT1G23710	0.353499837	0.282479856	-0.157283405	1.274748333	0.474287063	0.916743415	AT1G23710	At1g23710 [Acc:Q9ZUC4]	20
AT5G62470	0.997798954	-0.435918989	-0.842436685	1.230949177	1.075834462	1.114872777	MYB96	Transcription factor MYB96 [Acc:Q24JK1]	20
AT1G32690	0.139870423	-0.013475805	-0.386289175	1.219140143	0.999609163	0.676498509	AT1G32690	At1g32690 [Acc:Q6NNH5]	20
AT4G33985	-0.03003211	0.31861146	-0.338256941	1.20933951	0.718667667	0.928019157	AT4G33985	Membrane insertase, putative (DUF1685) [Acc:Q0WS40]	20
AT5G66650	0.139261791	-0.72144705	-0.017189048	1.197684638	0.678527468	0.916382184	AT5G66650	Calcium uniporter protein 3, mitochondrial [Acc:Q9LVR5]	20
AT1G06227	0.139593863	0.150917953	-0.015042751	1.174487692	0.779834045	0.634176768	AT1G06227	None	20
AT3G29370	0.606365026	0.837959921	-0.59900464	1.163517534	0.928991214	0.788030829	AT3G29370	Uncharacterized protein At3g29370 [Acc:Q8LD48]	20
AT3G04640	0.304891282	0.666814878	0.00832808	1.148772812	0.976710222	0.650819197	AT3G04640	At3g04640 [Acc:Q9SR10]	20
AT1G21010	0.295652047	0.416506351	-0.078981538	1.133310717	0.888363209	0.512018366	AT1G21010	At1g21010 [Acc:Q7XJ58]	20
AT4G20840	-0.018182916	0.060845611	0.122852079	1.130113003	0.797760937	0.38881783	AT4G20840	Berberine bridge enzyme-like 21 [Acc:Q9SVG3]	20
AT5G19600	-0.62565054	-0.652855313	0.922541554	1.12173921	0.165117393	0.615415298	SULTR3;5	Probable sulfate transporter 3.5 [Acc:Q94LW6]	20
AT1G75860	0.167691313	-0.08697859	0.450621543	1.082923005	0.179014518	0.526129536	AT1G75860	unknown protein	20
AT1G56440	-0.060645776	-0.386174279	0.15229535	1.063855889	0.689033514	0.90103316	TPR5	TPR5 [Acc:A0A178WG02]	20
AT2G41070	0.212294032	0.42922432	-0.343042958	1.047397724	0.702534836	0.99922372	EEL	Basic-leucine zipper (bZIP) transcription factor family protein [AT2G41070]	20
AT3G60520	0.644770007	0.460077441	0.155524424	1.032106722	0.762404736	0.511932226	AT3G60520	At3g60520 [Acc:Q9M207]	20
AT3G62070	0.423611867	0.221012708	0.651142058	1.019468047	0.87315709	0.913772925	AT3G62070	At3g62070 [Acc:Q9M1R7]	20
AT4G18240	-0.117985157	-0.499815204	0.063827586	-0.349167483	-1.018024013	-0.383954968	SS4	Probable starch synthase 4, chloroplastic/amyloplastic [Acc:Q0WVX5]	21
AT5G20980	-0.444196419	-0.632836176	-0.372591156	-0.702767874	-1.019194874	-0.12162562	MS3	5-methyltetrahydropteroyltriglutamate--homocysteine methyltransferase 3	21
AT2G35650	-0.313901903	-0.376377697	-0.425689409	-0.208611613	-1.03828161	-0.438970144	CSLA7	Glucomannan 4-beta-mannosyltransferase 7 [Acc:Q9ZQN8]	21
AT5G53460	-0.299259925	-0.767690529	-0.726217072	-0.324633196	-1.046729065	-0.870037432	GLT1	Glutamate synthase 1 [NADH], chloroplastic [Acc:Q9LV03]	21
AT5G63960	-0.466001545	-0.817888267	-0.074249356	-0.705130452	-1.048586898	-0.40226065	AT5G63960	DNA polymerase [Acc:F4KC69]	21
AT4G19390	-0.640457529	-0.55856511	-0.007588268	-0.507953256	-1.066256352	-0.893979578	AT4G19390	AT4g19390/T5K18_170 [Acc:Q94JM4]	21
AT3G54750	-0.817503646	-0.864727256	-0.343912909	-0.819850009	-1.077609069	-0.877165805	AT3G54750	Downstream neighbor of Son [Acc:F4JE17]	21
AT3G63160	-0.014330744	-0.97157972	-2.492210132	-0.492869263	-1.089034995	-1.03860483	AT3G63160	OEP6 [Acc:A0A384KZL8]	21
AT2G41560	-0.006453722	-0.628017669	-0.364268687	-0.434270572	-1.108393703	-0.567222806	ACA4	Calcium-transporting ATPase 4, plasma membrane-type [Acc:O22218]	21
AT1G28330	0.207929955	-0.063839722	0.666553498	0.449981238	-1.118261065	-0.24681072	DRM1	Dormancy-associated protein 1 [Acc:B9DGG8]	21
AT2G29670	0.041294127	0.008472319	-0.052150044	-0.186240444	-1.126224818	-0.496957917	AT2G29670	At2g29670/T27A16.23 [Acc:O82388]	21
AT4G13505	-0.146163997	0.411456011	-0.311197484	-0.717364149	-1.129431243	-0.933885921	AT4G13505	other RNA [AT4G13505]	21
AT2G07680	-0.261417408	-0.527544476	-0.027478199	-0.46603909	-1.198870217	-0.888671928	ATMRP11	multidrug resistance-associated protein 11 [AT2G07680]	21
AT1G72940	-0.338477829	0.06379272	0.479418383	-0.559042172	-1.227062782	-0.767571925	AT1G72940	At1g72940/F3N23_14 [Acc:Q9SSN2]	21
AT1G08130	-0.476341605	-0.55800402	-0.446803532	-0.91290404	-1.242340365	-0.770881586	LIG1	DNA ligase 1 [Acc:Q42572]	21
AT5G63810	-0.030705705	-0.36974343	-0.231158943	-0.309673268	-1.258980325	-0.635086922	BGAL10	Beta-galactosidase 10 [Acc:Q9FN08]	21
AT5G24470	0.349568173	0.257408346	-0.111920614	0.171260439	-1.367136836	-0.172850142	APRR5	Two-component response regulator-like APRR5 [Acc:Q6LA42]	21
AT5G26570	-0.408360098	-0.488713649	0.011612522	-0.333434362	-1.382085847	-0.588256803	GWD3	Phosphoglucan, water dikinase, chloroplastic [Acc:Q6ZY51]	21
AT4G09020	-0.12712763	-0.549873719	0.020256874	-0.213703724	-1.386241488	-0.784875279	ISA3	Isoamylase 3, chloroplastic [Acc:Q9M0S5]	21
AT1G10760	-0.000962358	-0.555980061	-0.14599654	-0.541749846	-1.387213214	-0.997714286	GWD1	Alpha-glucan water dikinase 1, chloroplastic [Acc:Q9SAC6]	21
AT2G28900	-0.419770478	-0.151138245	-0.406407301	-0.583068634	-1.392169989	-0.886360913	OEP161	Outer envelope pore protein 16-1, chloroplastic [Acc:Q9ZV24]	21
AT1G69830	0.139118193	-0.120050844	0.649239589	-0.189675756	-1.610453758	-0.835770426	AMY3	Alpha-amylase 3, chloroplastic [Acc:Q94A41]	21
AT4G02800	-1.066080528	-0.71784024	-0.594039346	-0.295380082	-0.315873469	-0.554369821	AT4G02800	At4g02800 [Acc:Q9SY05]	22
AT3G13310	-1.08978758	-0.290694682	-0.628914719	-0.613593089	-0.451157764	-0.894445775	AT3G13310	At3g13310 [Acc:Q9LTT7]	22
AT5G08350	-1.138311775	-0.488091689	-0.561931482	0.040937952	0.340154286	0.426420485	AT5G08350	GEM-like protein 4 [Acc:Q9FTA0]	22
AT1G22885	-1.184696665	-0.7492116	-0.682139396	0.109792906	0.277394026	0.117389475	AT1G22885	unknown protein	22
AT3G20470	-1.26922958	0.131559492	-0.402410319	0.068019172	0.534772069	-0.713601896	GRP5	Glycine-rich protein 5 [Acc:Q9LTP5]	22
AT5G47240	-1.28549432	-0.774949607	-0.716290785	-1.137235167	-1.422416092	-1.217795304	atnudt8	nudix hydrolase homolog 8 [AT5G47240]	22
AT1G75000	-1.563527434	0.408225676	-1.030913909	0.521331733	0.151587596	1.244018551	AT1G75000	F25A4.4 [Acc:Q9S804]	22
AT1G11330	-1.625243545	-1.000497012	-0.311478631	0.010246179	-0.190170361	-0.432161301	AT1G11330	G-type lectin S-receptor-like serine/threonine-protein kinase [Acc:Q9SXB8]	22
AT4G30110	-1.910508501	-0.976765174	-1.011486358	-0.191312939	-0.066784091	-0.480980947	HMA2	Cadmium/zinc-transporting ATPase HMA2 [Acc:Q9SZW4]	22
AT5G06730	-2.129910414	-0.523685132	-0.411957028	0	0	0	PER54	Peroxidase 54 [Acc:Q9FG34]	22
AT4G16240	-3.731687724	-0.634148857	-1.40188441	-1.244991071	0.445814151	-0.749957567	AT4G16240	Glycine-rich protein homolog [Acc:O23471]	22
AT3G57520	-1.277966606	-0.881966208	-1.066850074	-1.205556403	-2.264355137	-1.550220754	RFS2	Probable galactinol--sucrose galactosyltransferase 2 [Acc:Q94A08]	23
AT5G40780	-0.608487996	-1.158153458	-1.085134052	-0.875657396	-1.090885872	-1.184341042	LHT1	Lysine histidine transporter 1 [Acc:Q9FKS8]	23
AT5G48310	-0.909400826	-1.109828232	-0.304805871	-0.608181923	-1.153302701	-1.147064318	AT5G48310	Portal protein [Acc:Q9LK83]	23
AT5G52450	-0.808998583	-0.698983967	-0.864246278	-0.905055378	-1.179162512	-1.057547965	DTX16	Protein DETOXIFICATION [Acc:A0A178UMN3]	23
AT5G07590	0.050898635	-0.071648479	-0.166371078	-0.481577746	-1.016785166	-1.121021091	AT5G07590	At5g07590 [Acc:Q5PP39]	23
AT2G33830	0.164343103	-0.036742168	0.929494059	-0.121146135	-1.525670401	-1.122376951	DRMH1	DRM2 [Acc:A0A178VR62]	23
AT5G11150	-0.546352441	-0.0618478	-0.909199082	-0.581783145	-1.008513679	-1.126045214	ATVAMP713	vesicle-associated membrane protein 713 [AT5G11150]	23
AT1G21130	-0.130353003	0.023062445	-0.350435717	-0.113538869	-1.215569386	-1.144567989	IGMT4	Indole glucosinolate O-methyltransferase 4 [Acc:Q9LPU8]	23
AT4G29700	-0.280000528	-0.538934357	-0.406769617	-0.652270062	-1.306002899	-1.174530355	AT4G29700	Alkaline-phosphatase-like family protein [Acc:Q9SU81]	23
AT4G36410	-0.442459688	-0.106317269	-0.566728157	-0.9569813	-1.044875901	-1.250852341	UBC17	Probable ubiquitin-conjugating enzyme E2 17 [Acc:O23239]	23
AT3G46970	-0.101364517	-0.191650109	0.052709531	-0.593826236	-1.948209898	-1.310548849	PHS2	Alpha-glucan phosphorylase 2, cytosolic [Acc:Q9SD76]	23
AT5G11670	-0.566176673	-0.605477138	-0.466707135	-0.617814542	-1.013281029	-1.33040473	NADP-ME2	NADP-dependent malic enzyme 2 [Acc:Q9LYG3]	23
AT2G01120	-0.581515118	-0.590723897	-0.180568857	-0.586453153	-1.343514199	-1.389937961	ORC4	Origin of replication complex subunit 4 [Acc:F4IM82]	23
AT1G15125	-0.640541436	-0.716559334	0.068849394	-0.999903891	-1.801525682	-1.869178659	AT1G15125	F9L1.6 [Acc:Q9XI57]	23
AT1G74310	-0.450200744	-0.621937728	-0.874137324	-1.208363614	-1.386395773	-2.004598601	CLPB1	Chaperone protein ClpB1 [Acc:P42730]	23
AT3G01545	1.361146575	2.07833804	-0.742261939	0.523930825	-8.246421091	0.930599919	AT3G01545	None	23
AT5G01600	1.080545965	0.690489915	1.425451171	1.384260629	1.041865038	0.864824446	FER1	Ferritin-1, chloroplastic [Acc:Q39101]	24
AT1G77410	1.737002614	1.205984962	1.693103455	0.645728784	0.179976478	0.863596881	BGAL16	beta-galactosidase 16 [AT1G77410]	24
AT3G47470	1.561164343	1.308576063	2.233887127	0.107518997	0.165692697	0.222721153	LHCA4	Chlorophyll a-b binding protein, chloroplastic [Acc:A0A178VEE6]	24
AT1G73750	1.406917175	0.309631231	1.23427483	0.781766327	1.269647587	0.958133822	AT1G73750	Uncharacterised conserved protein UCP031088, alpha/beta hydrolase	24
AT3G15400	1.248032435	0.975575345	1.331900238	0.579904192	0.707240205	0.824222899	ATA20	ATA20 protein [Acc:Q9LD84]	24
AT3G16770	1.152038153	0.778405235	1.926130794	0.520468161	0.706839835	0.300878873	RAP2-3	Ethylene-responsive transcription factor RAP2-3 [Acc:P42736]	24
AT5G47560	1.12018151	0.908814306	1.48134535	-0.183949635	0.049312446	0.098810232	TDT	Tonoplast dicarboxylate transporter [Acc:Q8LG88]	24
AT2G24545	1.108775199	0.847322788	1.094393663	0.158492796	0.74470449	-0.721852831	AT2G24545	other RNA [AT2G24545]	24
AT1G70890	1.061792285	0.525176663	1.149482989	-0.424490703	0.4748473	-0.565370222	MLP43	MLP-like protein 43 [Acc:Q9SSK5]	24
AT2G46830	1.033460949	0.191218833	1.720909008	-0.033355024	0.743720591	0.739676402	CCA1	Protein CCA1 [Acc:P92973]	24
AT1G44446	1.020162323	0.805074421	1.828590517	-0.313956394	-0.176153575	0.079555184	CAO	CH1 [Acc:A0A178W6L1]	24
AT4G11290	-0.236072527	-0.069274357	-1.055499452	-1.495898151	-0.891042105	-1.243963999	PER39	Peroxidase 39 [Acc:Q9SUT2]	25
AT1G43800	0.283288198	-0.455571122	-1.146867199	-3.830984783	-0.591510888	-1.816368882	S-ACP-DES6	Stearoyl-[acyl-carrier-protein] 9-desaturase 6, chloroplastic [Acc:Q84VY3]	25
AT4G23700	-0.542114155	-0.345231701	-1.224640774	-2.577589139	-0.839007269	-2.064808082	CHX17	Cation/H(+) antiporter 17 [Acc:Q9SUQ7]	25
AT2G46650	-1.047249967	-0.107029207	-1.410809682	-1.151063782	0.035910173	-0.614613656	CYTB5-C	Cytochrome B5 isoform C [Acc:Q9ZNV4]	25
AT5G14200	-1.034635478	-0.415341735	-1.633721332	-1.345229129	-0.09223132	-0.55247615	IMDH3	3-isopropylmalate dehydrogenase [Acc:Q5XF32]	25
AT3G54600	-1.065837455	-0.386335706	-1.664351417	-1.193905615	0.035260353	-0.499869474	DJ1F	DJ-1 protein homolog F [Acc:Q9M1G8]	25
AT4G13770	-1.279279277	-0.400673606	-1.727271329	-1.171816943	0.280342702	-0.374399105	CYP83A1	Cytochrome P450 83A1 [Acc:P48421]	25
AT1G10370	-0.435806014	-0.855785851	-2.072147447	-1.603536703	0.00684684	-1.283845828	GSTU17	Glutathione S-transferase U17 [Acc:Q9FUS8]	25
AT5G24760	-0.396450533	-0.366329917	-0.486862551	-1.027898477	-0.068284813	-0.662176846	AT5G24760	Alcohol dehydrogenase-like 6 [Acc:Q8LEB2]	25
AT5G63950	-0.509818851	-0.548652092	-0.442707064	-1.028535648	-0.847608046	-0.8900345	CHR24	Protein CHROMATIN REMODELING 24 [Acc:Q8W103]	25
AT1G78370	-1.008292361	-0.382897977	-1.27950902	-1.030379818	-0.312113395	-0.941910742	GSTU20	Glutathione S-transferase U20 [Acc:Q8L7C9]	25
AT2G46450	-0.216194443	-0.562843617	0.458595079	-1.030763575	-0.371708233	-0.63966194	CNGC12	Probable cyclic nucleotide-gated ion channel 12 [Acc:Q8GWD2]	25
AT2G07690	-0.419222023	-0.15704848	-0.324763955	-1.034414661	-0.749833132	-0.803183537	MCM5	DNA replication licensing factor MCM5 [Acc:O80786]	25
AT3G16470	-0.523916021	-0.455379342	0.246210584	-1.038723024	0.218127423	-0.93317309	JAL35	Jacalin-related lectin 35 [Acc:O04309]	25
AT5G64850	0.359782385	0.185883809	-0.042722795	-1.100269056	-0.397888087	-0.621321106	AT5G64850	At5g64850 [Acc:Q0WN19]	25
AT3G52430	-0.19172149	0.013748474	-0.109475764	-1.140845816	-0.652676703	-0.597680928	PAD4	Lipase-like PAD4 [Acc:Q9S745]	25
AT2G16440	-0.37990899	-0.279944704	-0.418488806	-1.141119698	-0.813464948	-0.690194381	MCM4	DNA replication licensing factor MCM4 [Acc:Q0WVF5]	25
AT1G77990	0.752958375	-0.259879831	0.800997083	-1.154034946	-0.775266553	-0.627129882	SULTR2;2	Sulfate transporter 2.2 [Acc:P92946]	25
AT2G43100	-1.231627696	-0.318271002	-1.417833657	-1.154919589	0.014837559	-0.481411768	IPMI2	IPMI2 [Acc:A0A178VZE1]	25
AT5G20830	0.418291545	0.124404885	0.944965375	-1.195914782	-0.242029266	-0.731636072	SUS1	Sucrose synthase 1 [Acc:P49040]	25
AT5G46280	-0.32232666	-0.290759717	-0.613305606	-1.24094253	-0.840873466	-0.99693771	MCM3	DNA replication licensing factor MCM3 [Acc:Q9FL33]	25
AT2G31380	-0.391099376	-0.363480773	0.136233513	-1.327286975	-0.556630009	-0.834318774	BBX25	B-box zinc finger protein 25 [Acc:Q9SID1]	25
AT1G44900	-0.372323935	-0.163049107	-0.687690789	-1.372432278	-0.820609124	-0.967783677	MCM2	DNA replication licensing factor MCM2 [Acc:Q9LPD9]	25
AT5G44635	-0.620283983	-0.268797931	-0.847597038	-1.390187262	-0.858976845	-0.788978678	MCM6	minichromosome maintenance (MCM2/3/5) family protein [AT5G44635]	25
AT3G20370	-0.18266737	-0.016580261	-0.849690389	-1.411771113	0.193319088	-1.017787587	AT3G20370	AT3g20370/MQC12_13 [Acc:Q9LTQ5]	25
AT5G55050	0.013698178	0.20967365	-0.558330039	-2.047019315	-0.555251288	-1.453016838	AT5G55050	GDSL esterase/lipase At5g55050 [Acc:Q9FIA1]	25
AT5G24090	0.248933225	0.174571953	-0.110502434	-2.39606962	0.465524433	-0.240640932	CHIB1	Acidic endochitinase [Acc:P19172]	25
AT2G38240	-3.05729537	-2.535844377	-2.609536211	-4.657044246	-1.414933252	-3.214975557	ANS	Probable 2-oxoglutarate-dependent dioxygenase ANS [Acc:O80449]	25
AT5G66780	1.631016481	1.449416696	-0.129590901	0	0	0	AT5G66780	AT5g66780/MUD21_2 [Acc:Q9FL02]	26
AT3G02535	1.539090828	1.743924388	0.778359725	-0.141232438	0.538159362	-0.290117023	AT3G02535	None	26
AT1G56300	1.485821107	1.402647065	0.85012188	0.899996809	0.013399181	0.097641937	AT1G56300	At1g56300 [Acc:Q8L7R1]	26
AT5G67488	1.448369432	1.72164818	-0.233614023	0.218923514	-0.137210403	-0.13286324	AT5G67488	other RNA [AT5G67488]	26
AT5G09978	1.358982968	1.129875066	0.089971416	0	0	0	PEP7	Elicitor peptide 7 [Acc:P0C1T5]	26
AT4G27310	1.352593912	1.356562827	0.84458831	0.744939416	0.557917529	0.58766355	AT4G27310	BBX28 [Acc:A0A178V4D3]	26
AT2G36210	1.304920331	1.067659063	0.78484154	0	0	0	AT2G36210	SAUR-like auxin-responsive protein family [Acc:Q8L8B9]	26
AT3G10020	1.287355591	1.270572159	0.992540469	0.588901699	0.561427302	0.490186864	AT3G10020	AT3g10020/T22K18_16 [Acc:Q9SR67]	26
AT5G23950	1.260541184	1.122348344	0.24523269	-0.896248479	2.160449124	0.560018488	AT5G23950	Calcium-dependent lipid-binding (CaLB domain) family [Acc:Q9FF85]	26
AT1G75750	1.238047988	1.147583828	0.946729821	0.446245518	0.884482718	-0.321443921	GASA1	GASA1 [Acc:A0A178W4S5]	26
AT4G04125	1.130471528	1.638556758	0.023596323	0.553582789	0.229065042	0.002091708	AT4G04125	None	26
AT5G01815	1.125837835	1.900913764	0.28597238	0.305230158	0.339378655	0.157768116	AT5G01815	None	26
AT3G48020	1.074764718	1.463699368	0.589107994	0.317668428	1.132497012	-0.1718141	AT3G48020	At3g48020 [Acc:Q9SU65]	26
AT1G80240	1.024524654	1.008564461	-0.187395671	0.322271911	0.342596305	-0.344462648	AT1G80240	At1g80240 [Acc:Q9SSB7]	26
AT2G39380	1.006683805	1.038040767	0.879923347	-3.407579765	-9.268494408	-1.996903967	ATEXO70H2	Exocyst subunit Exo70 family protein [Acc:O80625]	26

ROS core groups

- I The genome uncoupled (gun) mutation retrograde signaling
- II Long high light exposure
- III Short high light exposure
- IV Ozone/hydrogen peroxide/superoxide
- V Methyl viologen/antimycin A/oligomycin/dibromothymoquinone/N-octyl-3-nitro-2,4,6-trihydroxybenzamide/flg22 peptide
- VI Singlet oxygen/UV-B early
- VII The impact of RBOHF during oxidative stress
- VIII ROS acclimation

Supplementary Table 3. Gene families that are significantly enriched in each t-SNE cluster of DEGs between flight and ground control in Arabidopsis roots or leaves from human-tended VG suborbital spaceflight. Threshold of significant enrichment is FDR Padj < 0.01.

t-SNE cluster	Gene family	Short name	Padj
Cluster 0	None		
Cluster 1	Basic helix-loop-helix (bHLH) gene family gene family	bHLH	8.88E-04
Cluster 2	None		
Cluster 3	Core histone (CH) gene family	CH	4.01E-09
Cluster 4	Glutathione S-transferase (GST) gene family	GST	4.42E-04
	Cytochrome P450 gene family	CYP	3.91E-03
Cluster 5	FAD-binding Berberine (FBBE) gene family	FBBE	7.61E-03
Cluster 6	HSP90 gene family	HSP90	7.08E-04
	Heat shock factor (HSF) gene family	HSF	4.21E-03
Cluster 7	Chlorophyll a/b-binding (LHC) gene family	LHC	1.82E-05
Cluster 8	None		
Cluster 9	Glutathione S-transferase (GST) gene family	GST	1.89E-04
Cluster 10	None		
Cluster 11	Arabidopsis response regulators (ARR) gene family	ARR	8.75E-03
	Dof gene family	Dof	8.75E-03
Cluster 12	None		
Cluster 13	None		
Cluster 14	None		
Cluster 15	None		
Cluster 16	HSP20 gene family	HSP20	3.27E-21
Cluster 17	Heat shock factor (HSF) gene family	HSF	4.97E-05
	Caffeic acid o-methyltransferase (COMT) gene family	COMT	1.60E-03
Cluster 18	TAZ gene family	TAZ	1.04E-04
	CTP synthase (CTPS) gene family	CTPS	1.89E-03
Cluster 19	None		
Cluster 20	None		
Cluster 21	None		
Cluster 22	None		
Cluster 23	None		
Cluster 24	None		
Cluster 25	None		
Cluster 26	None		
